# Experimental Treatment of Ebola Virus Disease with Brincidofovir

**DOI:** 10.1371/journal.pone.0162199

**Published:** 2016-09-09

**Authors:** Jake Dunning, Stephen B. Kennedy, Annick Antierens, John Whitehead, Iza Ciglenecki, Gail Carson, Rupa Kanapathipillai, Lyndsey Castle, Rebecca Howell-Jones, Raul Pardinaz-Solis, Jennifer Grove, Janet Scott, Trudie Lang, Piero Olliaro, Peter W. Horby, for the RAPIDE-BCV trial team

**Affiliations:** 1 Centre for Tropical Medicine and Global Health, Nuffield Department of Clinical Medicine, University of Oxford, Oxford, United Kingdom; 2 University of Liberia-Pacific Institute for Research and Evaluation (UL-PIRE) Africa Center, University of Liberia, Monrovia, Liberia; 3 Médecins Sans Frontières, Operational Centre, Brussels, Belgium; 4 Department of Mathematics and Statistics, Lancaster University, Lancaster, United Kingdom; 5 Médecins Sans Frontières, Operational Center, Geneva, Switzerland; 6 Institute of Translational Medicine, University of Liverpool, Liverpool, United Kingdom; 7 UNICEF-UNDP-World Bank-WHO Special Programme for Research and Training in Tropical Diseases, Geneva, Switzerland; National Cancer Institute, UNITED STATES

## Abstract

**Background:**

The nucleotide analogue brincidofovir was developed to prevent and treat infections caused by double-stranded DNA viruses. Based on *in vitro* data suggesting an antiviral effect against Ebola virus, brincidofovir was included in the World Health Organisation list of agents that should be prioritised for clinical evaluation in patients with Ebola virus disease (EVD) during the West African epidemic.

**Methods and Findings:**

In this single-arm phase 2 trial conducted in Liberia, patients with laboratory-confirmed EVD (two months of age or older, enrolment bodyweight ≥50 kg) received oral brincidofovir 200 mg as a loading dose on day 0, followed by 100 mg brincidofovir on days 3, 7, 10, and 14. Bodyweight-adjusted dosing was used for patients weighing <50 kg at enrolment. The primary outcome was survival at Day 14 after the first dose of brincidofovir.

Four patients were enrolled between 01 January 2015 and 31 January 2015. The trial was stopped following the decision by the manufacturer to terminate their program of development of brincidofovir for EVD. No Serious Adverse Reactions or Suspected Unexpected Serious Adverse Reactions were identified. All enrolled subjects died of an illness consistent with EVD.

**Conclusions:**

Due to the small sample size it was not possible to determine the efficacy of brincidofovir for the treatment of EVD. The premature termination of the trial highlights the need to establish better practices for preclinical *in-vitro* and animal screening of therapeutics for potentially emerging epidemic infectious diseases prior to their use in patients.

**Trial Registration:**

Pan African Clinical Trials Registry PACTR201411000939962

## Introduction

Prompted by the worsening Ebola virus epidemic in West Africa the World Health Organisation (WHO) published an Emergency Use Assessment and Listing procedure in September 2014 that identified a mechanism and candidate agents for therapeutic evaluation in patients with Ebola virus disease (EVD) [1]. Brincidofovir (Chimerix Inc., Durham, North Carolina, USA), an orally-available lipid conjugate of the licensed nucleotide analogue cidofovir, was included in the initial list of agents prioritised for testing in clinical trials [2]. Brincidofovir has broad-spectrum activity against double-stranded DNA (dsDNA) viruses and was developed for the treatment of severe infections including cytomegalovirus and adenovirus, and as a medical countermeasure against smallpox [3]. For dsDNA viruses the active metabolite is cidofovir diphospate (CDV-PP), which acts as an intra-cellular competitive substrate for the viral DNA polymerase [4]. The antiviral activity of brincidofovir against Ebola virus (EBOV; a single-stranded RNA virus) was indicated by *in vitro* studies of virus replication performed in 2014 by the United States Centers for Disease Control and Prevention [2]. In October 2014 the United States Food and Drug Administration (FDA) authorised brincidofovir for compassionate treatment of patients with EVD through an emergency Investigational New Drug application (eIND), and a phase 2 single-arm study was registered in the United States (NCT02271347).

Although the mechanism of action of brincidofovir in inhibiting replication of EBOV in vitro was not known at the time, the decision to undertake this trial was made in the light of continued support for the prioritization of brincidofovir by the WHO Working Group on Ebola Therapeutics, the knowledge that experiments in non-human primate (NHP) models of EVD could not provide efficacy data since brincidofovir is metabolized differently by NHPs, and the fact that brincidofovir was in late stage clinical development for other indications with safety data available in >1000 subjects, some with life-threatening infections [5]. The prioritisation of brincidofovir for evaluation in the 2014–16 West Africa EVD outbreak was also supported by: oral dosing; low pill burden; product stability; immediate availability for use in clinical trials; and potential scalability of production.

The Rapid Assessment of Potential Drugs and Interventions for Ebola (RAPIDE) clinical trial platform was developed to assess potential treatments for EVD. The platform follows a multi-stage approach described elsewhere [6]. This report describes one component of this platform, the RAPIDE-brincidofovir trial, an open-label, non-randomised, single-arm trial of brincidofovir for EVD.

## Methods

### Study Setting

ELWA-3 (Eternal Love Winning Africa-3) Ebola treatment unit at the Ministry of Health compound in Monrovia, Liberia, operated by Médecins Sans Frontières.

### Study Design

An open-label, non-randomised, phase 2 single arm trial. Enrolled patients received brincidofovir in addition to standard supportive care. The primary outcome measure was survival, assessed at 14 days after the first dose. The full protocol is available in the Supporting Information (S1 File).

### Statistical analysis plan

The RAPIDE-BCV trial was developed at the peak of the Ebola virus outbreak in 2014 as a component of a research platform designed to evaluate a series of potential treatments as they became available [6]. Brincidofovir would first be evaluated in a phase 2 trial with a maximum of 140 patients without a concurrent control group, designed as a triage to classify the treatment as (a) very effective, (b) promising or (c) apparently ineffective. Further details of the statistical methods are available in the Supporting Information (S1 and S2 Files). The need to perform further trials and the trial design(s) required would be informed by the result of this phase 2 trial [6]. The sequential data monitoring and primary outcome analysis was to be based on outcomes in adult participants only.

### Eligibility criteria and consent

Patients aged two months of age or older who were first diagnosed with EBOV infection by reverse transcription polymerase chain reaction (RT-PCR) in the preceding 48 hours were eligible for inclusion. Women who were lactating had to agree to stop breastfeeding. Sexually-active participants had to agree to use condoms for 3 months following discharge. All female patients aged 15–49 years underwent pregnancy testing (blood or urine beta-HCG) and pregnant women were ineligible for the trial as embryotoxicity was detected in animal studies of brincidofovir. Other exclusion criteria were: an underlying disease or condition that could jeopardize the safety of the participant or others, or meant that the patient was unable to comply with protocol requirements; inability to swallow; or known severe renal impairment, defined as an estimated glomerular filtration rate (eGFR) <15 mL/min/1.73m^2^. Written informed consent was obtained directly from competent adults, and from appropriate representatives of participants <18 years of age or those who were too sick to consent.

### Study treatment

A dosing regimen was selected based on the half maximal effective concentration (EC50) against the Mayinga strain of Ebola Zaire in Huh7 cells, which were within the range of EC50s observed for dsDNA viruses which had been effectively treated with brincidofovir in animal models and humans, and the pharmacokinetics of brincidofovir in humans [3, 5, 7–10]. Patients with bodyweight ≥50 kg at enrolment received 200 mg loading dose of brincidofovir on day 0, followed by 100 mg brincidofovir on days 3, 7, 10, and 14. Patients with bodyweight <50 kg received 4 mg/kg brincidofovir as an initial dose on day 0, followed by 2 mg/kg on days 3, 7, 10, and 14. Brincidofovir was administered orally, as tablets or dissolved in water. All patients continued to receive supportive care in accordance with Médecins Sans Frontières EVD treatment guidelines (including intravenous crystalloids or oral rehydration solution, antimicrobials, antimalarials, analgesics, and nutritional support as prescribed by their treating clinician).

### Safety reporting

Serious Adverse Reactions (SARs), Suspected Unexpected Serious Adverse Reaction (SUSARs), and key adverse events were assessed from the first dose to the primary outcome (day 14). Severity of vomiting and diarrhoea were categorised subjectively by clinical staff as mild, moderate, or severe, since volumes of excreta and vomitus could not be measured reliably.

### Laboratory methods

Laboratory tests were limited to those requested by treating physicians. At the time of the trial, local clinical diagnostic laboratories did not have the operational capacity to perform additional assays for research purposes. EBOV RT-PCR assays were performed by the CDC/NIH laboratory associated with the ELWA-3 treatment unit. Haematology and biochemistry assays were conducted on lithium-heparin plasma or whole blood using i-STAT® (Abbott Point of Care Inc., Princeton, NJ, USA) and/or Piccolo Xpress® (Abbott Point of Care Inc., Princeton, NJ, USA) point of care assays.

### Ethics statement

The trial protocol was approved by the following research ethics committees: University of Liberia—Pacific Institute for Research and Evaluation Institutional Review Board, Monrovia, Liberia; Oxford Tropical Research Ethics Committee, University of Oxford, UK; Médecins Sans Frontières Ethics Review Board, Geneva, Switzerland. Additional ethics advice was received from the WHO Ethics Review Committee. Approval to conduct the trial was granted by the Liberia Medicines and Health Products Regulatory Authority (LMHRA) and the Ministry of Health and Social Welfare, Liberia. An Independent Data Monitoring Committee reviewed data on a sequential basis, and reviewed any SARs, SUSARs, or other safety concerns. Community awareness and engagement programmes were conducted by the University of Oxford and Médecins Sans Frontières, supported by national collaborators and authorities. The study was registered with the Pan African Clinical Trials Registry (PACTR201411000939962). The trial was conducted in compliance with the International Conference on Harmonisation Guidance on Good Clinical Practice (GCP), for which the LMHRA conducted compliance monitoring during the trial.

## Results

**Fig 1:**
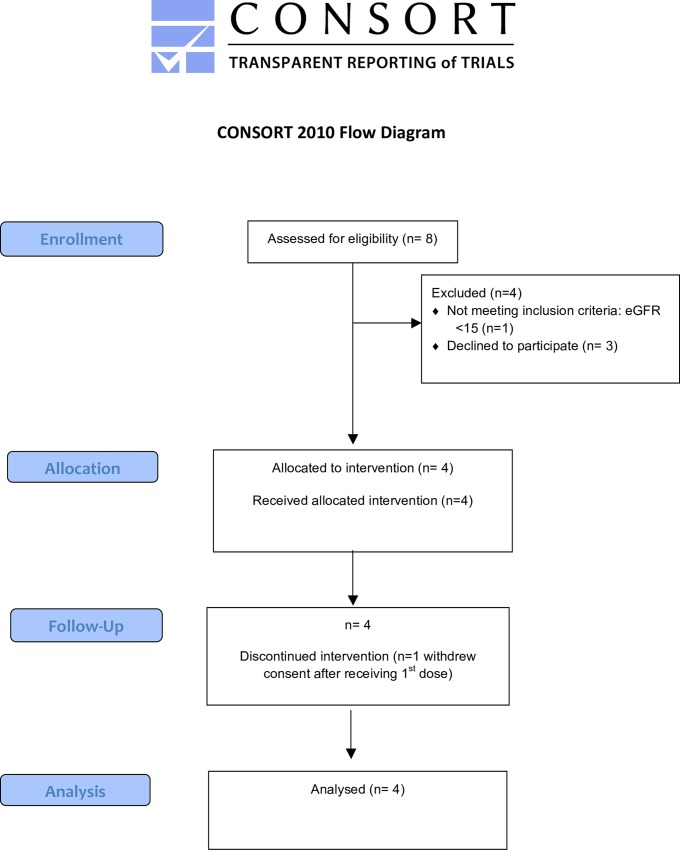
CONSORT Flow Diagram. Consort diagram of patients eligible, recruited, numbers followed up and included in analysis.

Four previously healthy participants with EVD were enrolled (two adults and two children) between 01 January 2015 and 31 January 2015 (Table 1). The CONSORT flow diagram is shown in Fig 1. On admission, all four participants had at least one feature associated with a high risk of death [11–15]. Participants 1 and 4 had haemorrhagic symptoms, and Participants 2 and 3 had Ebola virus RT-PCR cycle threshold values of 20. On admission, Participant 2 had evidence of hepatic injury (alanine aminotransferase concentration 750 U/L) and Participant 3 had evidence of dehydration (high haematocrit and haemoglobin) and renal impairment. Participant 4, a child, was enrolled with consent provided by a family member, which was withdrawn on day 2 of treatment by a more senior family member. The trial closed to recruitment on 31 January 2015 when the manufacturer ceased participation in all trials of brincidofovir for EVD.

**Table 1:** Demographic and clinical features at enrolment.

*At enrolment*	*Participant 1*	*Participant 2*	*Participant 3*	*Participant 4*
Age group (years)	20–29	10–14	40–49	10–14
Sex	F	M	F	F
Body Weight (kg)	54	37	65	28
Self reported duration of illness (days prior to enrolment)	3	7	2	3
EBOV RT-PCR cycle threshold value	22·70	20·66	20·80	26·74
Temperature (°C)	37·1	37·1	35·6	38·3
Heart rate (per minute)	104	102	112	98
Respiratory rate (per minute)	30	26	28	26
Systolic blood pressure (mmHg)	90	118	127	98
Diastolic blood pressure (mmHg)	65	75	96	56
HIV treatment in previous two weeks	No	No	No	No
Intravenous fluids in last 24 hours (volume in litres)	No	No	Yes (2·6)	Yes (1·5)
Oral rehydration fluids in last 24 hours	Yes	Yes	Yes	No
Malaria rapid test	Negative	Negative	Negative	Negative
Diarrhoea reported (severity*)	No	Yes (+)	Yes (+)	Yes(++)
Vomiting reported (severity*)	Yes (++)	Yes (+)	Yes (+)	No
Bleeding reported	Yes Yes (haematemesis)	No	No	Yes (epistaxis, Yes, (gingival)

* Physician assessed severity of vomiting and diarrhoea: + = mild; ++ = moderate; +++ = severe.

No patient completed the treatment course and none survived to day 14 (Table 2). Two participants received only the first dose: consent was withdrawn for Participant 4, and treatment was suspended for Participant 3 because of worsening pre-existing renal impairment (eGFR <15 mL/min/1·73 m^2^). Further clinical details of the participants are provided in the Supplementary Information (S3 File and S1 Table). All four deaths were consistent with severe EVD. Analysis of primary outcome data from the two adult patients was not undertaken because the sample size was insufficient to draw meaningful conclusions.

**Table 2:** Treatment and clinical progression during admission.

		Participant			
Description [normal range]	Study Day	1	2	3	4
Brincidofovir (weight adjusted dose)	Day 0	Y (200mg)	Y (150mg)	Y (200mg)	Y (110mg)
	Day 1	N	N	N	**Withdrew**
	Day 2	N	N	N	
	Day 3	Y (100mg)	Y (75mg)	N / **Died**	
	Day 4	N	N		
	Day 5	N	N		
	Day 6	N	N		
	Day 7	Y (100mg) / **Died**	N / **Died**		

No Serious Adverse Reactions or Suspected Unexpected Serious Adverse Reactions were identified. However the scheduled administration of the day 7 dose of brincidofovir was suspended for 24 hours in Participant 2 because of concerns that brincidofovir might be contributing to persistent diarrhoea. The patient died before a decision to continue the study drug could be made.

## Discussion

We report our experience of conducting a trial of brincidofovir for the treatment of EVD. We were unable to complete the trial or draw conclusions regarding drug efficacy due to slow recruitment (caused by falling case numbers in Liberia) and by the decision of Chimerix, Inc. to terminate their program of development of brincidofovir for EVD and to terminate the clinical trial agreement.

All four patients died, with illnesses consistent with severe EVD. There were no SARs or SUSARs reported. There was a possible temporal association between brincidofovir administration and worsening diarrhoea in one patient. Diarrhoea is the main dose-limiting toxicity observed with brincidofovir in other conditions but is most common after two weeks of therapy (personal communication Chimerix, Inc.)[16]. Since diarrhoea is very common in EVD and large volume watery diarrhoea is a reported in severe EVD, it is difficult to differentiate between diarrhoea as a symptom of EVD and diarrhea due to brincidofovir administration [15, 17–21].

Our study highlights the substantial uncertainties associated with the conduct of a trial of an experimental therapeutic under emergency conditions. Data on potential therapeutics were accruing during the trial set up and it was necessary to convene a special meeting of the WHO Working Group on Ebola Therapeutics in December 2014 (prior to initiating recruitment) to provide an expert opinion on the interpretation of new animal data for brincidofovir. The Working Group concluded that the new results were inconclusive and brincidofovir should remain a priority candidate for testing in EVD patients. On 30 January 2015 Chimerix, Inc. suspended the development of brincidofovir for EVD and withdrew from the trial [22]. On 18th February 2015, after the trial had closed to recruitment, brincidofovir was deprioritised by WHO based on new data bringing into doubt its anti-EBOV activity [1]. Prior to this change of status, brincidofovir had been given under an eIND to five medically evacuated EVD patients [18, 23], was registered for a phase 2 trial in the United States, and was used in our trial in Liberia. Subsequent work has identified that the lipid-nucleotide conjugate rather than the nucleotide itself is responsible for the inhibition of EBOV replication in cell culture [7]. Since the plasma half-life of the lipid-nucleotide conjugate is much shorter than the intra-cellular half-life of CDV-PP, the brincidofovir dose and schedule used on a compassionate basis, in animal studies, and in this trial is likely to have been insufficient to inhibit EBOV replication since dosing was based on the assumption that CDV-PP was the active agent. The inconsistent pre-clinical data, and differing opinions on their interpretation, highlight the need to develop and agree better practices for preclinical *in-vitro* and animal screening of therapeutics for potentially emerging epidemic infectious disease prior to their use in patients. In the absence of evidence of clinical effectiveness in a validated animal model of infection, it is vital that the mechanism of action and the pharmacokinetics of the active moiety are established. It is also important that when data are emerging and opinion is divided during a public health emergency, both the data and the differing opinions about its interpretation are made readily available so that clinicians can reach informed decisions about whether to proceed with clinical studies. This will maximise the likelihood of identifying effective treatments and will reduce the risk and potential associated harms of failed trials.

## Supporting Information

S1 File Trial protocol and statistical analysis plan. (PDF)

S2 File Statistical methods. (DOCX)

S3 File Additional clinical details of trial participants. (DOCX)

S4 File CONSORT Checklist. (DOC)

S1 Table Treatment and clinical progression during admission. (DOCX)
