# Supplementary material for: Experimental Treatment of Ebola Virus Disease with Brincidofovir
Source: PLoS One. 2016 Sep 9;11(9):e0162199. doi: 10.1371/journal.pone.0162199 (PMC5017617; doi:10.1371/journal.pone.0162199)
Supplement: S1 File — (PDF) [file pone.0162199.s001.pdf]

This supplement contains the following items:

1. Original protocol, final protocol, summary of changes.

2. Original statistical analysis plan

**Trial Title: Rapid Assessment of Potential Interventions & Drugs for Ebola (RAPIDE) - BCV**

**Scientific Title:** Open-label, non-randomised single arm trial to investigate the efficacy of Brincidofovir compared to historic controls for Ebolavirus Disease in an outbreak setting in West Africa.

**Short title:** Evaluation of BCV Treatment for Ebolavirus Disease

**Date and Version No**: 5th November 2014, V2.1


| | |
|---|---|
| **Chief Investigator:** | **Peter Horby**<br>Centre for Tropical Medicine and Global Health<br>Nuffield Department of Medicine Research Building<br>University of Oxford<br>Old Road Campus, Roosevelt Drive<br>OXFORD, OX3 7FZ |
| **Country Principal Investigator:** | **Stephen Kennedy**<br>Pacific Institute for Research and Evaluation<br>University of Liberia<br>Capitol Hill<br>Monrovia, Liberia |
| **Co-Investigators:** | **Micaela Serafini** Medical director of the MSF Operational Center Geneva, Médecins Sans Frontières – Doctors without Borders (MSF)<br><br>**Bertrand Draguez** Medical Director, MSF Operational Center Brussels, MSF<br><br>**Laboratory Lead,** to be determined<br><br>**Trudie Lang** Global Health Network, Centre for Tropical Medicine and Global Health, University of Oxford<br><br>**Piero L Olliaro** Newton-Abraham Visiting Professor, University of Oxford.<br><br>**John Whitehead** Department of Mathematics and Statistics, Lancaster University |
| **Sponsor:** | University of Oxford<br><br>Joint Research Office, Block 60, Churchill Hospital, Oxford, OX4 7LE |



| | |
|---|---|
| **Funder:** | Wellcome Trust |

## TABLE OF CONTENTS

1. KEY TRIAL CONTACTS ........ 4
2. SYNOPSIS ........ 6
3. ABBREVIATIONS ........ 7
4. INTRODUCTION ........ 7
5. OBJECTIVES AND OUTCOME MEASURES ........ 9
6. TRIAL DESIGN ........ 10
7. PARTICIPANT IDENTIFICATION ........ 12
   7.1. Trial Participants ........ 12
   7.2. Inclusion Criteria ........ 12
   7.3. Exclusion Criteria ........ 12
8. TRIAL PROCEDURES ........ 12
   8.1. Screening ........ 12
   8.2. Informed Consent ........ 13
   8.3. Assessments ........ 13
   8.4. Follow Up Assessments (Day 14 and Day 30) ........ 13
   8.5. Laboratory Assessments ........ 14
   8.6. Discontinuation of Trial Treatment ........ 15
   8.7. Withdrawal from the Study ........ 15
9. Trial Therapy ........ 15
   9.1. Investigational Medicinal Product ........ 15
   9.2. Dosing ........ 15
   9.3. Treatment Interruption ........ 16
   9.4. Storage and Accountability ........ 16
   9.5. Supportive Therapy ........ 16
10. SAFETY REPORTING ........ 16
   10.1. Procedures for safety reporting ........ 17
11. STATISTICS ........ 18
   11.1. Description of Statistical Methods ........ 18
   11.1. The Level of Statistical Significance ........ 19
   11.2. The Number of Participants ........ 19
   11.3. Criteria for the Termination of the Trial ........ 19
   11.4. Final Analysis ........ 21

11.5. Inclusion in Analysis ........ 21
11.7 Procedure for Accounting for Missing, Unused, and Spurious Data. ........ 21
11.8 Rationale for statistical design choice ........ 21
12. DATA MANAGEMENT ........ 22
12.1. Data Capture ........ 22
12.2. Access to Data ........ 22
13. QUALITY ASSURANCE PROCEDURES ........ 22
14. ETHICAL AND REGULATORY CONSIDERATIONS ........ 23
14.1. Regulations, Guidelines and Approval ........ 23
14.2. Considerations in Informed Consent ........ 23
14.3. Participant Confidentiality ........ 25
14.4. Sample Management ........ 25
14.5. Risks and Benefits ........ 26
14.6. Expenses ........ 27
14.7. Contemporaneous Studies ........ 27
14.8. Alternatives to Study Participation ........ 27
14.9. Community Engagement ........ 27
14.10. Drug Access Post-Study ........ 28
15. FINANCE AND INSURANCE ........ 28
15.1. Funding ........ 28
15.2. Insurance ........ 28
16. PUBLICATION POLICY ........ 28
17. REFERENCES ........ 28
18. APPENDIX A: TRIAL FLOW CHART ........ 30
19. APPENDIX B: DOSING VOLUMES FOR DISOLVED TABLETS ........ 31
20. APPENDIX C: AMENDMENT HISTORY ........ 32

## 1. KEY TRIAL CONTACTS

| | |
|---|---|
| **Chief Investigator** | Associate Professor Peter Horby<br><br>Centre for Tropical Medicine and Global Health<br>Nuffield Department of Medicine Research Building<br>University of Oxford<br>Old Road Campus, Roosevelt Drive<br>OXFORD<br>OX3 7FZ<br><br>Tel: 07990 560237<br>Email: Peter.horby@ndm.ox.ac.uk |
| **Sponsor** | University of Oxford<br>Joint Research Office<br>Block 60, Churchill Hospital<br>Oxford, OX4 7LE |
| **Statistician** | Professor John Whitehead<br><br>Department of Mathematics and Statistics<br>Fylde College<br>Lancaster University<br>Lancaster LA1 4YF<br><br>Tel: 01524 389967<br>Email: j.whitehead@lancaster.ac.uk |
| **Committees** | **Trial Steering Committee Voting Members:**<br><br>**Samuel Kargbo** (Sierra Leone Representative)<br>Ministry of Health and Sanitation, Freetown, Sierra Leone<br><br>**Mandy Kader Konde** (Guinea Representative)<br>Commission Recherche Ebola, Conakry, Guinea<br><br>**Stephen Kennedy** (Liberia Representative)<br>Pacific Institute for Research and Evaluation, Monrovia, Liberia<br><br>**Fred Binka** (Independent Member)<br>University of Health and Allied Sciences, Ho, Ghana<br><br>**Peter Horby** (Chief Investigator)<br>Centre for Tropical Medicine and Global Health, Oxford, United Kingdom<br>International Severe Acute Respiratory and emerging Infections Consortium<br><br>**Annick Antierens** (MSF Representative)<br>Médecins Sans Frontières, Geneva, Switzerland<br><br>**John Whitehead** (Trial Statistician) |

| | |
|---|---|
| | Lancaster University, Lancaster, United Kingdom<br><br>**Oumou Younoussa Bah-Sow** (Independent Member)<br>Hopital National Ignace Deenm, Guinee<br><br>**Rob Fowler** (Trial Clinical Lead)<br>Sunnybrook Health Sciences Centre, Toronto, Canada<br><br>**Nicholas White** (Independent Member - Chairperson)<br>Mahidol-Oxford Research Unit, Bangkok, Thailand<br><br>**Non-Voting Members**<br>**Ana Maria Henao-Restrepo** (WHO Representative)<br>World Health Organization, Geneva, Switzerland<br><br>**Independent Data Monitoring Committee (IDMC) Members:**<br><br>**Donald Berry,** Founder and senior Statistical Scientist, Berry Consulting<br><br>**Pontiano Kaleebu,** Director MRC/UVRI Uganda Research Unit on AIDS – to be confirmed<br><br>**Clement Adebamowo,** Director Office of Strategic Information and Research Institute of Human Virology in Nigeria<br><br>**David Lalloo,** Professor of Tropical Medicine; Dean of Clinical Sciences and International Public Health, Liverpool School of Tropical Medicine – to be confirmed<br><br>**Nicolas Opoku,** Director Onchocerciasis Chemotherapy Research Centre (OCRC), Hohoe, Ghana<br><br>**Peter Smith,** Professor of Tropical Epidemiology, London School of Hygiene and Tropical Medicine – to be confirmed |

## 2. SYNOPSIS

| | | |
|---|---|---|
| Trial Title | **Rapid Assessment of Potential Interventions & Drugs for Ebola (RAPIDE) - BCV** | |
| Scientific Title | Open-label, non-randomised single arm trial to investigate the efficacy of Brincidofovir compared to historic controls or Ebolavirus Disease in an outbreak setting in West Africa | |
| Internal ref. no. (or short title) | Evaluation of BCV Treatment for Ebolavirus Disease | |
| Clinical Phase | 2 | |
| Trial Design | Open-label, non-randomised, single arm trial | |
| Trial Participants | Patients with confirmed Ebolavirus disease attending the participating treatment centre | |
| Planned Sample Size | Up to 140 adult participants, paediatric enrolment according to presentation over the same recruitment period | |
| Treatment duration | 2 weeks | |
| Follow up duration | 30 days follow-up | |
| | Objectives | Outcome Measures |
| Primary | To evaluate the impact of BCV treatment on early mortality in EVD | Mortality at Day 14 |
| Secondary | 1. To evaluate the impact of BCV treatment of adults and children on:<br>a) Time to recovery<br>b) Late mortality<br>c) Viral load<br>d) EVD symptoms<br>e) EVD antibody response<br><br>2. To assess the safety of BCV treatment of adults and children | 1. a) Time to meeting EVD treatment center discharge criteria.<br>b) Mortality at Day 30 after first dose of study treatment<br>c) Day 4 viral load<br>d) Presence and duration of symptoms (Days 1-14)<br>e) Convalescent anti-Ebolavirus IgG titer (Day 30)<br><br>2. Incidence of SARs and key adverse events (Days 1-14) |
| Investigational Medicinal Product | Brincidofovir (BCV) | |
| Formulation, Dose, Route of Administration | Patients ≥50 kg: 200 mg initial dose, then 100 mg twice weekly for four (4) doses<br>Patients <50 kg: 4mg/kg initial dose, then 2 mg/kg twice weekly for four (4) doses | |

## 3. ABBREVIATIONS

| | |
|---|---|
| BCV | Brincidofovir (BCV) |
| CRF | Case Report Form |
| EDTA | Ethylenediaminetetraacetic acid |
| ETC | Ebola Treatment Centre |
| EVD | Ebolavirus Disease |
| GCP | Good Clinical Practice |
| IB | Investigators Brochure |
| ICF | Informed Consent Form |
| ICH | International Conference on Harmonisation |
| IDMC | Independent Data Monitoring Committee |
| IMP | Investigational Medicinal Product |
| MSF | Medecins Sans Frontieres |
| OXTREC | Oxford Tropical Research Ethics Committee |
| PI | Principal Investigator |
| REC | Research Ethics Committee |
| RNA | Ribonucleic acid |
| RT-PCR | Reverse transcription polymerase chain reaction |
| SAE | Serious Adverse Event |
| SAR | Serious Adverse Reaction |
| SUSAR | Suspected Unexpected Serious Adverse Reactions |
| TSC | Trial Steering Committee |
| WHO | World Health Organisation |

## 4. INTRODUCTION

The size and scale of the on-going Ebola Virus Disease (EVD) outbreak is unprecedented, shows no signs of abating, and has been declared a Public Health Emergency of International Concern.[1] EVD is among the most virulent infectious agents known: an analysis of data on 3343 confirmed and 667 probable EVD cases collected during the current outbreak in Guinea, Liberia, Nigeria, and Sierra Leone estimates a case fatality rate of 70.8% (95% confidence interval [CI], 69 to 73) among persons with known clinical outcome of infection.[2] A figure that is consistent with estimates of other authors.[3] **Figure 1** shows the key time points in the course of EVD in this outbreak.[2]

**Figure 1. Estimated mean (median) time course in days of Ebolavirus infection in this outbreak.[2]**

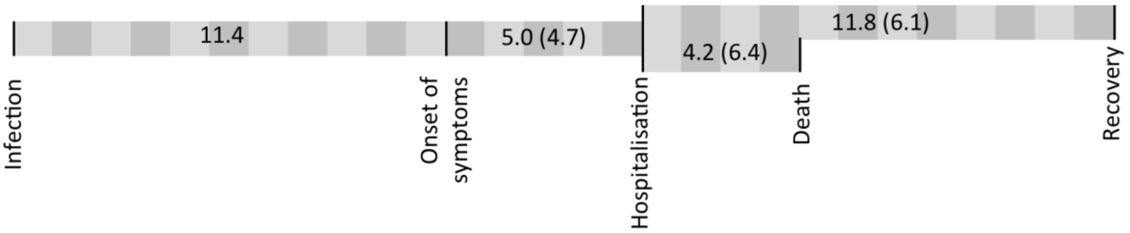


The current Ebolavirus strain causing the outbreak in West Africa is related to the Ebola Zaire strain and genetic analysis suggests the current outbreaks in Guinea, Sierra Leone and Liberia are all related to a single transmission event from a zoonotic source followed by human to human transmission.[4] EVD is characterized by a febrile illness dominated by fatigue and gastrointestinal symptoms that can be complicated by shock, haemorrhage and multi-organ failure. However, clinical presentation and severity is variable, with some patients remaining relatively well, able to ambulate and self feed throughout their illness, whilst others progress rapidly to a fatal outcome.[2, 5]
The pathogenesis of Ebola Virus Disease is incompletely understood [6, 7] but high levels of viral replication and the detection of virus in multiple body tissues is typical of severe.[8, 9] Coagulopathy, disruption of endothelial function and increased inflammatory responses are also associated with severe EVD.[6, 10-12] The association between high levels of viraemia and EVD severity suggests that therapies that target viral replication may benefit patients. Several experimental products that target Ebolavirus replication have show some efficacy in animal studies.[13-15] However, whilst several therapeutic interventions have shown promise in the laboratory and in animal studies, none have been tested for efficacy and safety in humans with EVD. A World Health Organization (WHO) expert panel recently concluded unanimously that "investigators have a moral duty to evaluate these interventions in the best possible clinical studies that can be conducted under the circumstances of the epidemic."[16]

The aim of this protocol is to assess the effectiveness of Brincidofovir (BCV, produced as CMX001 by Chimerix Inc.) for the treatment of EVD. BCV is an orally bioavailable, lipid acyclic nucleotide that was designed to utilize endogenous lipid uptake pathways to achieve high intracellular concentrations of BCV. BCV is a lipid conjugate of the nucleotide analogue cidofovir. BCV has demonstrated broad spectrum antiviral activity against various pathogenic human double stranded DNA (dsDNA) viruses in vitro and in animal models. BCV has successfully completed Phase 2 clinical development for the prevention of cytomegalovirus infection. A Phase 3 study of cytomegalovirus prevention in adult haematopoietic cell transplant recipients, Study CMX001-301 (also known as the SUPPRESS trial), began enrolling subjects in 2013. In addition, BCV is in Phase 2 clinical development for preemptive treatment of adenovirus viremia. In cytomegalovirus -infected haematopoietic cell transplant subjects, the main dose-limiting toxicity has been gastrointestinal in nature, primarily diarrhea, when administered at total weekly doses greater than 200 mg. In addition, dose-related, transient, asymptomatic serum aminotransferase increases (primarily alanine transaminase, but also aspartate transaminase) have been reported in both virally-infected subjects and healthy subjects, but do not appear to be of toxicologic importance. There is no evidence of the nephrotoxicity, myelotoxicity and ocular toxicity associated with the intravenous administration of cidofovir following once or twice weekly oral administration of BCV. The safety database of over 1000

individuals exposed to BCV has shown no evidence of hematologic toxicity or nephrotoxicity. No dose adjustment of BCV is necessary for renal or hepatic impairment. The potential for drug-drug interactions mediated through cytochrome P450 enzymes is low. BCV should be considered a potential carcinogen and should not be administered for longer than 3 months unless the potential benefit justifies the potential risk to patients.

BCV was tested for activity against the Mayinga strain of Ebola Zaire in Huh7 cells at the US Centers for Disease Control and Prevention. BCV was added to cells at 48, 24 and 2 hours prior to infection. The half maximal effective concentration (EC50) ranged from 21 nM with 48 hour pretreatment to 524 nM with a 2 hour pretreatment with therapeutic indices ranging from 54 to >2300. These EC50s encompass the range of EC50s observed for dsDNA viruses, which have been effectively treated with BCV in humans and animal models (adenovirus, poxviruses). Additional assessments of BCV in mouse models of Ebolavirus infection are underway. The pre-clinical work indicating potential efficacy combined with experience in over 1000 patients to show efficacy in other viral infections and providing safety and tolerability data, support the case for undertaking a phase IIB assessment of this drug in patients presenting with EVD.

Reproductive studies of BCV have identified embryotoxicity and fetal morphological changes in rabbits, and decreased fertility, embryonal viability, and growth and development of pups with delayed sexual maturation in rats. Given the current lack of data on the efficacy of BCV treatment in humans infected with Ebolavirus, the theoretical risk of embryotoxicity precludes the administration of BCV to pregnant women.

The Trial Steering Committee selected BCV for urgent evaluation from a possible list of 10 candidate therapies. The criteria for selection were based upon a Target Product Profile that considered existing clinical and pre-clinical data supporting safety and efficacy, immediate availability and scalability, and an acceptable treatment regimen.

The purpose of this trial is to establish whether BCV is (a) very effective, (b) promising or (c) ineffective. Details of how these three states are characterized and how BCV will be classified based on the data from this trial are given in Section 11 below. If conclusion (a) is reached, we recommend that the high effectiveness of BCV is confirmed in a subsequent single-arm trial of at least 100 patients. If conclusion (b) is reached, we recommend that the promise of BCV is studied further in a randomized comparison with best supportive care. If conclusion (c) is reached, we recommend that other treatments are given priority in clinical research, but that BCV might be reconsidered if better treatments are not found. We are developing trial designs for suitable single-arm confirmatory and randomized comparative trials that will ensure low risks that the sequence of drug development will give invalid conclusions.

It is the intention to use this clinical trial to build a platform for investigation of a number of experimental interventions for the treatment of EVD. As additional therapies become available, and are deemed appropriate for evaluation, this protocol will be replicated to test additional investigational medicinal products in multiple single arm studies.

## 5. OBJECTIVES AND OUTCOME MEASURES

| **Objectives** | **Outcome Measures** |
| --- | --- |

| | |
|---|---|
| **Primary Objective**<br>To evaluate the impact of BCV on early mortality in EVD | Mortality at Day 14 after entry into the trial |
| **Secondary Objectives**<br>1) To evaluate the impact of BCV treatment of adults and children on:<br><br>a) Time to recovery<br><br>b) Late mortality<br><br>c) Viral load<br><br>d) EVD symptoms<br><br>e) EVD antibody response<br><br>2. To assess the safety of BCV treatment of adults and children | Secondary outcomes measures:<br>1. a) Time to meeting EVD treatment center discharge criteria<br><br>b) Mortality at Day 30 after first dose of study treatment<br><br>c) Day 4 viral load<br><br>d) Presence and duration of symptoms (Days 1-14)<br><br>e) Convalescent anti-Ebolavirus IgG titer (Day 30)<br><br>2. Incidence of SARs and key adverse events (Days 1-14) |

## 6. TRIAL DESIGN

This is an open-label, non-randomised, single arm trial, which will be conducted in Monrovia, Liberia. Collated retrospective data will be used to inform mortality targets for comparison of trial data. Patients with reverse transcription polymerase chain reaction (RT-PCR) confirmed Ebolavirus RNA (confirmed patients) are admitted to confirmed wards of the Ebola Treatment Centre. Confirmed patients will be screened for trial eligibility and invited to give informed consent to participate. Parents/guardians of children and representatives of severely ill patients will be invited to give consent on behalf of the patient where appropriate. Study dosing, sampling and data collection will begin after enrolment. **Table 1** shows the schedule of study procedures.

The primary objective of the RAPIDE clinical trial team is to assess a number of potential treatments for Ebolavirus disease and to triage them as (a) very effective, (b) promising and (c) showing little evidence of effectiveness. The RAPIDE strategy is therefore intended to find and assess a very effective treatment, if one exists, as quickly as possible, and in time to help the patients in the current outbreak. RAPIDE-BCV as described in this protocol, is the first single-arm triage study and will be followed by additional studies that will be designed based on the results of this first trial and the availability of investigational products. As such, RAPIDE-BCV is the first stage of a larger 'adaptive' study. Follow-on studies will be designed and submitted for ethical and regulatory approval as data and products become available.

*Table 1. Schedule of study procedures*

| Procedures | Screening | Enrolment | In-Patient visit per day | Day 4 (+/- 24 hours) | Discharge | Day 14 | Day 30 |
|---|---|---|---|---|---|---|---|
| Eligibility assessment | x | | | | | | |
| Pregnancy test | x (standard care) | | | | | | x |
| Informed consent | x | | | | | | |
| Demographics , co-existing morbidities, clinical history | | x | | | | | |
| Clinical evaluation including key adverse events | | x | x | | | x | x |
| SAR assessment | | | x | | | | |
| Record of other therapy | | x | x | | | | x |
| Administration of BCV* | | x | x x x (twice weekly) | | | x | |
| Routine blood draws** 2 x ≤4 mls EDTA, ≤4 mls SST, ≤2 mls FluOx | x | | | | x | | |
| Study blood draws*** Day 4: 2 x ≤4 mls EDTA, ≤4 mls SST, ≤2 mls FluOx Day 30: ≤4 mls EDTA, ≤4 mls SST | | | | x | | | x |

*Study treatment is planned for days 1, 4, 7, 11 and 14. Days may be altered if required (see section on dosing below). Patients who meet discharge criteria and leave the ETC before Day 14 will stop study treatment upon discharge from the ETC.

**Screening – Ebolavirus diagnostic PCR, serology, FBC, LFTs, glucose, urea and electrolytes, store residual. More than one diagnostic PCR may be done if required for standard care.

**Discharge – Ebolavirus PCR to confirm of viral clearance, serology, FBC, LFTs, glucose, urea and electrolytes, store residual. Pharmacokinetics will be analysed when possible. More than one confirmatory PCR may be done if required for standard care.

***Day 4 – Pharmacokinetics, Ebolavirus PCR, serology, FBC, LFTs, glucose, urea and electrolytes, store residual.

*** Day 30 – Serology and pregnancy test

The maximum amount of blood that will be obtained on any particular day for adult and pediatric patients is 14 mL. The total maximum amount of blood that will be obtained for research purposes over the full duration of the study is 21 mL for adult and pediatric patients. The amount of blood taken for standard care over the full duration of the study is approximately 35 mL. Adult and pediatric samples will be reduced in volume according to standard procedures such that no patient will have more than 0.6 mls/kg (>1%

blood volume) taken on any one day, nor more that 2.4 mls/kg (approximately 3% of blood volume) taken in any four week period. Standard care samples will be prioritized over research samples if volume reduction is required.

## 7. PARTICIPANT IDENTIFICATION

### 7.1. Trial Participants

Participants with laboratory confirmed EVD treated at a participating Ebola Treatment Centre and meeting the inclusion and exclusion criteria will be eligible to take part in the study.

### 7.2. Inclusion Criteria

- Male and female patients 2 months of age or older
- Laboratory confirmed infection with Ebolavirus infection.
- Women who are lactating must agree to stop breastfeeding (a standard recommendation for all EVD patients)
- Willingness to use barrier birth control for 3 months after recovery of EVD (a standard recommendation for all EVD patients)
- For patients 18 years and older: the ability to provide fully informed consent or the provision of consent from the representative of a patient deemed too unwell to comprehend what is being asked of them.
- For patients under 18 years: consent from a parent or guardian.

### 7.3. Exclusion Criteria

The participant may not enter the trial if the following applies:

- Any underlying disease or condition that, in the opinion of the principal investigator or treating physician, could jeopardize the participant's or healthcare workers safety or the participant's ability to comply with the protocol requirements.
- Patient unable to take oral IMP (e.g. severe vomiting unresponsive to anti-emetics, unconscious).
- Pregnancy.

## 8. TRIAL PROCEDURES

### 8.1. Screening

All patients with laboratory confirmed EVD will be considered for study participation. Treating staff will evaluate all patients for inclusion/exclusion criteria based on available clinical information. Pregnancy tests are done as a part of routine standard of care using the same blood taken for Ebolavirus diseases diagnosis. Women who are identified as pregnant will be informed by the treating staff and will not be enrolled to the study. Women who have a positive pregnancy test, but have aborted/miscarried or given birth within the previous month will not be excluded. Staff will not discuss informed consent with patients (or the parents/representatives of patients <18 years old) with underlying disease that prohibits study

inclusion or those unable to take oral IMP. The number of patients who meet these exclusion criteria will be anonymously recorded.

### 8.2. Informed Consent

Patients who have not been excluded based on the initial screening will be invited to discuss study participation. A patient may be enrolled in the study anytime within 24 hours of being informed of Ebolavirus positive PCR rsults. Therefore, if a patient has been excluded for a reason that resolves within this period, they may be approached upon resolution of the reason for exclusion. If a patient wishes to delay their decision regarding consent, they may enrol at any time within the 24 hour period.

Study participation will be discussed with adult patients who, by the judgement of the treating staff are sufficiently mentally and physically fit to provide such consent. Where the participant is deemed too unwell, the study will be discussed with a representative of the patient. In the case of children, the study will be discussed with a parent/guardian. For the purpose of consent, a child is defined as a patient less than 18 years of age. For the purpose of consent, an adult is a patient ≥18 years of age.

Written informed consent will be obtained from all patients or parents/guardians/representatives who agree to study participation. In the event of consent being given by the representative of a severely ill adult patient, the patient will be approached for consent if they gain the ability to discuss the study at a later time during their participation.

The number of patients who refuse to give informed consent will be recorded anonymously.

### 8.3. Assessments

All patients who meet inclusion and exclusion criteria will be enrolled. Study data will be collected on key symptoms, clinical characteristics and administration of all medications including BCV treatment. Diarrhoea and vomiting will be specifically monitored for changes in severity following administration of BCV as they are recognised dose-limiting side effect. These data will be collected daily for participating patients up to Day 14, or death, or discharge, whichever occurs first.

Discharge criteria are:

- 3 days without fever or significant symptoms AND
- A significant improvement in clinical condition AND
- Able to feed, wash and walk independently AND
- Ebolavirus RNA PCR negative.

The first day of study treatment administration is defined as Day 1. Patients who achieve discharge criteria before day 14 will stop study treatment upon discharge.

### 8.4. Follow Up Assessments (Day 14 and Day 30)

Survival at Day 14 is the primary end point of this trial, and defined as survival until past midnight from Day 13. Follow up assessments will be conducted on Days 14 and 30. Patients who have been discharged before either of these dates will be issued a mobile telephone for the purposes of follow-up. Patients will be invited to return to the treatment centre for these visits. When patients are not able or willing to attend a follow-up visit at the treatment centre, field workers will endeavour to visit patients in their home if

agreed by the patient. If a visit to the treatment centre and a field worker visit are both prohibitive (e.g. due to the patient living very far from the treatment centre), staff will follow-up with the patient by telephone.

Patient status on follow-up assessment days will be collected (admitted, met discharge criteria, deceased). A blood sample (≤5 mL) will be collected on Day 30 for assessment of convalescent anti-Ebolavirus IgG titer. Samples that cannot be collected on Day 30, but +/- 5 days will be accepted and analysed appropriately.

Primary/secondary outcome data for Day 14 and 30 can be collected at any point after the respective days. Any pregnancies which occur during the study period will be followed to obtain outcome.

## 8.5. Laboratory Assessments

Malaria immunochromatographic diagnostic tests will be performed on the triage blood sample as part of standard care. The results of these tests will be recorded.

For women of childbearing age (15-49 years) a chromatographic βHCG test will be performed on the triage and Day 30 blood samples. Urine testing is also acceptable.

Patients will have EDTA blood samples collected at triage for EVD diagnosis by PCR, prior to discharge to confirm clearance of viral RNA, and also for virus detection on Day 4 (+/- 24 hours). Anti-Ebolavirus IgG will be evaluated at each of these study days and additionally on Day 30. The triage and discharge samples are a part of standard care and may be repeated if required. The Day 4 and Day 30 samples are for study purposes only. The PCR result for all samples, including the day 4 sample will be shared with the clinical team in real time. Residual volumes of all samples will be stored for study analysis. The triage sample will have been obtained as a part of standard care, before the patient has provided informed consent; residual volume from this sample will be used for study purposes and the results of the PCR and other blood tests will be recorded.

EDTA samples obtained for PCR are typically ≥4 ml whole blood in an EDTA tube; finger or heal-pricks of blood on a dry swab are sometimes obtained when venepuncture is not possible. Ebolavirus RNA will be detected by PCR, as per local clinical laboratory protocols, for the triage, day 4 and discharge samples. Additionally, plasma anti-Ebolavirus antibody titres will be determined by analysis of residual volumes by partner laboratories. This is to determine whether the treatment influences the subsequent production of specific antibodies.

Standard care for EVD patients at the study site includes a screening full blood count (FBC – ≥4 mls EDTA), liver function tests, urea and electrolytes (LFT, U+Es - ≥4 ml serum separator tube) and glucose (≥2 ml potassium oxalate/sodium fluoride tube). These tests will be repeated on Day 4 and at discharge. Pharmacokinetic analysis will also be performed on the Day 4 and Discharge potassium oxalate/sodium fluoride sample.

Aliquots of residual plasma from the same samples will be stored at -20°C or colder and subsequently shipped to international partner laboratories for confirmatory testing and quantification. If volume remains, additional testing relevant to the pathogenesis of EVD or effects of the study treatment will be performed. Residual cells or plasma supernatant may be retained for host genetic studies to identify susceptibility and severity markers.

## 8.6. Discontinuation of Trial Treatment

Each participant has the right to discontinue use of study treatment at any time. In addition, the Investigator may discontinue treatment at any time if the Investigator considers it necessary for any reason including:

- Ineligibility (either arising during the trial or retrospectively having been overlooked at screening)
- An adverse event which requires discontinuation of the trial medication
- Disease progression which requires discontinuation of the trial medication
- Patient or physician decision

The reason for any discontinuation of treatment will be recorded in the case record form.

## 8.7. Withdrawal from the Study

Patients, or parents/guardians/representatives when they have given consent on behalf of the patient, are free to withdraw consent and stop study participation at any time for any reason. In this case, the patient's clinical management will continue to be provided according to standard care and will not be affected by the decision to withdraw. Outcome data and the reason for withdrawal will be recorded when possible. Data collected up to the date of withdrawal will be used in study analysis.

# 9. Trial Therapy

## 9.1. Investigational Medicinal Product

BCV has been provided to more than 400 patients with serious or life-threatening infections with dsDNA viral infections and no alternative treatment options under expanded access or emergency investigational new drug or local equivalent regulations in nine countries, including the United States, Canada, France, United Kingdom, Spain, Switzerland, Austria, Israel, and Peru. The safety database of over 1,000 individuals exposed to BCV has shown no evidence of hematologic toxicity or nephrotoxicity. BCV is progressing in two clinical development programs: one for the prevention of cytomegalovirus infection in hematopoietic cell transplant recipients, and the other for the treatment of adenovirus infection. Phase 3 studies (CMX001-301 or SUPPRESS Trial for CMV; CMX001-304 or AdVise Trial for adenovirus) are being conducted in the United States, Canada, and Belgium.

BCV is supplied as a 100 mg tablet for oral administration.

100 mg tablet: BCV tablets are formulated as dry-blend, direct compressed tablets containing 100 mg of BCV as free acid. In addition to the active ingredient, BCV tablets contain microcrystalline cellulose, mannitol, crospovidone, and magnesium stearate. Tablets have a re-test period of 2 years.

In the case of patients unable to swallow pills, the tablets will be dissolved in water. When possible, treatment should be taken with a meal.

## 9.2. Dosing

Patients weighing ≥50 kg will be administered a 200 mg loading dose (two 100 mg tablets) on Day 1, followed by 100 mg on Days 4, 7, 11 and 14 (notwithstanding adjustments as detailed below).

Patients weighing <50 kg will be administered a 4mg/kg initial dose on Day 1, then 2 mg/kg on Days 4, 7, 11 and 14 (notwithstanding adjustments as detailed below).

In the case of a dose not taken on the assigned study day, it should be taken as soon as possible. The following dose should be taken 3 days after the administration of the missed dose, and treatment continued every 3 days until completion of 5 doses.

Patients who cannot swallow tablets will receive tablet(s) dissolved in water. Tablets will be dissolved in 20 mL of water, which can be divided to give doses smaller than 100 mg according to weight (see dosing table in Appendix 3), and administered immediately. When possible, drug should be administered with a low fat meal. Full details of treatment preparation and administration will be outlined in the study procedures.

BCV will be administered under the supervision of clinical staff. Patients who vomit within 30 minutes of receiving treatment will receive an additional dose as soon as they are able. A maximum of 1 redose will be given for any individual dose. In the event of a redose being vomited within 30 minutes of administration, patients will receive the next scheduled dose on the day after the vomited dose. Remaining dose(s) will be taken every three days until completion of 5 doses. No dose adjustments will be made to account for possible effect of gastrointestinal symptoms on absorption. All administration of study drug will be recorded in the CRF.

### 9.3. Treatment Interruption

In the event of a SUSAR or other significant treatment related event, study investigators will determine if study treatment will continue on the basis of clinical picture and severity and resolution of the event. Consultation with the Independent Data Monitoring Committee will be made in the case of uncertainty to determine if the treatment should be discontinued.

### 9.4. Storage and Accountability

Study drug should be stored at controlled room temperature between 15 and 25°C, with excursions permitted up to 30°C. Inventory, dispensing and accountability of study treatment will be tightly controlled. Treatment dispensed to the Confirmed ward, but not consumed by patients will not be returned due to infection control procedures and will be destroyed. Full accountability procedures and logs will be detailed in the corresponding study reference manual.

### 9.5. Supportive Therapy

The supportive therapy received by patients will not be affected by participation, or non-participation, in the trial. Trial participants will all receive standard supportive therapy and this will be recorded.

## 10. SAFETY REPORTING

It will not be possible to fully determine the safety profile of the study drug within this trial design because the sample size is insufficient. Due to the nature of the symptoms of EVD (see **Table 1**) [2] and the relatively small sample size it will be very difficult to differentiate between symptoms of the disease and events due to treatment, and the trial will have limited power to do this. Our investigation of safety will therefore focus on Serious Adverse Reactions (SARs) and Suspected Unexpected Serious Adverse Reaction (SUSARs) and key adverse events.

### 10.1. Definitions for safety reporting

**Serious Adverse Reaction (SAR)** – A serious untoward and unintended response in a participant to the study treatment, which is related (or has a reasonable possibility of being related) to any dose administered to that participant.

To qualify as "serious" the response must meet one of the following criteria:

- results in death
- is life-threatening
- requires inpatient hospitalisation or prolongation of existing hospitalisation
- results in persistent or significant disability/incapacity
- consists of a congenital anomaly or birth defect.

Other 'important medical events' may also be considered serious if they jeopardise the participant or require an intervention to prevent one of the above consequences.

**Suspected Unexpected Serious Adverse Reaction (SUSAR) -** A serious adverse reaction, the nature and severity of which is not consistent with the information about the medicinal product in question set out in the investigator's brochure (IB).

### 10.2. Procedures for safety reporting

All SARs will be reported by the site to the operational team within 48 hours. In discussion with the site staff, the operational team will assess the SAR for expectedness and relatedness and report all SUSARs to the independent data monitoring committee (IDMC) immediately. The operational team will also report any SUSARs to all the relevant parties as required within 7 days.

Evaluation of expectedness and relatedness of reactions will consider the opinion of the site staff and the expected signs, symptoms and events associated with Ebolavirus disease or BCV treatment according to the Investigators' Brochure. Evaluation of expectedness will be made with reference to the Investigator's brochure.

**Table 1. Symptoms of Ebolavirus disease (WHO Ebola Response Team, 2014[2]; ISARIC WHO Viral Haemorrhagic Fever CRF)**

| Known symptoms and clinical events of EVD: | |
|---|---|
| Death | Coma or unconsciousness |
| Fever | Unexplained bleeding |
| Fatigue | Hematemesis |
| Loss of appetite | Blood in stool |
| Vomiting | Bleeding gums |
| Diarrhoea | Bloody nose |
| Headache | Bloody cough |
| Abdominal pain | Other bleeding |
| Joint pain | Bleeding at injection site |
| Muscle pain | Blood from vagina |
| Chest pain | Blood in urine |
| Cough | Bleeding under the skin/bruising |
| Difficulty breathing | Back pain |

| | |
|---|---|
| Difficulty swallowing | Decreased urine output |
| Conjunctivitis | Lower chest wall indrawing |
| Sore throat | Hearing impairment |
| Confusion | Tinnitus |
| Hiccups | Seizures |
| Jaundice | Hepatomegaly |
| Eye pain | Splenomegaly |
| Rash | Lymphadenopathy |

Diarrhoea (and, less frequently, nausea, vomiting, anorexia, loss of appetite, hypoalbuminemia, and abdominal pain) and hepatobiliary events (including elevation of serum aminotransferases) are considered possible side effects associated with the use of BCV. These side effects overlap with some EVD disease manifestations as highlighted above. For diarrhoea and vomiting, severity and temporal association with study drug will be included in regular IDMC reports. Patients will be re-dosed if vomiting within 30 minutes of receiving treatment; otherwise no dose adjustments will be made to account for possible effect of gastrointestinal symptoms on absorption.

## 11. STATISTICS

### 11.1. Description of Statistical Methods

The trial is designed to distinguish between three situations: (a) the treatment is very effective, (b) the treatment is promising and (c) the treatment is ineffective. The effectiveness of the treatment will be judged in terms of the probability that a treated adult patient will survive to Day 14, denoted by p. Note that p represents the true value of the probability rather than any estimate that might be found from the trial data. Three possible values of p are used for guidance. If $p = 0.800$ then the treatment will be regarded as very effective. If $p = 0.667$ then the treatment will be regarded as promising. If $p = 0.500$ then the treatment will be regarded as ineffective.

Both children and adults will be treated in this trial. However, they will be analysed separately as the effects of treatment may be different in the two groups. The primary analysis will concern the results from adults, and a secondary analysis of the data in children will be used to ascertain whether the conclusions from those adult patients should apply to children. The stopping rules governing the study will be based on data from adults only, and once met the whole study, in adults and children, will be stopped. In this section, sample sizes mentioned refer to the number of adult patients in the trial, and analyses refer to analyses of the data from adult patients only. For the purpose of analysis adults are defined as all patients ≥50kg in weight and/or ≥18 years of age.

The choice of guide values for p has been made following an analysis of individual level data on patients from the Gueckedou treatment centre. Data are available on the outcomes of 778 patients with laboratory confirmed Ebolavirus infection. Of these, 239 survived. In the 140 patients with ages available and less than 16 years (children) 27.1% survived, while in the other 638 patients (adults), 31.5% survived. This is a survival rate of 30.7%, which is consistent with rates reported elsewhere. Following calculation of this initial estimate, the sample was reduced to reflect the type of patient who would feature in the primary analysis of the trial. Only adult patients who were treated in the hospital itself, and had known dates of

onset of symptoms and of death or discharge from hospital were included. This led to a sample size of 500 (all suitable patients were included, that the sample size is exactly 500 is coincidental). Failure was defined as dying on or before the 14th day after onset of symptoms, and success was taken to be lack of failure. Thus, patients dying after Day 14 were treated as successes, as will occur in the trial analysis. Of these 500 patients, 237 succeeded. This is a success rate of 47.0%. The probability of success for an untreated control can thus be estimated as 0.470, with a 95% confidence interval of (0.426, 0.514). The success rate was reasonably constant over the 8 months of operation of the Gueckedou treatment centre and did not appear to depend on the number of days between onset of symptoms and hospitalisation.

As a result of this analysis, p = 0.500 has been set as the success rate for which the experimental treatment would be considered ineffective.

### 11.1. The Level of Statistical Significance

The design specification is presented in **Error! Reference source not found.**For each of the three guide values of p, a high probability is set for reaching the most appropriate conclusion.

### 11.2. The Number of Participants

The maximum number of adult participants required to evaluate the efficacy is 140. The data will be analysed sequentially and stopping rules applied to each investigational product. It is most likely that the number of participants will be fewer than 100 (see 11.3).

### 11.3. Criteria for the Termination of the Trial

Research sites will inform the data management centre every time that an eligible patient is enrolled into the trial. Fourteen days later, they will report to the data management centre whether that patient did, or did not, survive to Day 14. Every time that a Day 14 report is received on an adult patient, a point will be plotted on Figure 2. This is plot of the number of (adult) survivals reported so far against the number of (adult) Day 14 reports received. The plot is compared with one of the three boundaries. As soon as the plot reaches one of the boundaries, the trial will be stopped. Conclusion (a), (b) or (c) will be drawn according to whether the green, orange or red boundary is reached. The trial would stop with conclusion (a) if all of the first 24 patients recruited survived to Day 14, and with conclusion (c) if none of the first 12 patients recruited survived to Day 14. The stopping boundaries are truncated at 140: this is the maximum sample size.

**Figure 2.** Stopping rules for the trial

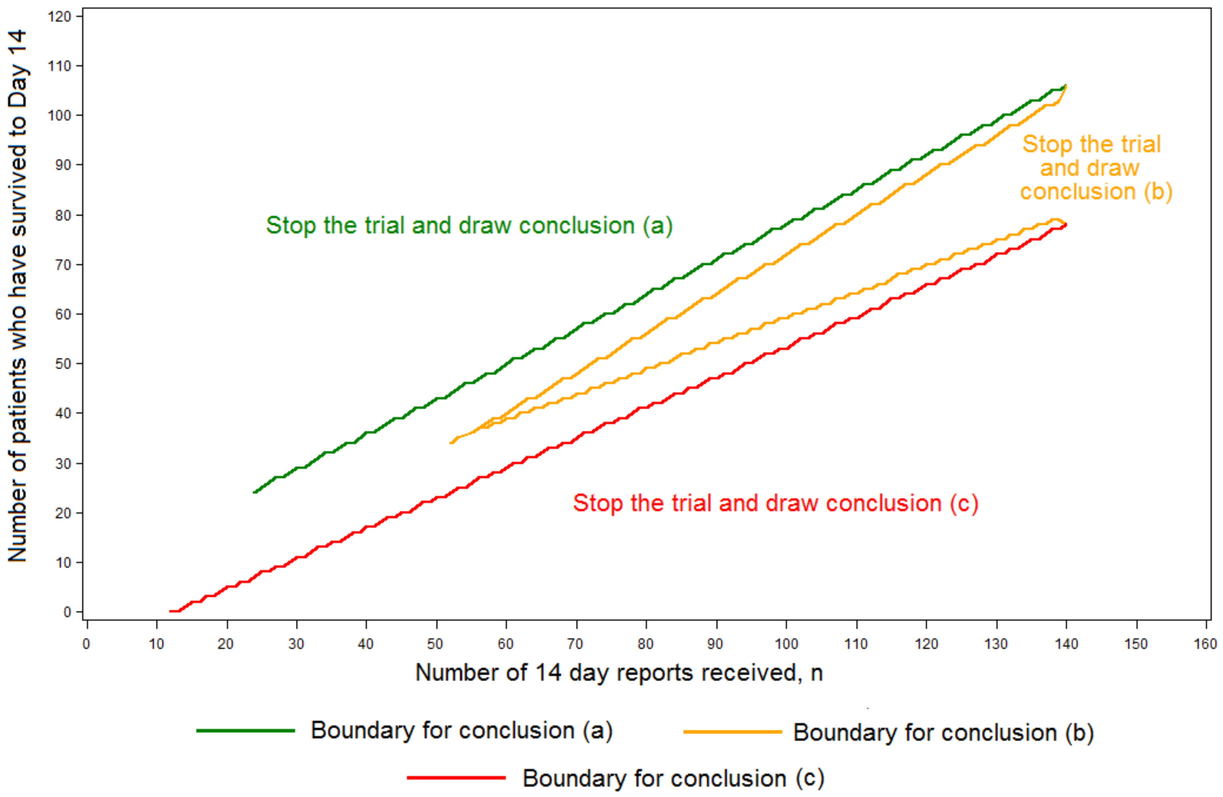


The properties of the design have been calculated exactly, based on the independent Bernoulli distributions of each patient outcome. These are shown in **Table 3**. The specified probability of reaching the most appropriate conclusion when p = 0.500, 0.667 or 0.800 is exceeded in each case (it is not possible to match them exactly due to the discrete nature of the data). It can be seen from **Table 3** that the average (were many such trials to be repeated) and the median sample sizes are much less than the maximum sample size of 140, and even the $90^{th}$ percentiles of sample size (the values before which the trial will be stopped with probability 90%) do not reach 140.

**Table 3**: Exact properties of the trial design

| p: true probability of surviving for 14 days | probability of concluding: (a) | (b) | (c) | final sample size: average | median | $90^{th}$ %ile |
|---|---|---|---|---|---|---|
| 0.414 | 0.000 | 0.002 | 0.998 | 37.7 | 35 | 58 |
| 0.500 | 0.000 | 0.100 | 0.900 | 60.5 | 56 | 97 |
| 0.667 | 0.034 | 0.950 | 0.016 | 70.0 | 65 | 96 |
| 0.800 | 0.908 | 0.092 | 0.000 | 68.7 | 65 | 105 |
| 0.850 | 0.997 | 0.003 | 0.000 | 49.4 | 45 | 72 |

A second plot will be maintained of the number of patients who have been recruited minus the number known so far to have died within 14 days (S*), against the number recruited. When records are finally completed for all of these patients, the number who have survived 14 days must be less than S*. The plot of S* will be compared with the lower boundary of Figure 1 and the trial will be stopped with conclusion (c) if it is crossed. Once the records are complete to 14 days, the main plot must also lie below the lower

boundary: there is no uncertainty in this prediction. This safety plot would ensure a rapid reaction to a treatment that proves to be lethal in the short term.

### 11.4. Final Analysis

When the trial has been completed, a point estimate and a 95% confidence interval will be computed for p using the method of Jovic and Whitehead,[17] which allows for the sequential nature of the trial.

### 11.5. Inclusion in Analysis

It is important to note that the primary sequential analysis described uses only data from adult patients who were recruited more than 14 days previously. Although it may be known that some more recently recruited patients have already died, their data will not yet be used, to avoid bias due to information on death being available sooner than information on survival.

### 11.6 Analysis of the data on children

The stopping rule described in Section 11.3 will be applied to data from adult patients only. When the trial is concluded for adult patients, the recruitment of children will also be terminated. The success rate for children will be estimated, and a 95% confidence interval will be computed using the exact method of Clopper and Pearson.[18] The difference between the success rates of children and adults will be analysed using approximate methods based on the normal distribution. Secondary endpoints will be analysed separately for both children and adults. For the purpose of analysis children are defined as all patients who are both <50kg in weight and <18 years of age.

### 11.7 Procedure for Accounting for Missing, Unused, and Spurious Data.

While considerable effort will be made to ensure all patients are followed up at Day 14 and Day 30 (through return visits to clinic, phone, email or field-researcher visits), some patients may not be contactable. Leeway will have to be allowed for late reporting, but this should be limited: it may be that data on survival to Day 14 is not entered until Day 16 in order to avoid remaining bias due to late reporting of survival. However, if it turns out that a patient died on Day 15 or later, *then they are survivors* for the purpose of this study. If they walked away on Day 7, seemingly cured, it might be assumed that they survived to Day 14. Some judgment will necessarily be used here.

### 11.8 Rationale for statistical design choice

In the context of the current EVD outbreak, a simple, robust and rapid trial is essential. Mortality dominates all other patient endpoints. Most deaths occur within 14 days of diagnosis, and so survival (or not) to Day 14 represents the most relevant clinical endpoint. The situation at Ebola Treatment Centres is highly challenging, and so this study places minimal additional responsibilities on them.

We believe that if evidence of a survival rate exceeding 0.800 there is strong evidence that the treatment is effective. Evidence that the rate lies close to 0.667 would identify the treatment as being worthy of further study. Although the patients are likely to be a heterogeneous group, there is little opportunity to use stratification or covariate adjustment in this trial. That is because the target values of the probability

of survival to 14 days, 0.500, 0.667 and 0.800, are assumed to be relevant to everyone. For stratification, different targets would be needed for each stratum.

A fully sequential design is proposed so that the conclusion of the trial can be made available as quickly as possible. If all treated [adult] patients die before Day 14, then only 12 patients will be required, and if all survive, then 24 will be sufficient. Rapid reporting of minimal information would appear to be feasible, and in keeping with the urgency of the situation. Until the trial is terminated, data managers need only compare the number of survivals observed so far with the stopping boundaries shown on Figure 2. A table of values will be provided for this purpose.

The approach described is related to the sequential medical plans of Bross[19] but his designs where centred on p = 0.50, rather than p = 0.67 as required here. It is also a form of double triangular test, as described by Whitehead and Whitehead and Todd.[20, 21] The latter paper summarises some implementations of the method. The analysis is based on an orderings approach introduced by Fairbanks and Madsen.[22] Exact calculations have been made for this particular design, using SAS, and this package will be used for the analysis.

## 12. DATA MANAGEMENT

### 12.1. Data Capture

Patient files will serve as source clinical and drug dosing data for the study. Due to infection control procedures, paper documents cannot be taken outside of the high-risk containment zone; therefore records may be dictated to a secondary document outside of the zone and the primary document incinerated. Where possible, electronic data entry will capture source data directly or source documents will be photographed and archived. Original data files from the site and research laboratories will provide source data for laboratory testing results. Data recorded by Medecins Sans Frontieres as a part of the standard case investigation form will be used to support the classification of disease severity as well as for validation and comparison of study data.

Data will be entered to a central study database on MACRO software. Data management, reporting and storage within this trial will comply with the requirements of European Union data protection laws, ICH Good Clinical Practice and FDA 21 CFR Part 11.

### 12.2. Access to Data

Data sharing will be under the aegis of the Trial Steering Committee and will adhere to the principles of rapid, open access as outlined in the World Health Organisation report on Ethical considerations for use of unregistered Interventions for Ebolavirus disease.[23]

## 13. QUALITY ASSURANCE PROCEDURES

Governance of the trial will be through the Trial Steering Committee (TSC), the Trial Operation Group (TOG) and the Independent Data Monitoring Committee (IDMC).

- The TSC will be the primary decision making body, with membership from each partner institution, a senior representative from each participating country, and other independent experts. Members

of the TSC are given in Section 1 (members may change if necessary). Decisions of the Committee will be by consensus. WHO will be part of the Committee on an ex-officio basis. Terms of reference for the TSC will set out the full details of the membership, operations and remit.

- The TOG will be led from the ISARIC Coordination Centre in Oxford and report to the TSC. This team will provide operational oversight and day-to-day management of the trial to ensure compliance to follow up and critical factors such a retention and data quality. This team will comprise those with responsibility for the operational activities and so include representation from each site, data management, trial operations, training and logistical support, laboratory sample processing and handling.
- The IDMC will be responsible for reviewing the data from the study. Membership will include individuals with statistical, clinical and trial expertise. An IDMC charter will fully set out the committee's remit, membership and full definition of their operational conduct in terms of reviewing efficacy and safety data and reporting their recommendations to the TSC.

The trial will be conducted in accordance with the current approved protocol, relevant regulations and standard operating procedures. Regular monitoring will be performed to ensure quality control. Data will be evaluated for compliance with the protocol and accuracy. Following written standard operating procedures, the monitors will verify that the clinical trial is conducted and data are generated, documented and reported in compliance with the protocol and the applicable regulatory requirements.

The MACRO software will be configured to automatically cross-validate across entire patient records according to the specific requirements. Data Clarification requests will be built in to the system. The data validation and quality processes will comply with the new FDA and EU recommendations for data level trial monitoring and therefore allow for data quality to be assured in real time as a mechanism for on site monitoring.

## 14. ETHICAL AND REGULATORY CONSIDERATIONS

### 14.1. Regulations, Guidelines and Approval

The Investigators will ensure that this study is conducted in compliance with the current revision of the Declaration of Helsinki and the applicable principles of International Committee on Harmonization Good Clinical Practice Guidelines. Regulatory approval to conduct the study and import investigational products will be obtained from national authorities. The protocol, informed consent form and investigators brochure will be submitted to the National Research Ethics Committee and regulatory authorities in-country, as well as the Medecins Sans Frontieres Research Ethics Committee, the World Health Organisation Research Ethics Committee, and the Oxford Tropical Research Ethics Committee for written approval.

The Investigator will submit and, where necessary, obtain approval from the above parties for all substantial amendments to the original approved documents.

### 14.2. Considerations in Informed Consent

**STANDARD CARE UPON PRESENTATION:** Patients are triaged to Suspected and Probable wards upon presentation based on symptom and contact history. Blood is drawn for Ebolavirus PCR testing daily according to standard care. Results are reported back to the clinical staff, who then visit patients to discuss the results. When laboratory results positive for Ebolavirus are received from the laboratory, a senior medical staff and a social worker visit the patient to discuss the results. Clinical and social counseling are provided as the patient is moved to the Confirmed ward.

**PROCESS FOR TAKING INFORMED CONSENT:** Written informed consent to participate will be required from all participants or their representative. The staff who undertake the discussion of test results will decide when the patient should be approached regarding the study. This discussion should occur as soon as possible after the availability of confirmed results, but at a time when the patient is emotionally stable and the staff feel that they are capable of considering the risks and benefits of participation.

All eligible patients should be offered the opportunity to participate in the study. When the clinical staff and/or social workers agree that an adult patient is mentally and physically fit to discuss and consider participation in the study, the patient will be approached. If an eligible patient is not deemed fit to consent, a representative will be approached for consent if available. Appropriate representatives will be selected according to the standard Ebola Treatment Centre processes for determining who can make decisions on a patient's behalf. In the case that the patient is unfit to consent and there are no appropriate representatives available the patient will not be invited to participate.

Consent will be requested by the local study staff or another suitable qualified and trained person authorised to do so by the Principal Investigator.

**ADULTS:** Adult patients for the purpose of consent are defined as ≥18 years of age. Adults who are deemed fit to discuss and consider participation by the medical and social staff will give consent independently. If an adult is not considered mentally and physically fit to give informed consent, consent will be sought from a close relative who attends the clinic. When consent is obtained from a relative, the patient will be approached for consent if at any time during study participation their condition improves such that they can consider consent.

**CHILDREN:** Children are patients less than 18 years of age. Consent for children to participate in the study will be sought from the parents or guardians of the child. Study participation will be discussed at the discretion of the clinical and social staff with children of suitable physical and mental fitness as well as emotional maturity.

**DISCUSSING CONSENT:** Participants or their representative will be presented with an informed consent form that explains the purpose, methods, risks and benefits of the study. The form will be available in relevant written local languages. If the patient or representative cannot read, the form will be read to them in the presence of a witness when available. If a consent form is not available in a language familiar to the participant, staff will verbally translate the form into the language of the patient. Standardization of translation will be supported by the use of recorded verbal translations with appropriate language and explanations in local terms that will be used to train study staff. When possible, a second staff will be present during the informed consent discussion to ensure that translations are appropriate, the form is read accurately, that all of the patient's questions have been addressed and to act as witness when possible. This will be facilitated by the paired working system of the clinics, but may not always be possible due to human resource limitations and limited shift times.

**DOCUMENTING CONSENT:** Patients or their representative will be asked to sign and date an informed consent form prior to participation. Those unable to write will be asked to make a mark on the signature

line only. If a witness was present during the consent process s/he will co-sign the consent form to confirm the accurate reading of the form and the consent of the participant/parent/guardian/representative. If consent for an adult patient is given by a representative, patients will be asked to sign a separate informed consent form when/if they are deemed physically and mentally fit at a later point during the study. When a second staff is present for the informed consent discussion, s/he will sign the informed consent form. A child's assent to participate will not be recorded.

**ARCHIVING OF INFORMED CONSENT FORMS:** Due to infection control procedures, paper cannot be transferred externally from the confirmed wards. Therefore informed consent forms will be photographed and archived electronically, then destroyed according to waste disposal procedures for contaminated substances. Patients will not be given a copy of the signed form as it is not permitted to leave the high-risk containment zone. Patients or their representatives will be offered an unsigned copy of the form to retain upon discharge.

### 14.3. Participant Confidentiality

The trial staff will ensure that the participants' anonymity is maintained. All study documents and samples will be labeled with an anonymous study code. Identifying information collected as a part of this study will remain confidential. For the purpose of ensuring data integrity and to facilitate quality assurance, study records will link to clinic files, which include patient's name and other identifying information. Participants' names will be recorded at the time of enrolment to allow for their identification at follow-up visits. However identifiable information will be linked to stored data or samples only by a protected Master List. This list will not be shared outside of the site study staff and no identifying information will be transferred between sites. All CRFs and samples will be labeled with a study identification number only and stored in suitable secure locations. Only persons who have signed the locally appropriate data protection training will have access to the password-protected computer where entered data is stored. After conclusion of the project, data will be removed from the computers and stored in a safe place. Any scientific publications or reports will not identify any patient by name or initials. When the research team reviews their notes, they are also bound by professional confidentiality. All study data will be stored in secure databases only accessible to study staff. Study sponsors and health authorities will be given controlled access for the purpose of audit when necessary.

### 14.4. Sample Management

Aliquots of EDTA blood-derived plasma samples will be stored initially at the site clinical diagnostic laboratory. Subsequently, aliquots will be shipped to national and/or international partner laboratories according to national and international regulations. Samples will be labeled with a unique study identifier prior to shipping and any patient identifiable data will have been removed. The Country Principal Investigator and Chief Investigator will have access to the enrolment log linking study identifiers with patient identifiers. Material transfer agreements and export/import licenses (where required) will be obtained, and international regulations on storage and shipping of hazardous samples will be followed. Sample custody will be maintained by the investigators and decisions regarding use and transfer of samples will be made by the TSC. Research samples will be stored indefinitely and approval from the sponsor and/or ethics committees, as appropriate, will be sought prior to destruction. The study investigators are responsible for biological deactivation and ensuring appropriate destruction of any residual materials.

## 14.5. Risks and Benefits

There are no approved treatments for EVD and the standard of care remains supportive care and treatment of complications. An analysis of data on 3343 confirmed and 667 probable EVD cases estimates a case fatality rate of 70.8% (95% confidence interval [CI], 69 to 73).[2] Unpublished data from MSF indicates that the survival rate in laboratory confirmed cased of Ebolavirus infection admitted to Gueckedou Ebola Treatment Centre is not likely to be more than 50% overall (see statistics section). BCV has demonstrated antiviral activity against EVD in vitro in independent testing performed by the Centre for Disease Control and National Institutes of Health; in vitro effects were demonstrated at concentrations that encompass the range of EC50 values observed for dsDNA viruses which have been effectively treated with BCV in humans and animal models, and which have been achieved in patients treated for dsDNA virus infection. For these reasons, the proposed study is considered a reasonable first step with regards to determining whether BCV has a potential benefit in patients with laboratory confirmed Ebolavirus infection.

BCV therapy has a number of potential risks.

BCV therapy has the potential for GI side effects, specifically diarrhea, however it is possible that the GI side effects of BCV therapy will overlap with the GI effects of EVD. Alternatively, patients with severe EBOV diarrhea may not absorb BCV as well as those without diarrhea. However, in patients with adenovirus enteritis with severe diarrhea after HCT, the treatment of adenovirus with BCV tended to stabilize or improve symptoms, rather than worsen them. In addition to surveillance for and reporting of all SUSARS, subjects enrolled in this study will be monitored specifically for GI signs and symptoms, especially diarrhea, as experience from Study CMX001-201 indicates that these events are likely associated with the administration of BCV after three or more weeks of therapy.

As described in the IB, BCV is considered a potential human carcinogen based on the findings from a 13-week toxicology study in rats, in which specific tumors (mainly adenocarcinomas) were observed in BCV-treated animals much earlier than would be expected over the course of a normal lifespan. However, any carcinogenic risk associated with administration of BCV in this patient population is considered to be outweighed by the potential benefit of preventing the significant mortality associated with EVD.

As described in the IB, in embryo/fetal development studies, decreased fetal body weight and survival (increased resorptions) as well as morphological changes (external malformations and visceral and skeletal developmental variations) were associated with BCV therapy at doses of 1.5 and 4.5 mg/kg/day. However, to date the fatality in foetus' of pregnant women with EVD has been 100% and therefore any feotal risk associated with administration of BCV in pregnant women is likely to be outweighed by the potential benefit of preventing the significant foetal mortality associated with EVD in pregnancy. However, given the current lack of data on the efficacy of BCV treatment in humans infected with Ebolavirus, the theoretical risk of embryotoxicity precludes the administration of BCV to pregnant women.

The risks of conducting this trial include the possibility that the operation of the Ebola Treatment Centre (ETC) where the trial is conducted is compromised by any of the following events: a.) the ETC is overwhelmed by suspected EVD cases referred or self-referring in the belief that an effective treatment is available at the ETC, b.) that the ETC security is breached by persons seeking access to the investigation product, c.) that the security and safety of the ETC and ETC staff is compromised if there is a perception that the investigational product or the clinical trial are harmful to individual or community interests.

Whilst these are significant risks they are not in themselves arguments for not conducting this trial, since these risks will exist for all ETCs conducted in this setting, although the risks may be most acute for the earliest trials conducted.

Mitigation of these risks will be achieved by the following activities: A) Community sensitization and participation in the run up to trial initiation. Experienced MSF health promotion and community outreach workers will conduct this activity. B) Study drug will stored off site in a locked metal cabinet within a secure

building. C) Sufficient study drug for the current number of enrolled patients will be transported to the ETC daily. D) Contingency plans for handling an increase in the number of suspected cases will be developed, including strict criteria for closing to new admissions at the site conducting the study and the identification of satellite ETC centers to accept patients that cannot be acceptable at the ETC conducting the trial. E) evacuation plans will be in place in the event of a major security breach.

### 14.6. Expenses

Clinic and treatment costs are covered by Medecins Sans Frontieres (and/or other organization/institution funding the site, including partner laboratories) for all patients presenting to the Ebola Treatment Centre regardless of study participation. The study drug will be supplied without cost to the patient. Patients will be issued a mobile phone for the purposes of follow-up. This phone and SIM card and SIM card will become the property of the patient. Reasonable travel expenses and compensation for loss of earnings will be paid for attending the Day 14 and Day 30 follow-up at the Ebola Treatment Centre. No other funding or incentives for participation will be given to the patients.

### 14.7. Contemporaneous Studies

Patients cannot enroll to any other clinical trial that involves a therapeutic or care intervention while they are participating in this study. If participating sites are undertaking observational studies, patients may be co-enrolled provided the combined study procedures are considered safe and appropriate for the patients and there is no conflict of outcomes or endpoints between the studies.

### 14.8. Alternatives to Study Participation

All patients will be treated with the best available care regardless of study participation. Patients are free to decline participation in this study without affect on the standard care provided.

A supply of BCV will be provided for compassionate use in patients not enrolled to this study, at the discretion of the treating doctor.

### 14.9. Community Engagement

Medecins Sans Frontieres (MSF) runs comprehensive Health Promotion activities alongside all Ebola Treatment Centres. Standard interventions include dissemination of health promotion messages to health authorities and communities regarding MSF activities and information on EVD transmission, prevention, reporting and response. Information on this research study would be added to these messages.

Messages to the community are disseminated by networks of MSF Health Promotion workers who engage with local leaders that serve as primary sources of information for the communities. Radio broadcasting and printed information sheets and posters are also common tools. This standard protocol on dissemination of critical messages would be used to inform the communities about research done at the Ebola Treatment Centre. Information regarding the nature and purpose of the research would be distributed with a variety of tools across these networks.

Messages to health authorities are disseminated to District and County Health Officers by the MSF Medical Team Leader and Field Coordinator. Education on the current development status of EVD treatments,

clinical research studies ongoing in West Africa and the scientific background and methods of the current project would be distributed to and discussed with the health authorities already engaged as a part of the Health Promotion activities.

MSF also provides psychological and social support to EVD patients. When presenting to the Ebola Treatment Centre, patients and families will be approached by a team social worker to facilitate the psychological process throughout admission, EVD confirmation, treatment and when required, death and bereavement. Patients who are discharged from the Treatment Centre will be supported in their return to the community. This team will be engaged in the research study according to the requirements of the sites to assist with explaining the nature and methods of the research, obtain informed consent, and discuss the study with the family.

### 14.10. Drug Access Post-Study

The study investigators and partners are in discussions with the producers of BCV to provide access to BCV upon completion of the study if it is demonstrated to be effective.

## 15. FINANCE AND INSURANCE

### 15.1. Funding

The trial is funded by the Wellcome Trust.

### 15.2. Insurance

The University of Oxford is arranging appropriate insurances to provide for the University's responsibilities, as Sponsor, to research subjects; and, to cover the legal liabilities of the University to those engaged by the University in the performance of this research. The University will also arrange, or arrange in conjunction with other participating partners those emergency medical repatriation facilities which can be achieved, subject to the exigencies of arranging such at the material time.

## 16. PUBLICATION POLICY

Results from the trial will be published in open access and the data will be available for sharing.

## 17. REFERENCES

1. WHO. Ebola Response Roadmap Situation Report 129 August 2014: Available from: http://www.who.int/csr/disease/ebola/situation-reports/29-august-2014.pdf?ua=1.
2. WHO Ebola Response Team. Ebola virus disease in West Africa--the first 9 months of the epidemic and forward projections. N Engl J Med. 2014;371:1481-95.
3. Kucharski AJ, Edmunds WJ. Case fatality rate for Ebola virus disease in west Africa. Lancet. 2014;384:1260.
4. Gire SK, Goba A, Andersen KG, Sealfon RS, Park DJ, Kanneh L, et al. Genomic surveillance elucidates Ebola virus origin and transmission during the 2014 outbreak. Science. 2014;345:1369-72.
5. Fowler RA, Fletcher T, Fischer WA, 2nd, Lamontagne F, Jacob S, Brett-Major D, et al. Caring for critically ill patients with ebola virus disease. Perspectives from west Africa. Am J Respir Crit Care Med. 2014;190:733-7.

6. Mahanty S, Bray M. Pathogenesis of filoviral haemorrhagic fevers. Lancet Infect Dis. 2004;4:487-98.
7. Takada A, Kawaoka Y. The pathogenesis of Ebola hemorrhagic fever. Trends Microbiol. 2001;9:506-11.
8. Bausch DG, Towner JS, Dowell SF, Kaducu F, Lukwiya M, Sanchez A, et al. Assessment of the risk of Ebola virus transmission from bodily fluids and fomites. J Infect Dis. 2007;196 Suppl 2:S142-7.
9. Towner JS, Rollin PE, Bausch DG, Sanchez A, Crary SM, Vincent M, et al. Rapid diagnosis of Ebola hemorrhagic fever by reverse transcription-PCR in an outbreak setting and assessment of patient viral load as a predictor of outcome. J Virol. 2004;78:4330-41.
10. Ebihara H, Rockx B, Marzi A, Feldmann F, Haddock E, Brining D, et al. Host response dynamics following lethal infection of rhesus macaques with Zaire ebolavirus. J Infect Dis. 2011;204 Suppl 3:S991-9.
11. Rollin PE, Bausch DG, Sanchez A. Blood chemistry measurements and D-Dimer levels associated with fatal and nonfatal outcomes in humans infected with Sudan Ebola virus. J Infect Dis. 2007;196 Suppl 2:S364-71.
12. Wauquier N, Becquart P, Padilla C, Baize S, Leroy EM. Human fatal zaire ebola virus infection is associated with an aberrant innate immunity and with massive lymphocyte apoptosis. PLoS Negl Trop Dis. 2010;4:
13. Oestereich L, Ludtke A, Wurr S, Rieger T, Munoz-Fontela C, Gunther S. Successful treatment of advanced Ebola virus infection with T-705 (favipiravir) in a small animal model. Antiviral Res. 2014;105:17-21.
14. Qiu X, Wong G, Audet J, Bello A, Fernando L, Alimonti JB, et al. Reversion of advanced Ebola virus disease in nonhuman primates with ZMapp. Nature. 2014;
15. Warren TK, Wells J, Panchal RG, Stuthman KS, Garza NL, Van Tongeren SA, et al. Protection against filovirus diseases by a novel broad-spectrum nucleoside analogue BCX4430. Nature. 2014;508:402-5.
16. Sayburn A. WHO gives go ahead for experimental treatments to be used in Ebola outbreak. BMJ. 2014;349:g5161.
17. Jovic G, Whitehead J. An exact method for analysis following a two-stage phase II cancer clinical trial. Stat Med. 2010;29:3118-25.
18. Clopper CJ, Pearson ES. The use of confidence or fiducial limits illustrated in the case of the binomial. Biometrika. 1934;26:404-13.
19. Bross IJ, Delaney MM. Significance tests for certain 2 x 2 tables. Am J Hyg. 1952;55:357-62.
20. Whitehead J. The Design and Analysis of Sequential Clinical Trials. Revised 2nd edition ed. Chichester: Wiley; 1997.
21. Whitehead J, Todd S. The double triangular test in practice. Pharmaceutical Statistics. 2004;3:39-49.
22. Fairbanks K, Madsen R. P values for tests using a repeated significance test design. Biometrika. 1982;69:69-74.
23. WHO. Ethical considerations for use of unregistered interventions for Ebola viral disease. Report of an advisory panel to WHO. Geneva, Switzerland: WHO; 2014 [9th September 2014]; Available from: http://apps.who.int/iris/bitstream/10665/130997/1/WHO_HIS_KER_GHE_14.1_eng.pdf?ua=1.

## 18. APPENDIX A: TRIAL FLOW CHART

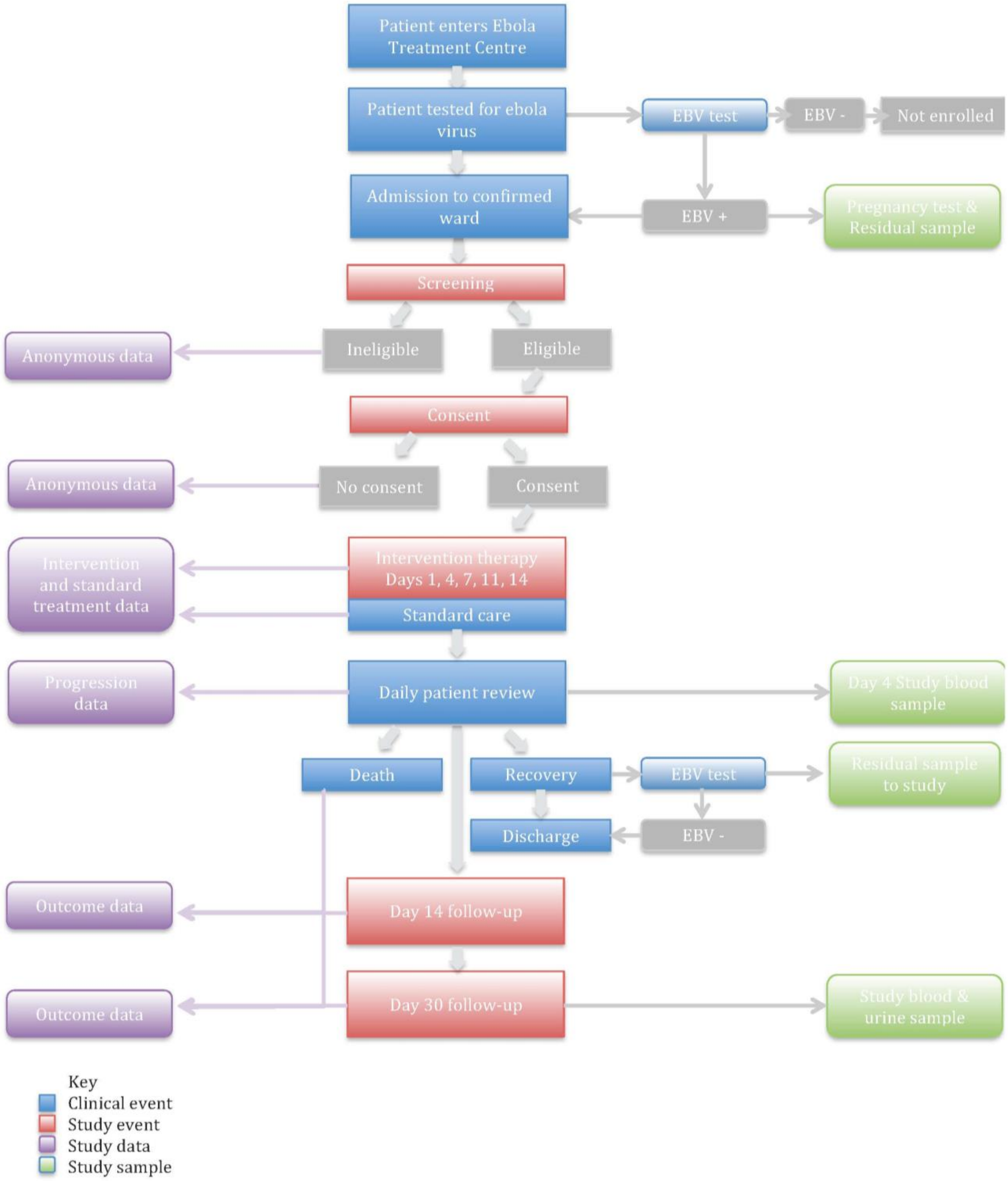

## 19. APPENDIX B: DOSING VOLUMES FOR DISOLVED TABLETS

Patients weighing less than 50 kg should have tablets dissolved in a closed container with 20 mL of water, shaken vigorously for 30 seconds and the appropriate volume drawn up into a graduated syringe. The table below shows the number of pills and volume of solution appropriate for each dose and each weight. Doses should be rounded up to the nearest mL.

- Initial dose of 4mg/kg = **2 tablets dissolved in 20 mL water**

followed by

- Four doses of 2mg/kg =**1 tablet dissolved in 20 mL water**

| Patient weight (kg) | Dose volume | Rounded dose volume (mL) |
|---|---|---|
| 3 | **1.2** | **2** |
| 4 | **1.6** | **2** |
| 5 | **2** | **2** |
| 6 | **2.4** | **3** |
| 7 | **2.8** | **3** |
| 8 | **3.2** | **4** |
| 9 | **3.6** | **4** |
| 10 | **4** | **4** |
| 11 | **4.4** | **5** |
| 12 | **4.8** | **5** |
| 13 | **5.2** | **6** |
| 14 | **5.6** | **6** |
| 15 | **6** | **6** |
| 16 | **6.4** | **7** |
| 17 | **6.8** | **7** |
| 18 | **7.2** | **8** |
| 19 | **7.6** | **8** |
| 20 | **8** | **8** |
| 21 | **8.4** | **9** |
| 22 | **8.8** | **9** |
| 23 | **9.2** | **10** |
| 24 | **9.6** | **10** |
| 25 | **10** | **10** |

| Patient weight (kg) | Dose volume | Rounded dose volume (mL) |
|---|---|---|
| 26 | **10.4** | **11** |
| 27 | **10.8** | **11** |
| 28 | **11.2** | **12** |
| 29 | **11.6** | **12** |
| 30 | **12** | **12** |
| 31 | **12.4** | **13** |
| 32 | **12.8** | **13** |
| 33 | **13.2** | **14** |
| 34 | **13.6** | **14** |
| 35 | **14** | **14** |
| 36 | **14.4** | **15** |
| 37 | **14.8** | **15** |
| 38 | **15.2** | **16** |
| 39 | **15.6** | **16** |
| 40 | **16** | **16** |
| 41 | **16.4** | **17** |
| 42 | **16.8** | **17** |
| 43 | **17.2** | **18** |
| 44 | **17.6** | **18** |
| 45 | **18** | **18** |
| 46 | **18.4** | **19** |
| 47 | **18.8** | **19** |
| 48-49 | **19.2** | **20** |

## 20. APPENDIX C: AMENDMENT HISTORY

| Amendment No. | Protocol Version No. | Date issued | Author(s) of changes | Details of Changes made |
|---|---|---|---|---|
| | | | | |

**Trial Title: Rapid Assessment of Potential Interventions & Drugs for Ebola (RAPIDE) - BCV**

**Scientific Title:** Open-label, non-randomised single arm trial to investigate the efficacy of Brincidofovir compared to historic controls for Ebolavirus Disease in an outbreak setting in West Africa.

**Short title:** Evaluation of BCV Treatment for Ebolavirus Disease

**Date and Version No**: 01 December 2014, V2.8


**Chief Investigator:** **Peter Horby**
Centre for Tropical Medicine and Global Health
Nuffield Department of Medicine Research Building
University of Oxford
Old Road Campus, Roosevelt Drive
OXFORD, OX3 7FZ

**Country Principal Investigator:** **Stephen Kennedy**
Pacific Institute for Research and Evaluation
University of Liberia
Capitol Hill
Monrovia, Liberia

**Co-Investigators:**

**Iza Ciglenecki** Médecins Sans Frontières Operational Center Geneva

**Annick Antierens** Médecins Sans Frontières Operational Center Brussels

**Laboratory Lead,** to be determined

**Trudie Lang** Global Health Network, Centre for Tropical Medicine and Global Health, University of Oxford

**Piero L Olliaro** Newton-Abraham Visiting Professor, University of Oxford.

**Jake Dunning (Field Clinical Lead)** Centre for Tropical Medicine and Global Health, University of Oxford

**John Whitehead** Department of Mathematics and Statistics, Lancaster University



**Sponsor:** University of Oxford

Joint Research Office, Block 60, Churchill Hospital, Oxford, OX4 7LE

**Funder:** Wellcome Trust

**Pan African Clinical Trial Registry** **PACTR201411000939962**

**TABLE OF CONTENTS**


1. KEY TRIAL CONTACTS ..... 4
2. SYNOPSIS ..... 6
3. ABBREVIATIONS ..... 7
4. INTRODUCTION ..... 7
5. OBJECTIVES AND OUTCOME MEASURES ..... 9
6. TRIAL DESIGN ..... 10
7. PARTICIPANT IDENTIFICATION ..... 12
   7.1. Trial Participants ..... 12
   7.2. Inclusion Criteria ..... 12
   7.3. Exclusion Criteria ..... 12
8. TRIAL PROCEDURES ..... 12
   8.1. Screening ..... 13
   8.2. Informed Consent ..... 13
   8.3. Assessments ..... 13
   8.4. Follow Up Assessments (Day 14 and Day 30) ..... 14
   8.5. Laboratory Assessments ..... 14
   8.6. Discontinuation of Trial Treatment ..... 15
   8.7. Withdrawal from the Study ..... 15
9. Trial Therapy ..... 15
   9.1. Investigational Medicinal Product ..... 15
   9.2. Dosing ..... 16
   9.3. Treatment Interruption ..... 16
   9.4. Storage and Accountability ..... 16
   9.5. Supportive Therapy ..... 17
10. SAFETY REPORTING ..... 17
   10.1. Definitions for safety reporting ..... 17
   10.2. Procedures for safety reporting ..... 17
11. STATISTICS ..... 18
   11.1. Description of Statistical Methods ..... 18
   11.2. The Number of Participants ..... 19
   11.3. Criteria for the Termination of the Trial ..... 19
   11.4. Final Analysis ..... 21

11.5. Inclusion in Analysis ........ 21
11.7 Procedure for Accounting for Missing, Unused, and Spurious Data. ........ 21
11.8 Rationale for statistical design choice ........ 21
12. DATA MANAGEMENT ........ 22
12.1. Data Capture ........ 22
12.2. Access to Data ........ 22
13. QUALITY ASSURANCE PROCEDURES ........ 22
14. ETHICAL AND REGULATORY CONSIDERATIONS ........ 23
14.1. Regulations, Guidelines and Approval ........ 23
14.2. Considerations in Informed Consent ........ 24
14.3. Participant Confidentiality ........ 25
14.4. Sample Management ........ 25
14.5. Risks and Benefits ........ 26
14.6. Expenses ........ 27
14.7. Contemporaneous Studies ........ 27
14.8. Alternatives to Study Participation ........ 28
14.9. Community Engagement ........ 28
14.10. Drug Access Post-Study ........ 28
15. FINANCE AND INSURANCE ........ 29
15.1. Funding ........ 29
15.2. Insurance ........ 29
16. PUBLICATION POLICY ........ 29
17. REFERENCES ........ 29
18. APPENDIX A: TRIAL FLOW CHART ........ 31
19. APPENDIX B: DOSING VOLUMES FOR DISOLVED TABLETS ........ 32
20. APPENDIX C: AMENDMENT HISTORY ........ 33

## 1. KEY TRIAL CONTACTS

| | |
|---|---|
| **Chief Investigator** | Associate Professor Peter Horby<br><br>Centre for Tropical Medicine and Global Health<br>Nuffield Department of Medicine Research Building<br>University of Oxford<br>Old Road Campus, Roosevelt Drive<br>OXFORD<br>OX3 7FZ<br><br>Tel: 07990 560237<br>Email: Peter.horby@ndm.ox.ac.uk |
| **Sponsor** | University of Oxford<br>Joint Research Office<br>Block 60, Churchill Hospital<br>Oxford, OX4 7LE |
| **Statistician** | Professor John Whitehead<br><br>Department of Mathematics and Statistics<br>Fylde College<br>Lancaster University<br>Lancaster LA1 4YF<br><br>Tel: 01524 389967<br>Email: j.whitehead@lancaster.ac.uk |
| **Committees** | **Trial Steering Committee Voting Members:**<br><br>**Samuel Kargbo** (Sierra Leone Representative)<br>Ministry of Health and Sanitation, Freetown, Sierra Leone<br><br>**Mandy Kader Konde** (Guinea Representative)<br>Commission Recherche Ebola, Conakry, Guinea<br><br>**Stephen Kennedy** (Liberia Representative)<br>Pacific Institute for Research and Evaluation, Monrovia, Liberia<br><br>**Fred Binka** (Independent Member)<br>University of Health and Allied Sciences, Ho, Ghana<br><br>**Peter Horby** (Chief Investigator)<br>Centre for Tropical Medicine and Global Health, Oxford, United Kingdom<br>International Severe Acute Respiratory and emerging Infections Consortium<br><br>**Annick Antierens** (MSF Representative)<br>Médecins Sans Frontières, Geneva, Switzerland<br><br>**John Whitehead** (Trial Statistician) |

| | |
|---|---|
| | Lancaster University, Lancaster, United Kingdom<br><br>**Oumou Younoussa Bah-Sow** (Independent Member)<br>Hopital National Ignace Deenm, Guinea<br><br>**Rob Fowler** (Independent Member)<br>Sunnybrook Health Sciences Centre, Toronto, Canada<br><br>**Nicholas White** (Independent Member - Chairperson)<br>Mahidol-Oxford Research Unit, Bangkok, Thailand<br><br>**Non-Voting Members**<br>**Ana Maria Henao-Restrepo** (WHO Representative)<br>World Health Organization, Geneva, Switzerland<br><br>**Independent Data Monitoring Committee (IDMC) Members:**<br><br>**Liberian National IDMC member,** Dr Garfee Williams<br><br>**David Lalloo** (Chair), Professor of Tropical Medicine; Dean of Clinical Sciences and International Public Health, Liverpool School of Tropical Medicine<br><br>**Clement Adebamowo,** Director Office of Strategic Information and Research Institute of Human Virology in Nigeria<br><br>**Donald Berry,** Founder and senior Statistical Scientist, Berry Consulting<br><br>**Nicolas Opoku,** Director Onchocerciasis Chemotherapy Research Centre, Hohoe, Ghana<br><br>**Pontiano Kaleebu,** Director MRC/UVRI Uganda Research Unit on AIDS<br><br>**Peter Smith,** Professor of Tropical Epidemiology, London School of Hygiene and Tropical Medicine – to be confirmed |

## 2. SYNOPSIS

| | | |
|---|---|---|
| Trial Title | **Rapid Assessment of Potential Interventions & Drugs for Ebola (RAPIDE) - BCV** | |
| Scientific Title | Open-label, non-randomised single arm trial to investigate the efficacy of Brincidofovir compared to historic controls or Ebolavirus Disease in an outbreak setting in West Africa | |
| Internal ref. no. (or short title) | Evaluation of BCV Treatment for Ebolavirus Disease | |
| Clinical Phase | 2 | |
| Trial Design | Open-label, non-randomised, single arm trial | |
| Trial Participants | Patients with confirmed Ebolavirus disease attending the participating treatment centre | |
| Planned Sample Size | Up to 140 adult participants, paediatric enrolment according to presentation over the same recruitment period | |
| Treatment duration | 2 weeks | |
| Follow up duration | 30 days follow-up | |
| | Objectives | Outcome Measures |
| Primary | To evaluate the impact of BCV treatment on early mortality in EVD | Mortality at Day 14 |
| Secondary | 1. To evaluate the impact of BCV treatment of adults and children on:<br>a) Time to recovery<br>b) Late mortality<br>c) Viral load<br>d) EVD symptoms<br>e) EVD antibody response<br><br>2. To assess the safety of BCV treatment of adults and children | 1. a) Time to meeting EVD treatment center discharge criteria.<br>b) Mortality at Day 30 after first dose of study treatment<br>c) Day 4 viral load<br>d) Presence and duration of symptoms (Days 0-14)<br>e) Convalescent anti-Ebolavirus IgG titer (Day 30)<br><br>2. Incidence of SARs and key adverse events (Days 0-14) |
| Investigational Medicinal Product | Brincidofovir (BCV) | |
| Formulation, Dose, Route of Administration | 100 mg tablet for oral administration. May be dissolved in water if patient is unable to swallow pills.<br>Patients ≥50 kg: 200 mg initial dose on Day 0, then 100 mg on Days 3, 7, 10, and 14<br>Patients <50 kg: 4mg/kg on Day 0, then 2 mg/kg on Days 3, 7, 10, and 14 | |

## 3. ABBREVIATIONS

| | |
|---|---|
| BCV | Brincidofovir (BCV) |
| CRF | Case Report Form |
| EDTA | Ethylenediaminetetraacetic acid |
| ETC | Ebola Treatment Centre |
| EVD | Ebolavirus Disease |
| GCP | Good Clinical Practice |
| IB | Investigators Brochure |
| ICF | Informed Consent Form |
| ICH | International Conference on Harmonisation |
| IDMC | Independent Data Monitoring Committee |
| IMP | Investigational Medicinal Product |
| MSF | Medecins Sans Frontieres |
| OXTREC | Oxford Tropical Research Ethics Committee |
| PI | Principal Investigator |
| REC | Research Ethics Committee |
| RNA | Ribonucleic acid |
| RT-PCR | Reverse transcription polymerase chain reaction |
| SAE | Serious Adverse Event |
| SAR | Serious Adverse Reaction |
| SUSAR | Suspected Unexpected Serious Adverse Reactions |
| TSC | Trial Steering Committee |
| WHO | World Health Organisation |

## 4. INTRODUCTION

The size and scale of the on-going Ebola Virus Disease (EVD) outbreak is unprecedented, shows no signs of abating, and has been declared a Public Health Emergency of International Concern.[1] EVD is among the most virulent infectious agents known: an analysis of data on 3343 confirmed and 667 probable EVD cases collected during the current outbreak in Guinea, Liberia, Nigeria, and Sierra Leone estimates a case fatality rate of 70.8% (95% confidence interval [CI], 69 to 73) among persons with known clinical outcome of infection.[2] A figure that is consistent with estimates of other authors.[3] **Figure 1** shows the key time points in the course of EVD in this outbreak.[2]

**Figure 1. Estimated mean (median) time course in days of Ebolavirus infection in this outbreak.[2]**

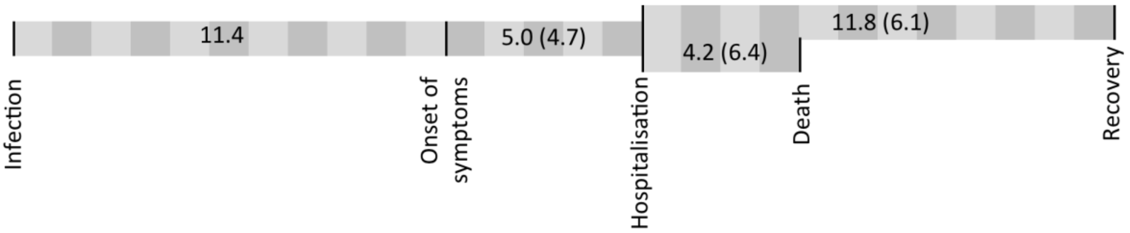


The current Ebolavirus strain causing the outbreak in West Africa is related to the Ebola Zaire strain and genetic analysis suggests the current outbreaks in Guinea, Sierra Leone and Liberia are all related to a single transmission event from a zoonotic source followed by human-to-human transmission.[4] EVD is characterized by a febrile illness dominated by fatigue and gastrointestinal symptoms that can be complicated by shock, haemorrhage and multi-organ failure. However, clinical presentation and severity is variable, with some patients remaining relatively well, able to ambulate and self-feed throughout their illness, whilst others progress rapidly to a fatal outcome.[2, 5]

The pathogenesis of Ebola Virus Disease is incompletely understood [6, 7] but high levels of viral replication and the detection of virus in multiple body tissues is typical of severe disease.[8, 9] Coagulopathy, disruption of endothelial function and increased inflammatory responses are also associated with severe EVD.[6, 10-12] The association between high levels of viraemia and EVD severity suggests that therapies that target viral replication may benefit patients. Several experimental products that target Ebolavirus replication have shown some efficacy in animal studies.[13-15] However, whilst several therapeutic interventions have shown promise in the laboratory and in animal studies, none have been tested for efficacy and safety in humans with EVD. A World Health Organization (WHO) expert panel recently concluded unanimously that "investigators have a moral duty to evaluate these interventions in the best possible clinical studies that can be conducted under the circumstances of the epidemic."[16]

The aim of this protocol is to assess the effectiveness of Brincidofovir (BCV, produced as CMX001 by Chimerix Inc.) for the treatment of EVD. BCV is an orally bioavailable, lipid acyclic nucleotide that was designed to utilize endogenous lipid uptake pathways to achieve high intracellular concentrations of BCV. BCV is a lipid conjugate of the nucleotide analogue cidofovir. BCV has demonstrated broad spectrum antiviral activity against various pathogenic human double stranded DNA (dsDNA) viruses in vitro and in animal models. BCV has successfully completed Phase 2 clinical development for the prevention of cytomegalovirus infection. A Phase 3 study of cytomegalovirus prevention in adult haematopoietic cell transplant recipients, Study CMX001-301 (also known as the SUPPRESS trial), began enrolling subjects in 2013. In addition, BCV is in Phase 2 clinical development for preemptive treatment of adenovirus viremia. In cytomegalovirus -infected haematopoietic cell transplant subjects, the main dose-limiting toxicity has been gastrointestinal in nature, primarily diarrhea, when administered at total weekly doses greater than 200 mg. In addition, dose-related, transient, asymptomatic serum aminotransferase increases (primarily alanine transaminase, but also aspartate transaminase) have been reported in both virally-infected subjects and healthy subjects, but do not appear to be of toxicologic importance. There is no evidence of the nephrotoxicity, myelotoxicity and ocular toxicity associated with the intravenous administration of cidofovir following once or twice weekly oral administration of BCV. The safety database of over 1000

individuals exposed to BCV has shown no evidence of hematologic toxicity or nephrotoxicity. No dose adjustment of BCV is necessary for renal or hepatic impairment. The potential for drug-drug interactions mediated through cytochrome P450 enzymes is low. BCV should be considered a potential carcinogen and should not be administered for longer than 3 months unless the potential benefit justifies the potential risk to patients.

BCV was tested for activity against the Mayinga strain of Ebola Zaire in Huh7 cells at the US Centers for Disease Control and Prevention. BCV was added to cells at 48, 24 and 2 hours prior to infection. The half maximal effective concentration (EC50) ranged from 21 nM with 48 hour pretreatment to 524 nM with a 2 hour pretreatment with therapeutic indices ranging from 54 to >2300. These EC50s encompass the range of EC50s observed for dsDNA viruses, which have been effectively treated with BCV in humans and animal models (adenovirus, poxviruses). Additional assessments of BCV in mouse models of Ebolavirus infection are underway. The pre-clinical work indicating potential efficacy combined with experience in over 1000 patients to show efficacy in other viral infections and providing safety and tolerability data, support the case for undertaking a phase IIB assessment of this drug in patients presenting with EVD.

Reproductive studies of BCV have identified embryotoxicity and fetal morphological changes in rabbits, and decreased fertility, embryonal viability, and growth and development of pups with delayed sexual maturation in rats. Given the current lack of data on the efficacy of BCV treatment in humans infected with Ebolavirus, the theoretical risk of embryotoxicity precludes the administration of BCV to pregnant women.

The Trial Steering Committee selected BCV for urgent evaluation from a possible list of 10 candidate therapies. The criteria for selection were based upon a Target Product Profile that considered existing clinical and pre-clinical data supporting safety and efficacy, immediate availability and scalability, and an acceptable treatment regimen.

The purpose of this trial is to establish whether BCV is (a) very effective, (b) promising or (c) ineffective. Details of how these three states are characterized and how BCV will be classified based on the data from this trial are given in Section 11 below. If conclusion (a) is reached, we recommend that the high effectiveness of BCV is confirmed in a subsequent single-arm trial of at least 100 patients. If conclusion (b) is reached, we recommend that the promise of BCV is studied further in a randomized comparison with best supportive care. If conclusion (c) is reached, we recommend that other treatments are given priority in clinical research, but that BCV might be reconsidered if better treatments are not found. We are developing trial designs for suitable single-arm confirmatory and randomized comparative trials that will ensure low risks that the sequence of drug development will give invalid conclusions.

It is the intention to use this clinical trial to build a platform for investigation of a number of experimental interventions for the treatment of EVD. As additional therapies become available, and are deemed appropriate for evaluation, this protocol will be replicated to test additional investigational medicinal products in multiple single arm studies.

## 5. OBJECTIVES AND OUTCOME MEASURES

| Objectives | Outcome Measures |
| --- | --- |

| | |
|---|---|
| **Primary Objective**<br>To evaluate the impact of BCV on early mortality in EVD | Mortality at Day 14 after entry into the trial |
| **Secondary Objectives**<br>1) To evaluate the impact of BCV treatment of adults and children on:<br><br>a) Time to recovery<br><br>b) Late mortality<br><br>c) Viral load<br><br>d) EVD symptoms<br><br>e) EVD antibody response<br><br>2. To assess the safety of BCV treatment of adults and children | Secondary outcomes measures:<br>1. a) Time to meeting EVD treatment center discharge criteria<br><br>b) Mortality at Day 30 after first dose of study treatment<br><br>c) Day 4 viral load<br><br>d) Presence and duration of symptoms (Days 0-14)<br><br>e) Convalescent anti-Ebolavirus IgG titer (Day 30)<br><br>2. Incidence of SARs and key adverse events (Days 0-14) |

## 6. TRIAL DESIGN

This is an open-label, non-randomised, single arm trial, which will be conducted in Monrovia, Liberia. Collated retrospective data will be used to inform mortality targets for comparison of trial data. Patients with reverse transcription polymerase chain reaction (RT-PCR) confirmed Ebolavirus RNA (confirmed patients) are admitted to confirmed wards of the Ebola Treatment Centre. Confirmed patients will be screened for trial eligibility and invited to give informed consent to participate. Parents/guardians of children and representatives of severely ill patients will be invited to give consent on behalf of the patient where appropriate. Study dosing, sampling and data collection will begin after enrolment. **Table 1** shows the schedule of study procedures.

The primary objective of the RAPIDE clinical trial team is to assess a number of potential treatments for Ebolavirus disease and to triage them as (a) very effective, (b) promising and (c) showing little evidence of effectiveness. The RAPIDE strategy is therefore intended to find and assess a very effective treatment, if one exists, as quickly as possible, and in time to help the patients in the current outbreak. RAPIDE-BCV as described in this protocol, is the first single-arm triage study and will be followed by additional studies that will be designed based on the results of this first trial and the availability of investigational products. As such, RAPIDE-BCV is the first stage of a larger 'adaptive' study. Follow-on studies will be designed and submitted for ethical and regulatory approval as data and products become available.

*Table 1. Schedule of study procedures*

| Procedures | Screening | Enrolment Day 0 | In-Patient visit per day | Day 4 (+/- 24 hours) | Discharge | Day 14 | Day 30 |
|---|---|---|---|---|---|---|---|
| Eligibility assessment | x | | | | | | |
| Pregnancy test | x (standard care) | | | | | | x |
| Informed consent | x | | | | | | |
| Demographics , co-existing morbidities, clinical history | | x | | | | | |
| Clinical evaluation including key adverse events | | x | x | | | | |
| Primary outcome assessment | | | | | | x | x |
| SAR assessment | | | x | | | | |
| Record of other therapy | | x | x | | | | |
| Administration of BCV* | | x | x x x (days 3, 7, 10) | | | x | |
| Routine blood draws** ≤4 mls EDTA, ≤4 mls EDTA/lithium heparin ≤4 mls serum tube | x | | | | x | | |
| Study blood draws*** Day 4: ≤4 mls EDTA, ≤4 mls EDTA/lithium heparin, ≤4 mls serum tube Day 30: ≤4 mls EDTA, ≤4 mls serum tube | | | | x | | | x |

*Study treatment is planned for days 0, 3, 7, 10 and 14. Days may be altered if required (see section on dosing below). Patients who meet discharge criteria and leave the ETC before Day 14 will stop study treatment upon discharge from the ETC.

**Screening – Ebolavirus diagnostic PCR, serology, store residual. More than one diagnostic PCR or ICT may be done if required for standard care. FBC, LFTs, glucose, urea and electrolytes will be analysed if established as standard care by the site.

**Discharge – Ebolavirus PCR to confirm of viral clearance, serology, store residual. More than one confirmatory PCR may be done if required for standard care. FBC, LFTs, glucose, urea and electrolytes will be analysed if established as standard care by the site. Pharmacokinetics will be analysed when possible.

***Day 4 – Pharmacokinetics, Ebolavirus PCR, serology, store residual. FBC, LFTs, glucose, urea and electrolytes will be analysed on Day 4 if established as standard care at screening by the site.

*** Day 30 – Serology and pregnancy test

The maximum amount of blood that will be obtained on any particular day for adult and pediatric patients is 14 mL. The total maximum amount of blood that will be obtained for research purposes over the full duration of the study is 21 mL for adult and pediatric patients. The amount of blood taken for standard care over the full duration of the study is up to 35 mL. Adult and pediatric samples will be reduced in volume according to standard procedures such that no patient will have more than 0.6 mls/kg (>1% blood volume) taken on any one day, nor more that 2.4 mls/kg (approximately 3% of blood volume) taken in any four week period. Standard care samples will be prioritized over research samples if volume reduction is required. Ability to take samples is dependent on staff availability and caseload. Research samples and standard care samples may be reduced where required, to maintain care standards and staff safety, and reflect the assays that can be performed by the laboratory attached to the ETC. Other chemical or biological assays may be performed using the same blood volumes if deemed necessary to inform patient safety or study outcomes.

# 7. PARTICIPANT IDENTIFICATION

## 7.1. Trial Participants

Participants with laboratory confirmed EVD treated at a participating Ebola Treatment Centre and meeting the inclusion and exclusion criteria will be eligible to take part in the study.

## 7.2. Inclusion Criteria

- Male and female patients 2 months of age or older
- Laboratory confirmed infection with Ebolavirus infection.
- Women who are lactating must agree to stop breastfeeding (a standard recommendation for all EVD patients)
- Willingness to use barrier birth control for 3 months after recovery of EVD (a standard recommendation for all EVD patients)
- For patients 18 years and older: the ability to provide fully informed consent or the provision of consent from the representative of a patient deemed too unwell to comprehend what is being asked of them.
- For patients under 18 years: consent from a parent or guardian.

## 7.3. Exclusion Criteria

The participant may not enter the trial if the following applies:

- Any underlying disease or condition that, in the opinion of the principal investigator or treating physician, could jeopardize the participant's or healthcare workers safety or the participant's ability to comply with the protocol requirements
- Patient unable to take oral IMP (e.g. severe vomiting unresponsive to anti-emetics, unconscious)
- Pregnancy

# 8. TRIAL PROCEDURES

## 8.1. Screening

All patients with laboratory confirmed EVD will be considered for study participation. Research staff will work with clinical staff to evaluate all patients for inclusion/exclusion criteria based on available clinical information. Pregnancy tests are done as a part of routine standard of care at the Liberia study site using the same blood taken for Ebolavirus diseases diagnosis. Women who are identified as pregnant will be informed by the treating staff and will not be enrolled to the study. Women who have a positive pregnancy test, but have aborted/miscarried or given birth within the previous month will not be excluded. Staff will not discuss informed consent with patients (or the parents/representatives of patients <18 years old) with underlying disease that prohibits study inclusion or those unable to take oral IMP. The number of patients who meet these exclusion criteria will be recorded anonymously.

## 8.2. Informed Consent

Patients who have not been excluded based on the initial screening will be invited to discuss study participation. A patient may be enrolled in the study anytime within 24 hours of being informed of Ebolavirus positive PCR results. Therefore, if a patient has been excluded for a reason that resolves within this period, they may be approached upon resolution of the reason for exclusion. If a patient wishes to delay their decision regarding consent, they may enrol at any time within the 24 hour period.

Study participation will be discussed with adult patients who, by the judgement of the treating staff are sufficiently mentally and physically fit to provide such consent. Where the participant is deemed too unwell, the study will be discussed with a representative of the patient. In the case of children, the study will be discussed with a parent/guardian or appropriate representative in the absence of a parent/guardian. For the purpose of consent, a child is defined as a patient less than 18 years of age. For the purpose of consent, an adult is a patient ≥18 years of age.

Written informed consent will be obtained from all patients or parents/guardians/representatives who agree to study participation. In the event of consent being given by the representative of a severely ill adult patient, the patient will be approached for consent if they gain the ability to discuss the study at a later time during their participation. The representative may be a personal consultee of the patient or, if a personal consultee is not available, a professional consultee, defined as a designated site clinician who is not directly involved in the research study.

The number of patients who refuse to give informed consent will be recorded anonymously. Further details of informed consent considerations and procedures can be found in section 14.2.

## 8.3. Assessments

All patients who meet inclusion and exclusion criteria will be enrolled. Study data will be collected on key symptoms, clinical characteristics and administration of all medications including BCV treatment. Diarrhoea and vomiting will be specifically monitored for changes in severity following administration of BCV as they are recognised dose-limiting side effect. These data will be collected daily for participating patients up to Day 14, or death, or discharge, whichever occurs first.

Discharge criteria are:

- 72 hours without fever or significant symptoms AND
- A significant improvement in clinical condition AND
- Able to feed, wash and walk independently AND

- Ebolavirus RNA PCR negative.

The first day of study treatment administration is defined as Day 0. Patients who achieve discharge criteria before day 14 will stop study treatment upon discharge.

### 8.4. Follow Up Assessments (Day 14 and Day 30)

Survival at Day 14 is the primary end point of this trial, and defined as survival until past midnight from Day 13. Follow up assessments will be conducted on Days 14 and 30. Patients who have been discharged before either of these dates will be issued a mobile telephone for the purposes of follow-up. Patients will be invited to return to the treatment centre for these visits. When patients are not able or willing to attend a follow-up visit at the treatment centre, field workers will endeavour to visit patients in their home if agreed by the patient. If a visit to the treatment centre and a field worker visit are both prohibitive (e.g. due to the patient living very far from the treatment centre), staff will follow-up with the patient by telephone.

Patient status on follow-up assessment days will be collected (admitted, met discharge criteria, deceased). A blood sample (≤5 mL) will be collected on Day 30 for assessment of convalescent anti-Ebolavirus IgG titer. Samples that cannot be collected on Day 30, but +/- 5 days will be accepted and analysed appropriately.

Primary/secondary outcome data for Day 14 and 30 can be collected at any point after the respective days. Any pregnancies which occur during the study period will be followed to obtain outcome.

### 8.5. Laboratory Assessments

Malaria immunochromatographic diagnostic tests will be performed on the triage blood sample as part of standard care at the Liberia study site. The results of these tests will be recorded.

For women of childbearing age (15-49 years) a chromatographic βHCG test will be performed on the triage and Day 30 blood samples. Urine testing is also acceptable.

Patients will have EDTA blood samples collected at triage for EVD diagnosis by PCR or ICT, prior to discharge to confirm clearance of viral RNA, and also for virus detection on Day 4 (+/- 24 hours). Anti-Ebolavirus IgG will be evaluated at each of these study days and additionally on Day 30. The triage and discharge samples are a part of standard care at the Liberia study site and may be repeated if required. The Day 4 and Day 30 samples are for study purposes only. The PCR result for all samples, including the day 4 sample will be shared with the clinical team in real time. Residual volumes of all samples will be stored for study analysis. The triage sample will have been obtained as a part of standard care, before the patient has provided informed consent; residual volume from this sample will be used for study purposes and the results of the PCR and other blood tests will be recorded.

Samples obtained for PCR are typically ≤4 ml whole blood in an EDTA tube; finger or heel-pricks of blood on a dry swab are sometimes obtained when venepuncture is not possible. Ebolavirus RNA will be detected by PCR, as per local clinical laboratory protocols, for the triage, day 4 and discharge samples. Additionally, plasma anti-Ebolavirus antibody titres will be determined by analysis of residual volumes by partner laboratories. This is to determine whether the treatment influences the subsequent production of specific antibodies.

Standard care for EVD patients at the Liberia study site currently includes blood draws at admission and discharge which include Ebolavirus PCR testing. If the capabilities are established, additional laboratory tests for patient care at these routine time points will include a screening full blood count, liver function tests, urea and electrolytes and glucose (≤4 ml EDTA/lithium heparin, ≤4 ml serum tube, ≤2 ml potassium oxalate/sodium fluoride tube). In the event of establishment as a part of clinical care, these tests will be repeated on Day 4 and at discharge for study purposes. Pharmacokinetic analysis will also be performed on the Day 4 and Discharge samples for research purposes.

Aliquots of residual plasma from the same samples will be stored at -20°C or colder and subsequently shipped to international partner laboratories for confirmatory testing and quantification. If volume remains, additional testing relevant to the pathogenesis of EVD or effects of the study treatment will be performed. Residual cells or plasma supernatant (buffy coat) may be retained for host genetic studies to identify susceptibility and severity markers. Details of sample storage, shipment and custody are in Section 14.4.

### 8.6. Discontinuation of Trial Treatment

Each participant has the right to discontinue use of study treatment at any time. In addition, the Investigator may discontinue treatment at any time if the Investigator considers it necessary for any reason including:

- Ineligibility (either arising during the trial or retrospectively having been overlooked at screening)
- An adverse event which requires discontinuation of the trial medication
- Disease progression which requires discontinuation of the trial medication
- Patient or physician decision

The reason for any discontinuation of treatment will be recorded in the case record form.

### 8.7. Withdrawal from the Study

Patients, or parents/guardians/representatives when they have given consent on behalf of the patient, are free to withdraw consent and stop study participation at any time for any reason. In this case, the patient's clinical management will continue to be provided according to standard care and will not be affected by the decision to withdraw. Outcome data and the reason for withdrawal will be recorded when possible. Data collected up to the date of withdrawal will be used in study analysis.

## 9. Trial Therapy

### 9.1. Investigational Medicinal Product

BCV has been provided to more than 400 patients with serious or life-threatening infections with dsDNA viral infections and no alternative treatment options under expanded access or emergency investigational new drug or local equivalent regulations in nine countries, including the United States, Canada, France, United Kingdom, Spain, Switzerland, Austria, Israel, and Peru. The safety database of over 1,000 individuals exposed to BCV has shown no evidence of hematologic toxicity or nephrotoxicity. BCV is progressing in two clinical development programs: one for the prevention of cytomegalovirus (CMV)

infection in hematopoietic cell transplant recipients, and the other for the treatment of adenovirus infection. Phase 3 studies (CMX001-301 or SUPPRESS Trial for CMV; CMX001-304 or AdVise Trial for adenovirus) are being conducted in the United States, Canada, and Belgium.

BCV is supplied as a 100 mg tablet for oral administration.

100 mg tablet: BCV tablets are formulated as dry-blend, direct compressed tablets containing 100 mg of BCV as free acid. In addition to the active ingredient, BCV tablets contain microcrystalline cellulose, mannitol, crospovidone, and magnesium stearate. Tablets have a re-test period of 2 years.

In the case of patients unable to swallow pills, the tablets will be dissolved in water. When possible, treatment should be taken with a meal.

## 9.2. Dosing

Patients weighing ≥50 kg will be administered a 200 mg loading dose (two 100 mg tablets) on Day 0, followed by 100 mg on Days 3, 7, 10 and 14 (notwithstanding adjustments as detailed below).

Patients weighing <50 kg will be administered a 4mg/kg initial dose on Day 0, then 2 mg/kg on Days 3, 7, 10 and 14 (notwithstanding adjustments as detailed below).

In the case of a dose not taken on the assigned study day, it should be taken as soon as possible. The following dose should be taken 3 days after the administration of the missed dose, and treatment continued every 3 days until completion of 5 doses.

Patients who cannot swallow tablets will receive tablet(s) dissolved in water. Tablets will be dissolved in 20 mL of water, which can be divided to give doses smaller than 100 mg according to weight (see dosing table in Appendix 3), and administered immediately. When possible, drug should be administered with a low fat meal. Full details of treatment preparation and administration will be outlined in the study procedures.

BCV will be administered under the supervision of clinical staff. Patients who vomit within 30 minutes of receiving treatment will receive an additional dose as soon as they are able. A maximum of 1 redose will be given for any individual dose. In the event of a redose being vomited within 30 minutes of administration, patients will receive the next scheduled dose on the day after the vomited dose. Remaining dose(s) will be taken every three days until completion of 5 doses. No dose adjustments will be made to account for possible effect of gastrointestinal symptoms on absorption. All administration of study drug will be recorded in the CRF.

## 9.3. Treatment Interruption

In the event of a SUSAR or other significant treatment related event, study investigators will determine if study treatment will continue on the basis of clinical picture and severity and resolution of the event. Consultation with the Independent Data Monitoring Committee will be made in the case of uncertainty to determine if the treatment should be discontinued.

## 9.4. Storage and Accountability

Study drug should be stored at controlled room temperature between 15 and 25°C, with excursions permitted up to 30°C. Inventory, dispensing and accountability of study treatment will be tightly controlled. Treatment dispensed to the Confirmed ward, but not consumed by patients will not be

returned due to infection control procedures and will be destroyed. Full accountability procedures and logs will be detailed in the corresponding study reference manual.

### 9.5. Supportive Therapy

The supportive therapy received by patients will not be affected by participation, or non-participation, in the trial. All patients will receive standard supportive therapy and this will be recorded. Treatment provided by clinical staff is based on the current MSF guidelines for management of EVD. This includes oral and/or intravenous fluid resuscitation, empirical antibiotics, antimalarial agents, and also symptom control, which includes antiemetics, antipyretics/analgesics, and anti-diarrhoeal agents, as appropriate to the patient's symptoms.

## 10. SAFETY REPORTING

It will not be possible to fully determine the safety profile of the study drug within this trial design because the sample size is insufficient. Due to the nature of the symptoms of EVD (see **Table 2**) [2] and the relatively small sample size it will be very difficult to differentiate between symptoms of the disease and events due to treatment, and the trial will have limited power to do this. Our investigation of safety will therefore focus on Serious Adverse Reactions (SARs) and Suspected Unexpected Serious Adverse Reaction (SUSARs) and key adverse events.

### 10.1. Definitions for safety reporting

**Serious Adverse Reaction (SAR)** – A serious untoward and unintended response in a participant to the study treatment, which is related (or has a reasonable possibility of being related) to any dose administered to that participant.

To qualify as "serious" the response must meet one of the following criteria:

- results in death
- is life-threatening
- requires inpatient hospitalisation or prolongation of existing hospitalisation
- results in persistent or significant disability/incapacity
- consists of a congenital anomaly or birth defect.

Other 'important medical events' may also be considered serious if they jeopardise the participant or require an intervention to prevent one of the above consequences.

**Suspected Unexpected Serious Adverse Reaction (SUSAR) -** A serious adverse reaction, the nature and severity of which is not consistent with the information about the medicinal product in question set out in the investigator's brochure (IB).

### 10.2. Procedures for safety reporting

All SARs will be reported by the site to the operational team within 48 hours. In discussion with the site staff, the operational team will assess the SAR for expectedness and relatedness and report all SUSARs to the independent data monitoring committee (IDMC) immediately. The operational team will also report any SUSARs to all the relevant parties as required within 7 days.

Evaluation of expectedness and relatedness of reactions will consider the opinion of the site staff and the expected signs, symptoms and events associated with Ebolavirus disease or BCV treatment according to the Investigators' Brochure. Evaluation of expectedness will be made with reference to the Investigator's brochure.

**Table 21. Symptoms of Ebolavirus disease (WHO Ebola Response Team, 2014[2]; ISARIC WHO Viral Haemorrhagic Fever CRF)**

| Known symptoms and clinical events of EVD: | |
|---|---|
| Death | Coma or unconsciousness |
| Fever | Unexplained bleeding |
| Fatigue | Hematemesis |
| Loss of appetite | Blood in stool |
| Vomiting | Bleeding gums |
| Diarrhoea | Bloody nose |
| Headache | Bloody cough |
| Abdominal pain | Other bleeding |
| Joint pain | Bleeding at injection site |
| Muscle pain | Blood from vagina |
| Chest pain | Blood in urine |
| Cough | Bleeding under the skin/bruising |
| Difficulty breathing | Back pain |
| Difficulty swallowing | Decreased urine output |
| Conjunctivitis | Lower chest wall indrawing |
| Sore throat | Hearing impairment |
| Confusion | Tinnitus |
| Hiccups | Seizures |
| Jaundice | Hepatomegaly |
| Eye pain | Splenomegaly |
| Rash | Lymphadenopathy |

Diarrhoea (and, less frequently, nausea, vomiting, anorexia, loss of appetite, hypoalbuminemia, and abdominal pain) and hepatobiliary events (including elevation of serum aminotransferases) are considered possible side effects associated with the use of BCV. These side effects overlap with some EVD disease manifestations as highlighted above. For diarrhoea and vomiting, severity and temporal association with study drug will be included in regular IDMC reports. Patients will be re-dosed if vomiting within 30 minutes of receiving treatment; otherwise no dose adjustments will be made to account for possible effect of gastrointestinal symptoms on absorption.

## 11. STATISTICS

### 11.1. Description of Statistical Methods

The trial is designed to distinguish between three situations: (a) the treatment is very effective, (b) the treatment is promising and (c) the treatment is ineffective. The effectiveness of the treatment will be

judged in terms of the probability that a treated adult patient will survive to Day 14, denoted by P. Note that p represents the true value of the probability rather than any estimate that might be found from the trial data. Three possible values of P are used for guidance. If P = 0.800 then the treatment will be regarded as very effective. If P = 0.667 then the treatment will be regarded as promising. If P = 0.500 then the treatment will be regarded as ineffective.

Both children and adults will be treated in this trial. However, they will be analysed separately as the effects of treatment may be different in the two groups. The primary analysis will concern the results from adults, and a secondary analysis of the data in children will be used to ascertain whether the conclusions from those adult patients should apply to children. The stopping rules governing the study will be based on data from adults only, and once met the whole study, in adults and children, will be stopped. In this section, sample sizes mentioned refer to the number of adult patients in the trial, and analyses refer to analyses of the data from adult patients only. For the purpose of analysis adults are defined as all patients ≥50kg in weight and/or ≥18 years of age.

The choice of guide values for P has been made following an analysis of individual level data on patients from the Gueckedou treatment centre. Data are available on the outcomes of 778 patients with laboratory confirmed Ebolavirus infection. Of these, 239 survived. In the 140 patients with ages available and less than 16 years (children) 27.1% survived, while in the other 638 patients (adults), 31.5% survived. This is a survival rate of 30.7%, which is consistent with rates reported elsewhere. Following calculation of this initial estimate, the sample was reduced to reflect the type of patient who would feature in the primary analysis of the trial. Only adult patients who were treated in the hospital itself, and had known dates of onset of symptoms and of death or discharge from hospital were included. This led to a sample size of 500 (all suitable patients were included, that the sample size is exactly 500 is coincidental). Failure was defined as dying on or before the 14th day after onset of symptoms, and success was taken to be lack of failure. Thus, patients dying after Day 14 were treated as successes, as will occur in the trial analysis. Of these 500 patients, 237 succeeded. This is a success rate of 47.0%. The probability of success for an untreated control can thus be estimated as 0.470, with a 95% confidence interval of (0.426, 0.514). The success rate was reasonably constant over the 8 months of operation of the Gueckedou treatment centre and did not appear to depend on the number of days between onset of symptoms and hospitalisation.

As a result of this analysis, P = 0.500 has been set as the success rate threshold for which the experimental treatment would be considered ineffective (i.e. ineffective if P < 0.500).

## 11.2. The Number of Participants

The maximum number of adult participants required to evaluate the efficacy is 140. The data will be analysed sequentially and stopping rules applied to each investigational product. It is most likely that the number of participants will be fewer than 100 (see 11.3).

## 11.3. Criteria for the Termination of the Trial

Research sites will inform the data management centre every time that an eligible patient is enrolled into the trial. Fourteen days later, they will report to the data management centre whether that patient did, or did not, survive to Day 14. Every time that a Day 14 report is received on an adult patient, a point will be plotted on Figure 2. This is plot of the number of (adult) survivals reported so far against the number of (adult) Day 14 reports received. The plot is compared with one of the three boundaries. As soon as the plot reaches one of the boundaries, the trial will be stopped. Conclusion (a), (b) or (c) will be drawn according to whether the green, orange or red boundary is reached. The trial would stop with conclusion

(a) if all of the first 24 patients recruited survived to Day 14, and with conclusion (c) if none of the first 12 patients recruited survived to Day 14. The stopping boundaries are truncated at 140: this is the maximum sample size.

**Figure 2.** Stopping rules for the trial

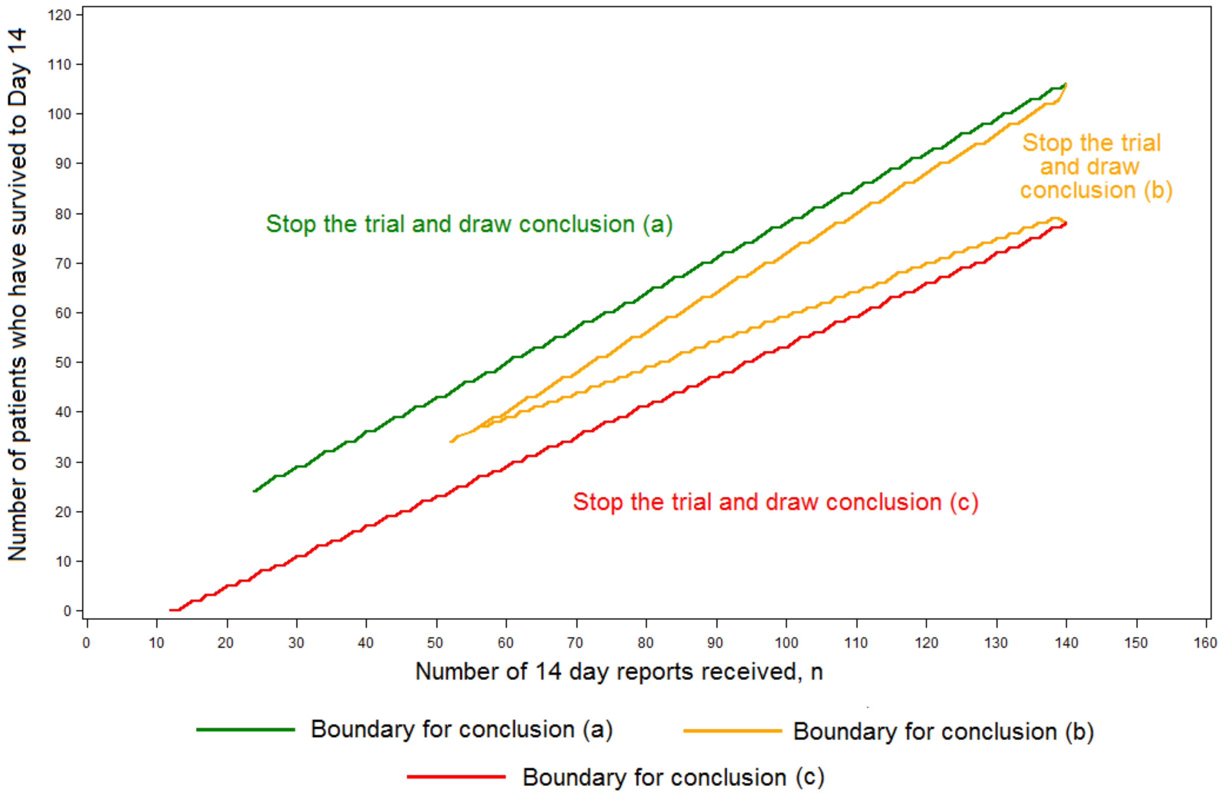


The properties of the design have been calculated exactly, based on the independent Bernoulli distributions of each patient outcome. These are shown in **Table 3**. The specified probability of reaching the most appropriate conclusion when P = 0.500, 0.667 or 0.800 is exceeded in each case (it is not possible to match them exactly due to the discrete nature of the data). It can be seen from **Table 3** that the average (were many such trials to be repeated) and the median sample sizes are much less than the maximum sample size of 140, and even the 90$^{th}$ percentiles of sample size (the values before which the trial will be stopped with probability 90%) do not reach 140.

**Table 3**: Exact properties of the trial design

| P: true probability of surviving for 14 days* | probability of concluding: (a) | (b) | (c) | final sample size: average | median | 90$^{th}$ %ile |
|---|---|---|---|---|---|---|
| 0.414 | 0.000 | 0.002 | 0.998 | 37.7 | 35 | 58 |
| 0.500 | 0.000 | 0.100 | 0.900 | 60.5 | 56 | 97 |
| 0.667 | 0.034 | 0.950 | 0.016 | 70.0 | 65 | 96 |
| 0.800 | 0.908 | 0.092 | 0.000 | 68.7 | 65 | 105 |
| 0.850 | 0.997 | 0.003 | 0.000 | 49.4 | 45 | 72 |

* For each of the three guide values of P, a high probability is set for reaching the most appropriate conclusion.

A second plot will be maintained of the number of patients who have been recruited minus the number known so far to have died within 14 days (S*), against the number recruited. When records are finally completed for all of these patients, the number who have survived 14 days must be less than S*. The plot of S* will be compared with the lower boundary of Figure 1 and the trial will be stopped with conclusion (c) if it is crossed. Once the records are complete to 14 days, the main plot must also lie below the lower boundary: there is no uncertainty in this prediction. This safety plot would ensure a rapid reaction to a treatment that proves to be lethal in the short term.

### 11.4. Final Analysis

When the trial has been completed, a point estimate and a 95% confidence interval will be computed for p using the method of Jovic and Whitehead,[17] which allows for the sequential nature of the trial.

### 11.5. Inclusion in Analysis

It is important to note that the primary sequential analysis described uses only data from adult patients who were recruited more than 14 days previously. Although it may be known that some more recently recruited patients have already died, their data will not yet be used, to avoid bias due to information on death being available sooner than information on survival.

### 11.6 Analysis of the data on children

The stopping rule described in Section 11.3 will be applied to data from adult patients only. When the trial is concluded for adult patients, the recruitment of children will also be terminated. The success rate for children will be estimated, and a 95% confidence interval will be computed using the exact method of Clopper and Pearson.[18] The difference between the success rates of children and adults will be analysed using approximate methods based on the normal distribution. Secondary endpoints will be analysed separately for both children and adults. For the purpose of analysis children are defined as all patients who are both <50kg in weight and <18 years of age.

### 11.7 Procedure for Accounting for Missing, Unused, and Spurious Data.

While considerable effort will be made to ensure all patients are followed up at Day 14 and Day 30 (through return visits to clinic, phone, email or field-researcher visits), some patients may not be contactable. Leeway will have to be allowed for late reporting, but this should be limited: it may be that data on survival to Day 14 is not entered until Day 16 in order to avoid remaining bias due to late reporting of survival. However, if it turns out that a patient died on Day 15 or later, *then they are survivors* for the purpose of this study. If they walked away on Day 7, seemingly cured, it might be assumed that they survived to Day 14. Some judgment will necessarily be used here.

### 11.8 Rationale for statistical design choice

In the context of the current EVD outbreak, a simple, robust and rapid trial is essential. Mortality dominates all other patient endpoints. Most deaths occur within 14 days of diagnosis, and so survival (or not) to Day 14 represents the most relevant clinical endpoint. The situation at Ebola Treatment Centres is highly challenging, and so this study places minimal additional responsibilities on them.

We believe that if evidence of a survival rate exceeding 0.800 there is strong evidence that the treatment is effective. Evidence that the rate lies close to 0.667 would identify the treatment as being worthy of further study. Although the patients are likely to be a heterogeneous group, there is little opportunity to use stratification or covariate adjustment in this trial. That is because the target values of the probability of survival to 14 days, 0.500, 0.667 and 0.800, are assumed to be relevant to everyone. For stratification, different targets would be needed for each stratum.

A fully sequential design is proposed so that the conclusion of the trial can be made available as quickly as possible. If all treated [adult] patients die before Day 14, then only 12 patients will be required, and if all survive, then 24 will be sufficient. Rapid reporting of minimal information would appear to be feasible, and in keeping with the urgency of the situation. Until the trial is terminated, data managers need only compare the number of survivals observed so far with the stopping boundaries shown on Figure 2. A table of values will be provided for this purpose.

The approach described is related to the sequential medical plans of Bross[19] but his designs where centred on p = 0.50, rather than p = 0.67 as required here. It is also a form of double triangular test, as described by Whitehead and Whitehead and Todd.[20, 21] The latter paper summarises some implementations of the method. The analysis is based on an orderings approach introduced by Fairbanks and Madsen.[22] Exact calculations have been made for this particular design, using SAS, and this package will be used for the analysis.

## 12. DATA MANAGEMENT

### 12.1. Data Capture

Patient files will serve as source clinical and drug dosing data for the study. Due to infection control procedures, paper documents cannot be taken outside of the high-risk containment zone; therefore records may be dictated to a secondary document outside of the zone and the primary document incinerated. Where possible, electronic data entry will capture source data directly or source documents will be photographed/scanned and archived. Original data files from the site and research laboratories will provide source data for laboratory testing results. Data recorded by Médecins Sans Frontières as a part of the standard case investigation form will be used to support the classification of disease severity as well as for validation and comparison of study data.

Data will be entered to a central study database on MACRO software. Data management, reporting and storage within this trial will comply with the requirements of European Union data protection laws, ICH Good Clinical Practice and FDA 21 CFR Part 11.

### 12.2. Access to Data

Data sharing will be under the aegis of the Trial Steering Committee and will adhere to the principles of rapid, open access as outlined in the World Health Organisation report on Ethical considerations for use of unregistered Interventions for Ebolavirus disease.[23]

## 13. QUALITY ASSURANCE PROCEDURES

Governance of the trial will be through the Trial Steering Committee (TSC), the Trial Operation Group (TOG) and the Independent Data Monitoring Committee (IDMC).

- The TSC will be the primary decision making body, with membership from each partner institution, a senior representative from each participating country, and other independent experts. Members of the TSC are given in Section 1 (members may change if necessary). Decisions of the Committee will be by consensus. WHO will be part of the Committee on an ex-officio basis. Terms of reference for the TSC will set out the full details of the membership, operations and remit.
- The TOG will be led from the ISARIC Coordination Centre in Oxford and report to the TSC. This team will provide operational oversight and day-to-day management of the trial to ensure compliance to follow up and critical factors such a retention and data quality. This team will comprise those with responsibility for the operational activities and so include representation from each site, data management, trial operations, training and logistical support, laboratory sample processing and handling.
- The IDMC will be responsible for reviewing the data from the study. Membership will include individuals with statistical, clinical and trial expertise. An IDMC charter will fully set out the committee's remit, membership and full definition of their operational conduct in terms of reviewing efficacy and safety data and reporting their recommendations to the TSC.

The trial will be conducted in accordance with the current approved protocol, relevant regulations and standard operating procedures. Regular monitoring will be performed to ensure quality control. Data will be evaluated for compliance with the protocol and accuracy. Following written standard operating procedures, the monitors will verify that the clinical trial is conducted and data are generated, documented and reported in compliance with the protocol and the applicable regulatory requirements.

The MACRO software will be configured to automatically cross-validate across entire patient records according to the specific requirements. Data Clarification requests will be built in to the system. The data validation and quality processes will comply with the new FDA and EU recommendations for data level trial monitoring and therefore allow for data quality to be assured in real time as a mechanism for on site monitoring.

## 14. ETHICAL AND REGULATORY CONSIDERATIONS

### 14.1. Regulations, Guidelines and Approval

The Investigators will ensure that this study is conducted in compliance with the current revision of the Declaration of Helsinki and the applicable principles of International Committee on Harmonization Good Clinical Practice Guidelines. Regulatory approval to conduct the study and import investigational products will be obtained from national authorities. The protocol, informed consent form and investigators brochure will be submitted to the University of Liberia Pacific Institute for Research and Evaluation (PIRE) Research Ethics Committee and regulatory authorities in-country LMHRA, as well as the Médecins Sans Frontières Research Ethics Committee, the World Health Organisation is providing an independent review of protocol and the Oxford Tropical Research Ethics Committee for written approval.

The Chief Investigator will submit and, where necessary, obtain approval from the above parties for all substantial amendments to the original approved documents.

## 14.2. Considerations in Informed Consent

**STANDARD CARE UPON PRESENTATION:** Patients are triaged to Suspected and Probable wards upon presentation based on symptom and contact history. Blood is drawn for Ebolavirus PCR testing daily according to standard care. Results are reported back to the clinical staff, who then visit patients to discuss the results. When laboratory results positive for Ebolavirus are received from the laboratory, a senior medical staff and a social worker visit the patient to discuss the results. Clinical and social counseling are provided as the patient is moved to the Confirmed ward. Recruitment to the study will not interfere with the standard care process. Research staff will be available to support ETC workers for study related tasks and to ensure that standard care is not disrupted by study procedures.

**PROCESS FOR TAKING INFORMED CONSENT:** Written informed consent to participate will be required from all participants or their representative. The staff who undertake the discussion of test results will decide when the patient should be approached regarding the study. This discussion should occur as soon as possible after the availability of confirmed results, but at a time when the patient is emotionally stable and the staff feel that they are capable of considering the risks and benefits of participation.

All eligible patients should be offered the opportunity to participate in the study. When the clinical staff and/or social workers agree that an adult patient is mentally and physically fit to discuss and consider participation in the study, the patient or the parent/guardian of a patient under 18 years, will be approached. If an eligible patient has reduced consciousness or is unresponsive to discussion due to illness severity, an appropriate representative will be approached for consent if available. Appropriate representatives will be selected according to the standard Ebola Treatment Centre processes for determining who can make decisions on a patient's behalf. In the case of a child who has no parent/guardian or in the case of a patient who is unfit to consent and there are no appropriate representatives available proxy consent will be sought from an independent adult who has been previously nominated. This person could be a doctor who is not a member of the research team who has already agreed to undertake this role and has been fully briefed on the trial.

Consent will be requested by the local study staff or another suitable qualified and trained person authorised to do so by the Principal Investigator.

**ADULTS:** Adult patients for the purpose of consent are defined as ≥18 years of age. Adults who are deemed fit to discuss and consider participation by the medical and social staff will give consent independently. If an adult is not considered mentally and physically fit to give informed consent, consent will be sought from a close relative who attends the clinic or an appointed representative. When consent is obtained from a relative, the patient will be approached for consent if at any time during study participation their condition improves such that they can consider consent.

**CHILDREN:** Children are patients less than 18 years of age. Consent for children to participate in the study will be sought from the parents or guardians of the child or an appointed representative in the case of a child without a contactable parent/guardian. Study participation will be discussed at the discretion of the clinical and social staff with children of suitable physical and mental fitness as well as emotional maturity. When consent is obtained from a representative, a parent/guardian will be approached for consent if at any time during study participation the study staff are able to contact them.

**DISCUSSING CONSENT:** Participants or their representative will be presented with an informed consent form that explains the purpose, methods, risks and benefits of the study. The form will be available in relevant written local languages. If the patient or representative cannot read, the form will be read to them in the presence of a witness when available. If a consent form is not available in a language familiar

to the participant, staff will verbally translate the form into the language of the patient. Standardization of translation will be supported by the use of recorded verbal translations with appropriate language and explanations in local terms that will be used to train study staff. When possible, a second staff will be present during the informed consent discussion to ensure that translations are appropriate, the form is read accurately, that all of the patient's questions have been addressed and to act as witness when possible. This will be facilitated by the paired working system of the clinics, but may not always be possible due to human resource limitations and limited shift times.

**DOCUMENTING CONSENT:** Patients or their representative will be asked to sign and date an informed consent form prior to participation. Those unable to write will be asked to make a mark on the signature line only. If a witness was present during the consent process s/he will co-sign the consent form to confirm the accurate reading of the form and the consent of the participant/parent/guardian/representative. If consent for an adult patient is given by a representative, patients will be asked to sign a separate informed consent form when/if they are deemed physically and mentally fit at a later point during the study. When a second staff is present for the informed consent discussion, s/he will sign the informed consent form. A child's assent to participate will not be recorded.

**ARCHIVING OF INFORMED CONSENT FORMS:** Due to infection control procedures, paper cannot be transferred externally from the confirmed wards. Therefore informed consent forms will be photographed and archived electronically, then destroyed according to waste disposal procedures for contaminated substances. Patients will not be given a copy of the signed form as it is not permitted to leave the high-risk containment zone. Patients or their representatives will be offered an unsigned copy of the form to retain upon discharge.

### 14.3. Participant Confidentiality

The trial staff will ensure that the participants' anonymity is maintained. All study documents and samples will be labeled with an anonymous study code. Identifying information collected as a part of this study will remain confidential. For the purpose of ensuring data integrity and to facilitate quality assurance, study records will link to clinic files, which include patient's name and other identifying information. Participants' names will be recorded at the time of enrolment to allow for their identification at follow-up visits. However identifiable information will be linked to stored data or samples only by a protected Master List. This list will not be shared outside of the site study staff and no identifying information will be transferred between sites. All CRFs and samples will be labeled with a study identification number only and stored in suitable secure locations. Only persons who have signed the locally appropriate data protection training will have access to the password-protected computer where entered data is stored. After conclusion of the project, data will be removed from the computers and stored in a safe place. Any scientific publications or reports will not identify any patient by name or initials. When the research team reviews their notes, they are also bound by professional confidentiality. All study data will be stored in secure databases only accessible to study staff. Study sponsors and health authorities will be given controlled access for the purpose of audit when necessary.

### 14.4. Sample Management

Aliquots of EDTA blood-derived plasma samples will be stored initially at the site clinical diagnostic laboratory. Subsequently, aliquots will be shipped to national and/or international partner laboratories according to national and international regulations. Samples will be anonymised with a unique study

identifier prior to shipping and any patient identifiable data will have been removed. The Country Principal Investigator and Chief Investigator will have access to the enrolment log linking study identifiers with patient identifiers. Material transfer agreements and export/import licenses (where required) will be obtained, and international regulations on storage and shipping of hazardous samples will be followed. Sample custody will be maintained by the investigators and decisions regarding use and transfer of samples will be made by the TSC. Research samples will be stored indefinitely and approval from the sponsor and/or ethics committees, as appropriate, will be sought prior to destruction. The study investigators are responsible for biological deactivation and ensuring appropriate destruction of any residual materials.

## 14.5. Risks and Benefits

There are no approved treatments for EVD and the standard of care remains supportive care and treatment of complications. An analysis of data on 3343 confirmed and 667 probable EVD cases estimates a case fatality rate of 70.8% (95% confidence interval [CI], 69 to 73).[2] Unpublished data from MSF indicates that the survival rate in laboratory confirmed cased of Ebolavirus infection admitted to Gueckedou Ebola Treatment Centre is not likely to be more than 50% overall (see statistics section). BCV has demonstrated antiviral activity against EVD in vitro in independent testing performed by the Centre for Disease Control and National Institutes of Health; in vitro effects were demonstrated at concentrations that encompass the range of EC50 values observed for dsDNA viruses which have been effectively treated with BCV in humans and animal models, and which have been achieved in patients treated for dsDNA virus infection. For these reasons, the proposed study is considered a reasonable first step with regards to determining whether BCV has a potential benefit in patients with laboratory confirmed Ebolavirus infection.

BCV therapy has a number of potential risks.

BCV therapy has the potential for gastrointestinal (GI) side effects, specifically diarrhea; it is possible, however, that the GI side effects of BCV therapy will overlap with the GI effects of EVD. Alternatively, patients with severe EVD-related diarrhea may not absorb BCV as well as those who do not have diarrhea. However, in transplant patients with adenovirus enteritis with severe diarrhea, the treatment of adenovirus infection with BCV tended to stabilize or improve symptoms, rather than worsen them. In addition to surveillance for and reporting of all SUSARS, subjects enrolled in this study will be monitored specifically for GI signs and symptoms, especially diarrhea, as experience from Study CMX001-201 indicates that these events are likely associated with the administration of BCV after three or more weeks of therapy.

As described in the IB, BCV is considered a potential human carcinogen based on the findings from a 13-week toxicology study in rats, in which specific tumors (mainly adenocarcinomas) were observed in BCV-treated animals much earlier than would be expected over the course of a normal lifespan. Recommendations from the manufacturers of BCV specify that BCV is a potential carcinogen and should therefore not be administered for longer than 3 months unless the potential benefit justifies the potential risk to the patient. This study involves a maximum of 2 weeks of treatment and any carcinogenic risk associated with administration of BCV in this patient population is considered to be outweighed by the potential benefit of preventing the significant mortality associated with EVD.

As described in the IB, in embryo/fetal development studies, decreased fetal body weight and survival (increased resorptions) as well as morphological changes (external malformations and visceral and skeletal developmental variations) were associated with BCV therapy at doses of 1.5 and 4.5 mg/kg/day. To date, the fatality in foetus' of pregnant women with EVD is not known to be less than 100%. If the treatment showed potential for efficacy in humans, a reduction in maternal mortality and a potential reduction on

foetal mortality could outweigh any foetal risk associated with administration of BCV. However, given the current lack of data on the efficacy of BCV treatment in humans infected with Ebolavirus, the theoretical risk of embryotoxicity precludes the administration of BCV to pregnant women. This study is designed to determine whether or not BCV shows a chance of effectiveness against Ebolavirus. As maximum of 140 patients will be enrolled in this study, based on the WHO Maternal and Child Perinatal Health Profile for Liberia, approximately 3 pregnant women would be excluded from the study during the recruitment period. If the results of this study determine that BCV may be effective, justification for exposing foetus' to the risk of embryotoxicity by the inclusion of pregnant women in the follow-up study may exist.

BCV has never been used in patients under 2 months of age. As the potential for harm in newborns and the potential efficacy of BCV are both currently unknown, this study has excluded this population. If this trial determines that BCV has a chance of effectiveness against EVD, the risks of including this population in subsequent studies could be justified. Based on population statistics and data available from the previous months of patient care, approximately 1 child under 2 months may be excluded during the study recruitment period.

The risks of conducting this trial include the possibility that the operation of the Ebola Treatment Centre (ETC) where the trial is conducted is compromised by any of the following events: a.) the ETC is overwhelmed by suspected EVD cases referred or self-referring in the belief that an effective treatment is available at the ETC, b.) that the ETC security is breached by persons seeking access to the investigation product, c.) that the security and safety of the ETC and ETC staff is compromised if there is a perception that the investigational product or the clinical trial are harmful to individual or community interests.

Whilst these are significant risks they are not in themselves arguments for not conducting this trial, since these risks will exist for all ETCs conducted in this setting, although the risks may be most acute for the earliest trials conducted.

Mitigation of these risks will be achieved by the following activities: A) Community sensitization and participation in the run up to trial initiation. Experienced MSF health promotion and community outreach workers will conduct this activity. B) Study drug will stored off site in a locked metal cabinet within a secure building. C) Sufficient study drug for the current number of enrolled patients will be transported to the ETC daily. D) Contingency plans for handling an increase in the number of suspected cases will be developed, including strict criteria for closing to new admissions at the site conducting the study and the identification of satellite ETC centers to accept patients that cannot be acceptable at the ETC conducting the trial. E) evacuation plans will be in place in the event of a major security breach.

### 14.6. Expenses

Clinic and treatment costs are covered by Médecins Sans Frontières (and/or other organization/institution funding the site, including partner laboratories) for all patients presenting to the Ebola Treatment Centre regardless of study participation. The study drug will be supplied without cost to the patient. Patients will be issued a mobile phone for the purposes of follow-up. This phone and SIM card will become the property of the patient. Reasonable travel expenses and compensation for loss of earnings will be paid for attending the Day 14 and Day 30 follow-up at the Ebola Treatment Centre. No other funding or incentives for participation will be given to the patients.

### 14.7. Contemporaneous Studies

Patients cannot enroll to any other clinical trial that involves a therapeutic or care intervention while they are participating in this study. If participating sites are undertaking observational studies, patients may be

co-enrolled provided the combined study procedures are considered safe and appropriate for the patients and there is no conflict of outcomes or endpoints between the studies.

## 14.8. Alternatives to Study Participation

All patients will be treated with the best available care regardless of study participation. Patients are free to decline participation in this study without affect on the standard care provided.

Patients who are pregnant, less than 2 months of age, and/or unable to swallow study treatment are not eligible for study participation. These patients will receive treatment from the MSF clinical staff based on the current MSF guidelines for management of EVD. This includes appropriate rehydration, anti-infectives and symptom control.

## 14.9. Community Engagement

Médecins Sans Frontières (MSF) runs comprehensive Health Promotion activities alongside all Ebola Treatment Centres. Standard interventions include dissemination of health promotion messages to health authorities and communities regarding MSF activities and information on EVD transmission, prevention, reporting and response. Information on this research study would be added to these messages.

Messages to the community are disseminated by networks of MSF Health Promotion workers who engage with local leaders that serve as primary sources of information for the communities. Radio broadcasting and printed information sheets and posters are also common tools. This standard protocol on dissemination of critical messages would be used to inform the communities about research done at the Ebola Treatment Centre. Information regarding the nature and purpose of the research would be distributed with a variety of tools across these networks.

Messages to health authorities are disseminated to District and County Health Officers by the MSF Medical Team Leader and Field Coordinator. Education on the current development status of EVD treatments, clinical research studies ongoing in West Africa and the scientific background and methods of the current project would be distributed to and discussed with the health authorities already engaged as a part of the Health Promotion activities.

MSF also provides psychological and social support to EVD patients. When presenting to the Ebola Treatment Centre, patients and families will be approached by a team social worker to facilitate the psychological process throughout admission, EVD confirmation, treatment and when required, death and bereavement. Patients who are discharged from the Treatment Centre will be supported in their return to the community. This team will be engaged in the research study according to the requirements of the sites to assist with explaining the nature and methods of the research, obtain informed consent, and discuss the study with the family.

## 14.10. Drug Access Post-Study

The study investigators and partners are in discussions with the producers of BCV to provide access to BCV upon completion of the study if it is demonstrated to be effective.

## 15. FINANCE AND INSURANCE

### 15.1. Funding

The trial is funded by the Wellcome Trust.

### 15.2. Insurance

The University of Oxford has arranged appropriate insurances to provide for the University's responsibilities, as Sponsor, to research subjects; and, to cover the legal liabilities of the University to those engaged by the University in the performance of this research. The University will also arrange, or arrange in conjunction with other participating partners those emergency medical repatriation facilities which can be achieved, subject to the exigencies of arranging such at the material time.

## 16. PUBLICATION POLICY

Results from the trial will be published in open access and the data will be available for sharing.

## 17. REFERENCES

1. WHO. Ebola Response Roadmap Situation Report 129 August 2014: Available from: http://www.who.int/csr/disease/ebola/situation-reports/29-august-2014.pdf?ua=1.

2. WHO Ebola Response Team. Ebola virus disease in West Africa--the first 9 months of the epidemic and forward projections. N Engl J Med. 2014;371:1481-95.

3. Kucharski AJ, Edmunds WJ. Case fatality rate for Ebola virus disease in west Africa. Lancet. 2014;384:1260.

4. Gire SK, Goba A, Andersen KG, Sealfon RS, Park DJ, Kanneh L, et al. Genomic surveillance elucidates Ebola virus origin and transmission during the 2014 outbreak. Science. 2014;345:1369-72.

5. Fowler RA, Fletcher T, Fischer WA, 2nd, Lamontagne F, Jacob S, Brett-Major D, et al. Caring for critically ill patients with ebola virus disease. Perspectives from west Africa. Am J Respir Crit Care Med. 2014;190:733-7.

6. Mahanty S, Bray M. Pathogenesis of filoviral haemorrhagic fevers. Lancet Infect Dis. 2004;4:487-98.

7. Takada A, Kawaoka Y. The pathogenesis of Ebola hemorrhagic fever. Trends Microbiol. 2001;9:506-11.

8. Bausch DG, Towner JS, Dowell SF, Kaducu F, Lukwiya M, Sanchez A, et al. Assessment of the risk of Ebola virus transmission from bodily fluids and fomites. J Infect Dis. 2007;196 Suppl 2:S142-7.

9. Towner JS, Rollin PE, Bausch DG, Sanchez A, Crary SM, Vincent M, et al. Rapid diagnosis of Ebola hemorrhagic fever by reverse transcription-PCR in an outbreak setting and assessment of patient viral load as a predictor of outcome. J Virol. 2004;78:4330-41.

10. Ebihara H, Rockx B, Marzi A, Feldmann F, Haddock E, Brining D, et al. Host response dynamics following lethal infection of rhesus macaques with Zaire ebolavirus. J Infect Dis. 2011;204 Suppl 3:S991-9.

11. Rollin PE, Bausch DG, Sanchez A. Blood chemistry measurements and D-Dimer levels associated with fatal and nonfatal outcomes in humans infected with Sudan Ebola virus. J Infect Dis. 2007;196 Suppl 2:S364-71.

12. Wauquier N, Becquart P, Padilla C, Baize S, Leroy EM. Human fatal zaire ebola virus infection is associated with an aberrant innate immunity and with massive lymphocyte apoptosis. PLoS Negl Trop Dis. 2010;4:

13. Oestereich L, Ludtke A, Wurr S, Rieger T, Munoz-Fontela C, Gunther S. Successful treatment of advanced Ebola virus infection with T-705 (favipiravir) in a small animal model. Antiviral Res. 2014;105:17-21.

14. Qiu X, Wong G, Audet J, Bello A, Fernando L, Alimonti JB, et al. Reversion of advanced Ebola virus disease in nonhuman primates with ZMapp. Nature. 2014;
15. Warren TK, Wells J, Panchal RG, Stuthman KS, Garza NL, Van Tongeren SA, et al. Protection against filovirus diseases by a novel broad-spectrum nucleoside analogue BCX4430. Nature. 2014;508:402-5.
16. Sayburn A. WHO gives go ahead for experimental treatments to be used in Ebola outbreak. BMJ. 2014;349:g5161.
17. Jovic G, Whitehead J. An exact method for analysis following a two-stage phase II cancer clinical trial. Stat Med. 2010;29:3118-25.
18. Clopper CJ, Pearson ES. The use of confidence or fiducial limits illustrated in the case of the binomial. Biometrika. 1934;26:404-13.
19. Bross IJ, Delaney MM. Significance tests for certain 2 x 2 tables. Am J Hyg. 1952;55:357-62.
20. Whitehead J. The Design and Analysis of Sequential Clinical Trials. Revised 2nd edition ed. Chichester: Wiley; 1997.
21. Whitehead J, Todd S. The double triangular test in practice. Pharmaceutical Statistics. 2004;3:39-49.
22. Fairbanks K, Madsen R. P values for tests using a repeated significance test design. Biometrika. 1982;69:69-74.
23. WHO. Ethical considerations for use of unregistered interventions for Ebola viral disease. Report of an advisory panel to WHO. Geneva, Switzerland: WHO; 2014 [9th September 2014]; Available from: http://apps.who.int/iris/bitstream/10665/130997/1/WHO_HIS_KER_GHE_14.1_eng.pdf?ua=1.

## 18. APPENDIX A: TRIAL FLOW CHART

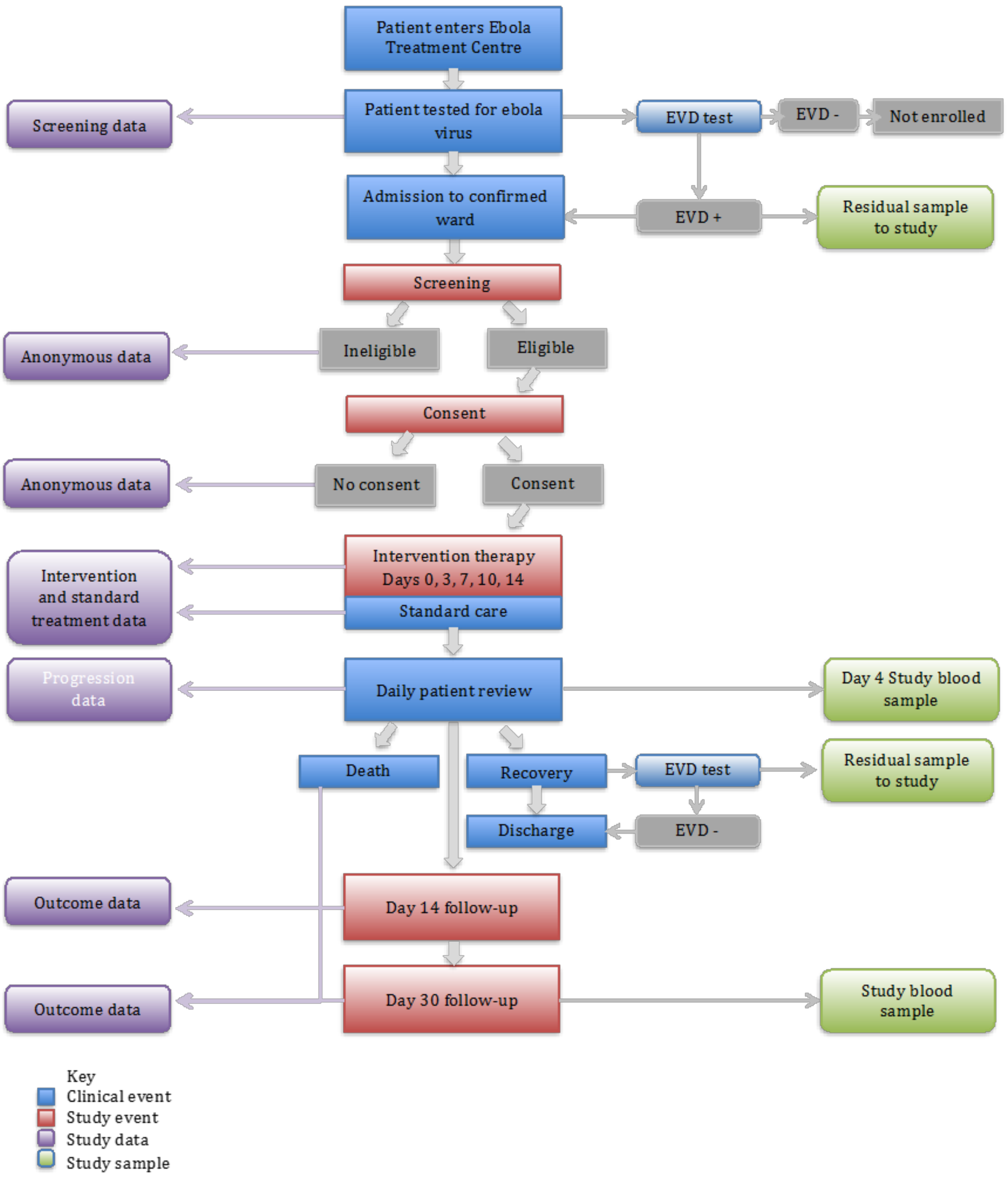

## 19. APPENDIX B: DOSING VOLUMES FOR DISOLVED TABLETS

Patients weighing less than 50 kg should have tablets dissolved in a closed container with 20 mL of water, shaken vigorously for 30 seconds and the appropriate volume drawn up into a graduated syringe. The table below shows the number of pills and volume of solution appropriate for each dose and each weight. Doses should be rounded up to the nearest mL.

- Initial dose of 4mg/kg = **2 tablets dissolved in 20 mL water**

followed by

- Four doses of 2mg/kg =**1 tablet dissolved in 20 mL water**

| Patient weight (kg) | Dose volume | Rounded dose volume (mL) |
|---|---|---|
| 3 | **1.2** | **2** |
| 4 | **1.6** | **2** |
| 5 | **2** | **2** |
| 6 | **2.4** | **3** |
| 7 | **2.8** | **3** |
| 8 | **3.2** | **4** |
| 9 | **3.6** | **4** |
| 10 | **4** | **4** |
| 11 | **4.4** | **5** |
| 12 | **4.8** | **5** |
| 13 | **5.2** | **6** |
| 14 | **5.6** | **6** |
| 15 | **6** | **6** |
| 16 | **6.4** | **7** |
| 17 | **6.8** | **7** |
| 18 | **7.2** | **8** |
| 19 | **7.6** | **8** |
| 20 | **8** | **8** |
| 21 | **8.4** | **9** |
| 22 | **8.8** | **9** |
| 23 | **9.2** | **10** |
| 24 | **9.6** | **10** |
| 25 | **10** | **10** |

| Patient weight (kg) | Dose volume | Rounded dose volume (mL) |
|---|---|---|
| 26 | **10.4** | **11** |
| 27 | **10.8** | **11** |
| 28 | **11.2** | **12** |
| 29 | **11.6** | **12** |
| 30 | **12** | **12** |
| 31 | **12.4** | **13** |
| 32 | **12.8** | **13** |
| 33 | **13.2** | **14** |
| 34 | **13.6** | **14** |
| 35 | **14** | **14** |
| 36 | **14.4** | **15** |
| 37 | **14.8** | **15** |
| 38 | **15.2** | **16** |
| 39 | **15.6** | **16** |
| 40 | **16** | **16** |
| 41 | **16.4** | **17** |
| 42 | **16.8** | **17** |
| 43 | **17.2** | **18** |
| 44 | **17.6** | **18** |
| 45 | **18** | **18** |
| 46 | **18.4** | **19** |
| 47 | **18.8** | **19** |
| 48-49 | **19.2** | **20** |

## 20. APPENDIX C: AMENDMENT HISTORY

| Amendment No. | Protocol Version No. | Date issued | Author(s) of changes | Details of Changes made |
|---|---|---|---|---|
| | | | | |

## RAPIDE BCV Protocol Amendment History

| Protocol Version | Date issued | Approved by | Author(s) of changes | Details of Changes made |
|---|---|---|---|---|
| **v2.1** | **05NOV2014** | Oxford Tropical Research Ethics Committee (OxTREC) approval day 13NOV2014 | PI & Co-Investigators | This was the first protocol approved by OxTREC, the first protocol approved in Liberia and Médecins Sans Frontières was v2.8 01DEC2014 |
| **v2.8** | **01DEC2014** | Liberia PIRE-IRB approval day 05DEC2014<br><br>OxTREC approval day 19DEC2014<br><br>Ethics Review Board - Médecins Sans Frontières (ERB-MSF) approval day 19DEC2015 | PI & Co-Investigators<br><br>changes were recommended by the Liberia IRB and by MSF ERC | **Protocol v2.1 to v2.8 01DEC2014**<br>• Change in Co-Investigators<br>• Registration number with PACTR<br>• Change IDMC members<br>• Dosing: specification for dissolving IMP; change in dosing days of administration<br>• Schedule of study procedures up dated<br>• Inform consent: clarification of consent for unaccompanied minors and proxy consent sections 8.2 and 14.2<br>• Lab assessment: specification of standard of care<br>• Supportive therapy: further details were added<br>• Repeated section 11.1 deleted<br>• Clarification on ethics committees that approved protocol<br>• Risk and benefits: further details on side effects, carcinogen and risk to foetus due to embriotoxicity<br>• Clarification on alternatives to study participation<br>• Trial flow chart updated<br><br>Other documents<br>• Child proxy ICF added |
| **v2.8** | **01DEC2014** | OxTREC approval day 19DEC2014<br><br>Liberia PIRE-IRB approval 26DEC2014 | PI and Co-investigators | No change in protocol<br>Modification in ICF and CRF only:<br>• RAPIDE CVF ICF Child Proxy v1.6 18DEC2014;<br>• RAPIDE BCV ICF Child v2.2 18DEC2014;<br>• RAPIDE ICF Adult v2.2 18DEC2014; |

| | | | | |
|---|---|---|---|---|
| | | | | • RAPIDE CRF 18DEC2014 |
| **V2.8** | **01DEC2014** | OxTREC approval day 08JAN2015 | Non-substantial Amendment submitted 31DEC2014<br><br>PI & Co-investigators | Clarification Table 1 and Section 8.5 Laboratory Analysis:<br>Day 4 and Day 30 assays will be performed and residual samples stored only when an appropriate laboratory is in place and provide suitable storage facilities. A pregnancy test will still be performed on urine on Day 30 |

# General Investigational Plan

**Clinical Trial Title: Rapid Assessment of Potential Interventions & Drugs for Ebola (RAPIDE) - BCV**

| | |
|---|---|
| **Chief Investigator:** | Dr Peter Horby , Centre for Tropical Medicine and Global Health, University of Oxford |
| **Country Principal Investigator:** | Dr Stephen Kennedy, Univeristy of Liberia, PIRE |
| **Sponsor:** | University of Oxford |
| **Funder:** | Wellcome Trust |

*2nd `December 2014*

## 1. Background

Whilst several therapeutic interventions have shown promise in the laboratory and in animal studies, none have been tested for efficacy and safety in humans with EVD. A World Health Organization (WHO) expert panel recently concluded unanimously that "investigators have a moral duty to evaluate these interventions in the best possible clinical studies that can be conducted under the circumstances of the epidemic."

It is the intention to build a platform for investigation of a number of experimental interventions for the treatment of EVD. The strategy is therefore intended to find and assess a very effective treatment, if one exists, as quickly as possible, and in time to help the patients in the current outbreak.

This document provides the detailed justification for the proposed programme of research to rapidly evaluate investigational products for efficacy against Ebola virus disease.

The first study is of brincidofovir, as described in the study protocol. This is the first single-arm triage study and will be followed by additional studies that will be designed based on the results of this first trial and the availability of investigational products.

## 2. Overview

The flow chart below shows the potential stages in the assessment of a single experimental treatment in combination with best supportive care (Treatment A). In the initial phase II assessment, all trial patients receive Treatment A, and the primary endpoint is survival to Day 14 after entry. The trial will distinguish between three situations:

(a) Treatment A is very effective (about 67% of patients survive to Day 14);
(b) Treatment A is promising (about 50% of patients survive to Day 14); and
(c) Treatment A is not very effective (about 33% of patients survive to Day 14).

If conclusion (a) is reached, then Treatment A will be made generally available for the treatment of ebola, while a single-arm confirmatory study takes place. If this study fails to confirm the efficacy of Treatment A, then it will be evaluated further in a randomised phase III trial comparing it with a control treatment comprising best supportive care.

If conclusion (b) is reached, then Treatment A will be evaluated further in a randomised phase III trial comparing it with control.

If conclusion (c) is reached, then Treatment A will be set aside, although it may be revisited if no better treatments are found.

This scheme is illustrated in the flow chart shown in Figure 1.

The motivation for this approach is the urgency of identifying very effective treatments very quickly if they exist, while allowing the opportunity to find moderately effective treatments if there are none that are very effective. The value of 0.33 is based on WHO Ebola Response Team (2014) where a survival rate of 30% is quoted. Survival to Day 14 will be slightly more common than this.

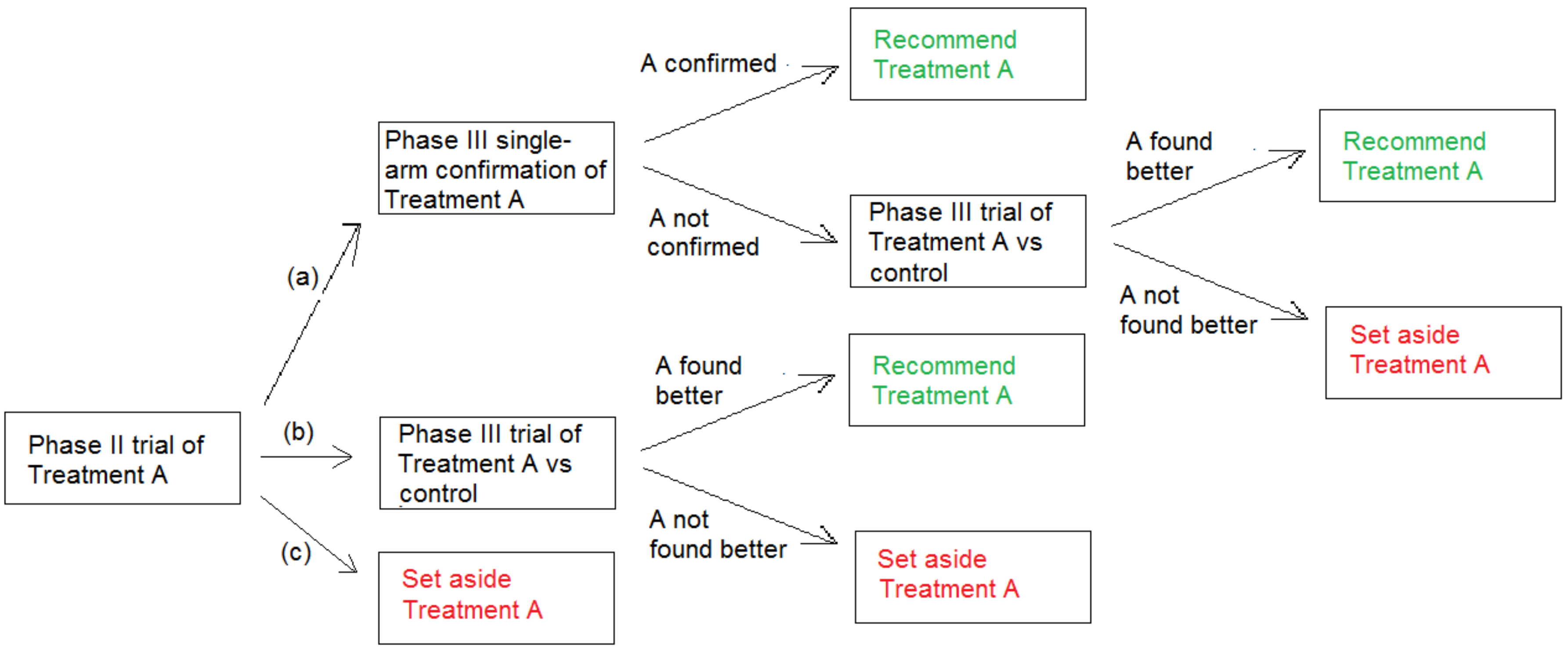


*Figure 1: Flow chart to show how a single experimental treatment can be evaluated*

## 2. The Phase II design

The phase II trial of Treatment A is designed to achieve (conservatively) the probabilities of reaching conclusions (a), (b) and (c) shown in Table 1.

**Table 1: Target error probabilities for the phase II design**

| True probability of surviving for 14 days | Probability of reaching conclusion: (a) | (b) | (c) |
|---|---|---|---|
| 0.333 | 0.000 | 0.100 | 0.900 |
| 0.500 | 0.025 | 0.950 | 0.025 |
| 0.667 | 0.900 | 0.100 | 0.000 |

A fixed sample trial that achieves these error probabilities would recruit 92 patients. Conclusion (a) would be reached if $S \geq 56$, where S is the number of patients who survive to Day 14. Conclusion (c) would be reached if $S \leq 36$ and conclusion (b) if $36 < S < 56$. With this design, the exact probability of reaching conclusion (c) when $p = 0.333$ is 0.9003, of reaching conclusion (b) when $p = 0.500$ is 0.9530, and of reaching conclusion (a) when $p = 0.667$ is 0.9003.

The sequential design to be used for phase II is shown in Figure 2. The intercepts of the lines that form the boundaries are at ±7.11721. The slopes of the four boundaries, working from the top to the bottom, are 0.55062, 0.65585, 0.34416 and 0.44939. A look-up table of boundary points is given as Appendix 1.

The trial would stop with conclusion (a) if all of the first 16 patients recruited survived to Day 14. The middle boundaries can reached from $n = 46$, where 23 survivals are required to reach it. Because of rounding, the middle boundary cannot be reached when $n = 47$. If none of the first 16 patients recruited survived to Day 14, then the trial would be stopped with conclusion (c). The boundaries are truncated at $n = 120$: if no boundary has been reached at the point, conclusion (b) is drawn. The method is similar to the sequential medical plans of Bross (1952), and is also related to the double triangular test (Whitehead and Todd, 2004).

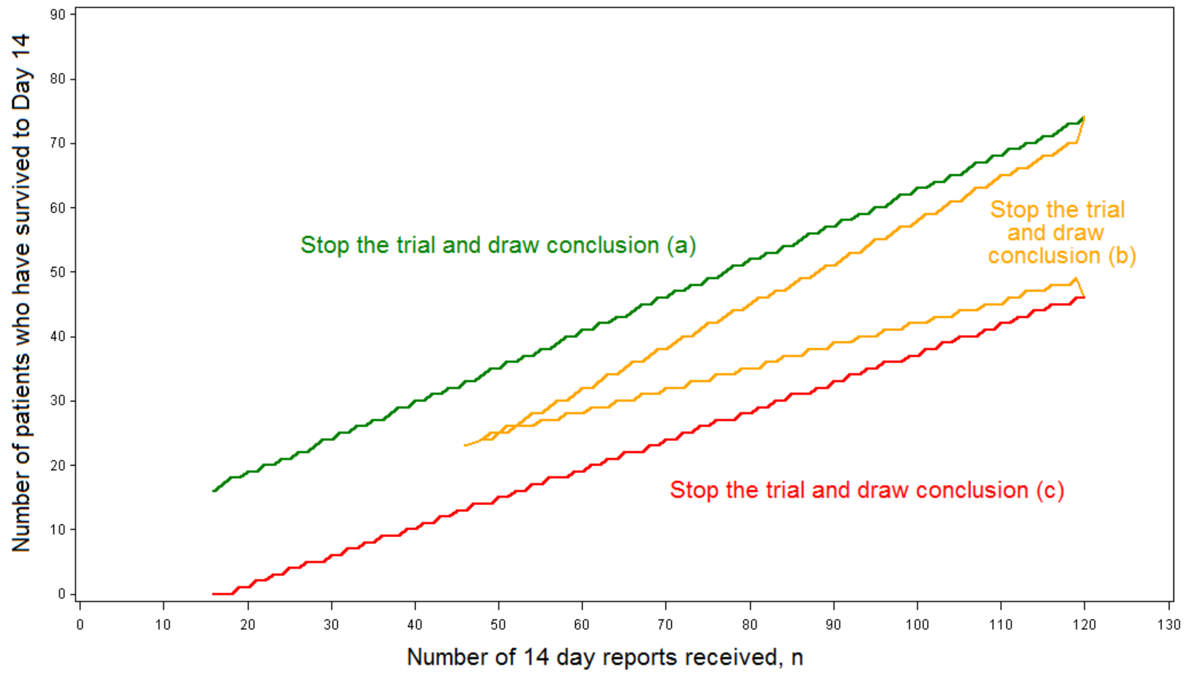


*Figure 2: Stopping boundaries for the phase II trial*

Table 2 shows the actual error probabilities achieved by this design. They are close to, but not precisely the same as those specified in Table 1. The values for each of the probabilities of reaching each of the conclusions (a), (b) and (c) when they are true slightly exceed the targets set.

**Table 2: Actual error probabilities for the phase II design**

| True probability of surviving for 14 days | Probability of reaching conclusion: (a) | (b) | (c) |
|---|---|---|---|
| 0.333 | 0.00000 | 0.09449 | 0.90551 |
| 0.500 | 0.02461 | 0.95078 | 0.02461 |
| 0.667 | 0.90551 | 0.09449 | 0.00000 |

The probabilities of reaching each possible conclusion and the properties of the final sample size are shown in Figures 3 and 4.

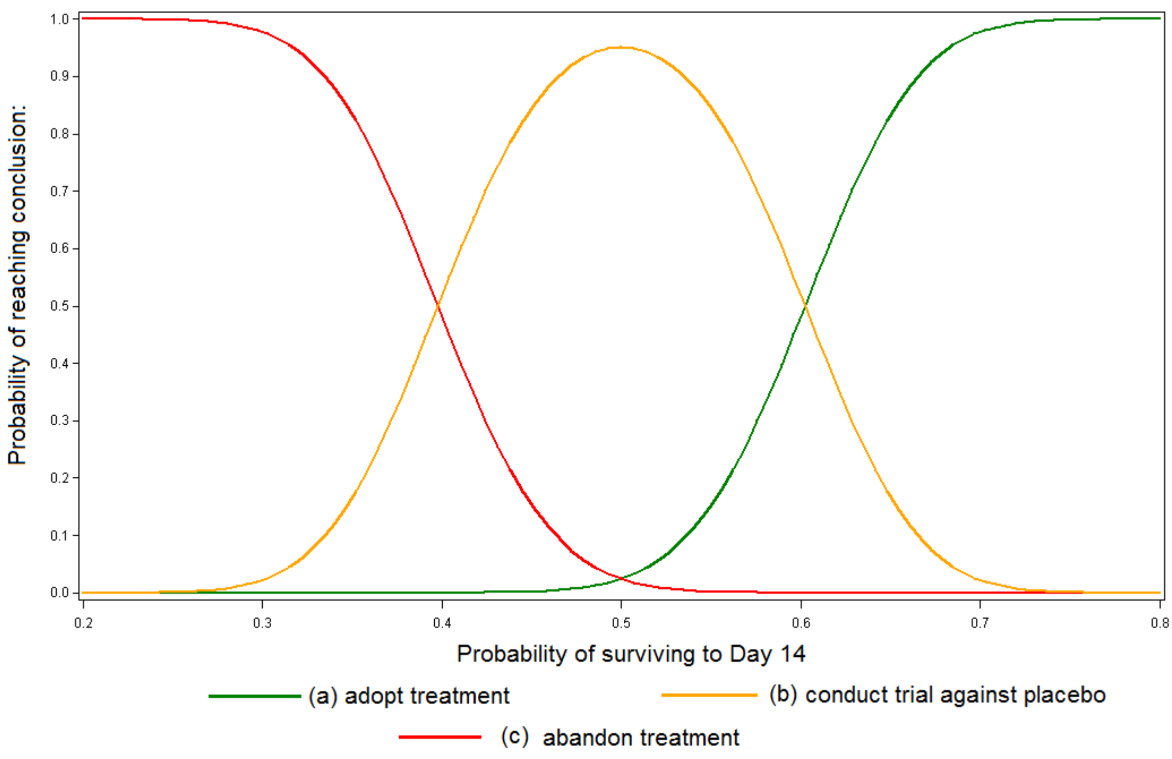


*Figure 3: Probabilities of reaching each conclusion for the phase II trial*

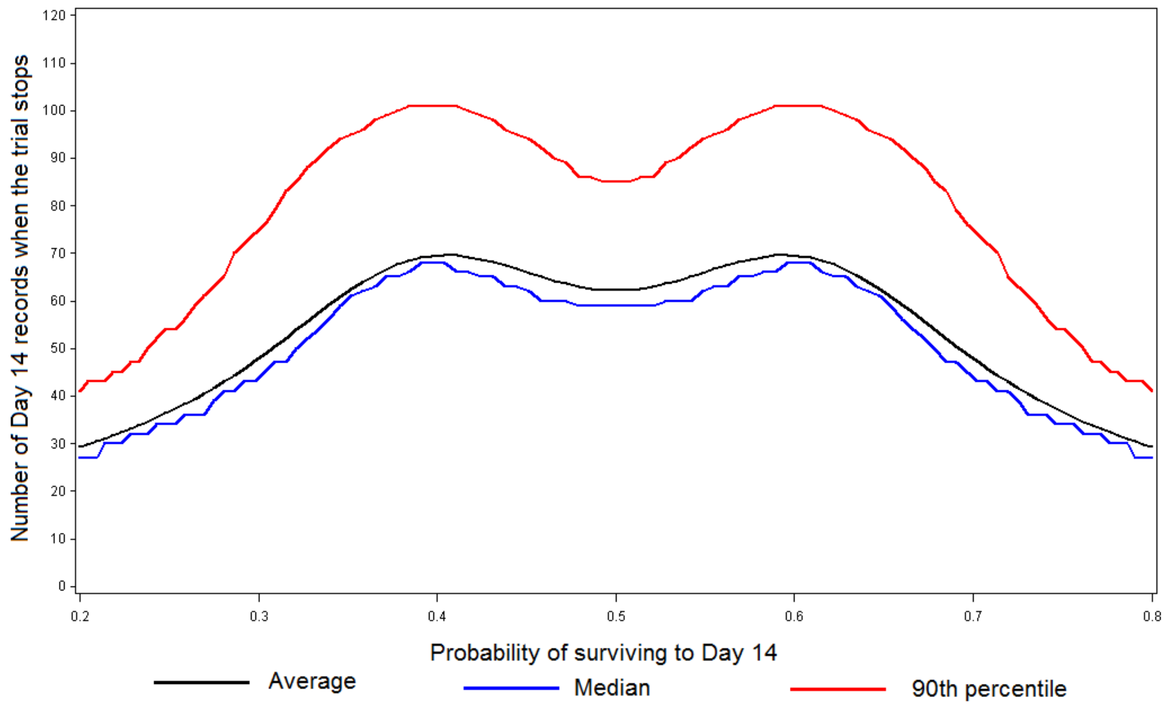


*Figure 4: Properties of the final sample size for the phase II trial*

Properties for some key values of p are shown in Table 3.

**Table 3: Probabilities of each conclusion and properties of the final sample size for the phase II trial**

| True probability of surviving for 14 days | probability of concluding: (a) | (b) | (c) | final sample size: average | median | $90^{th}$ %ile |
|---|---|---|---|---|---|---|
| 0.200 | 0.000 | 0.000 | 1.000 | 29.4 | 27 | 41 |
| 0.333 | 0.000 | 0.094 | 0.906 | 57.4 | 54 | 90 |
| 0.500 | 0.025 | 0.951 | 0.025 | 62.3 | 59 | 85 |
| 0.667 | 0.906 | 0.094 | 0.000 | 57.4 | 54 | 90 |
| 0.800 | 1.000 | 0.000 | 0.000 | 29.4 | 27 | 41 |

## 3. Design for the phase III single-arm confirmatory trial

The phase III confirmatory trial of Treatment A is designed to confirm that it is very effective with probability 0.900 if p = 0.667 and to wrongly confirm the treatment with probability 0.025 if p = 0.500. It will recruit a maximum of 108 patients, and will stop to set aside the experimental treatment if it is ever observed that $S \leq -5.2425 + 0.6300n$ where n is the number of patients who have been followed up for 14 days or more and S is the number who survived to Day 14. If the trial fails to confirm that Treatment A is effective, then it will be evaluated further in a randomised comparison with control in order to ascertain whether it is at least moderately effective. The boundary is shown in Figure 5 below. Using this design, the exact probability of confirming that the treatment is very effective when p = 0.667 is 0.90367 and the exact probability of wrongly confirming that it is very effective when p = 0.500 is 0.02429. Appendix 2 gives details of how starting values are deduced from asymptotic theory for a search for a suitable slope and intercept. This method is based on the futility design of Whitehead and Matsushita (2003).

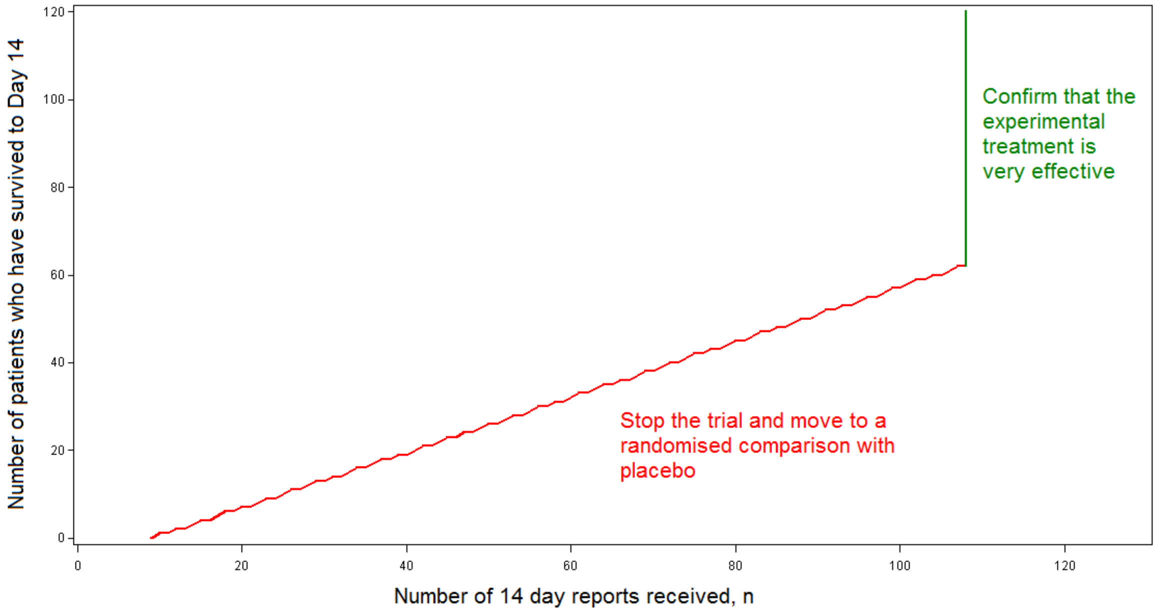


*Figure 5: Stopping boundaries for the single-arm confirmatory phase III trial*

Properties for some key values of p are shown in Table 4.

**Table 4: Probabilities of each conclusion and properties of the final sample size for the phase III single-arm confirmatory trial**

| True probability of surviving for 14 days | probability of confirming the treatment | final sample size: average | median | $90^{th}$ %ile |
|---|---|---|---|---|
| 0.200 | 0.000 | 12.9 | 12 | 17 |
| 0.333 | 0.000 | 18.7 | 17 | 28 |
| 0.500 | 0.024 | 41.9 | 36 | 75 |
| 0.667 | 0.904 | 103.1 | 108 | 108 |
| 0.800 | 1.000 | 108.0 | 108 | 108 |

The probabilities of failing to confirm that the experimental treatment is very effective and the properties of the final sample size are shown in Figures 6 and 7.

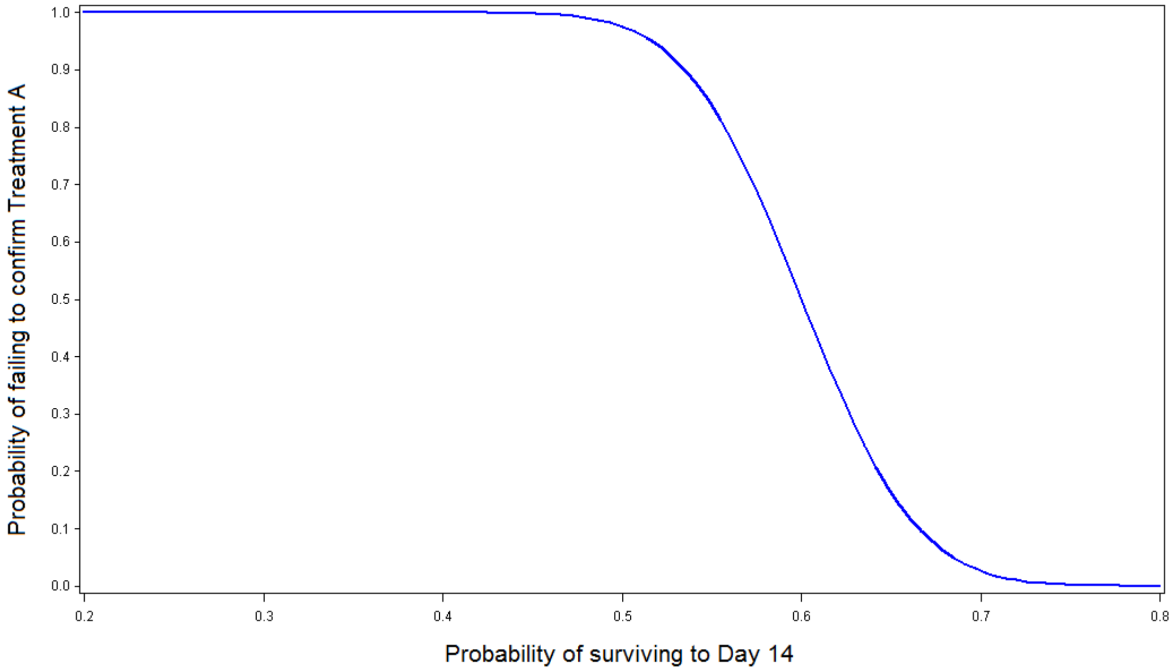


*Figure 6: Probabilities of failing to confirm the experimental treatment for the single-arm confirmatory phase III trial*

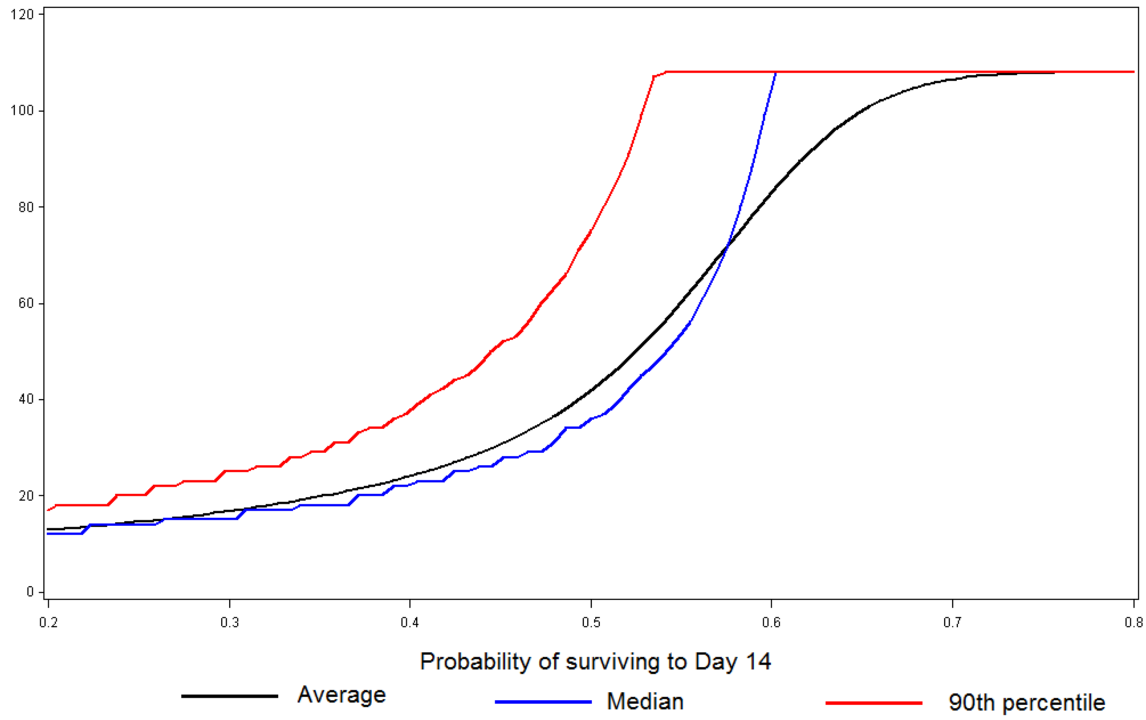


*Figure 7: Properties of the final sample size for the single-arm confirmatory phase III trial*

## 4. Design for the phase III comparison of Treatment A with control

### *4.1 Introduction to the trial design*

If the phase II trial ends with conclusion (b) that Treatment A is promising, then a randomised clinical comparison with standard care would be worthwhile. Patients would be randomised in a 1:1 ratio between standard care plus Treatment A and standard care alone.

At any stage in the trial, the data available from patients who have completed 14 days of follow-up can be summarised as shown in Table 5. Success means surviving to the end of Day 14 and failure means dying on or before Day 14. Only patients who were recruited to the study 14 or more days ago can be counted. Patients who were recruited within the last two weeks but have already died are not included, in this or any of the other designs discussed here.

**Table 5: Summary of data available for a randomised trial of Treatment A versus control**

| | Treatment A | Control | Total |
|---|---|---|---|
| Success | $S_A$ | $S_C$ | S |
| Failure | $F_A$ | $F_C$ | F |
| Total | $n_A$ | $n_C$ | n |

Because there are two counts of successes, the test statistics to be computed are more complicated than those used in single arm studies. Instead of plotting the number of successes on Treatment A against the sample size, we plot Z against V, where

$$Z = \frac{n_C S_A - n_A S_C}{n} \quad \text{and} \quad V = \frac{n_C n_A S F}{n^3}.$$

The statistic Z is a measure of the advantage of Treatment A over control. Notice that, if there are equal sample sizes in the two treatment groups ($n_A = n_C = n/2$), then $Z = (S_A - S_C)/2$. The statistic is tracking the excess of survivals on Treatment A over those on control, while allowing for any difference between the two sample sizes. If sample sizes are approximately equal, then

$$V \approx \frac{n}{4}\bar{p}(1-\bar{p})$$

provides an approximation to V, where $\bar{p}$ denotes the overall success rate in the trial. Thus, V is approximately proportional to sample size, while allowing for differences in the sample sizes and accounting for the observed success rate in the trial. Note that Pearson's chi-squared statistic, usually expressed as $\chi^2 = \sum(O-E)^2/E$, can be expressed as $\chi^2 = Z^2/V$, so these statistics are part of classic statistical analysis.

The trial is specified to have a power of 0.90 of reaching a significant conclusion that Treatment A has a better success rate than control at the one-sided level 0.025 if the odds ratio on success is 2. Denoting the success rates on Treatment A and placebo by $p_A$ and $p_P$ respectively, an odds ratio of 2 would occur if $p_A = \frac{1}{2}$ and $p_C = \frac{1}{3}$, or if $p_A = \frac{2}{3}$ and $p_C = \frac{1}{2}$, or if $p_A = \frac{4}{5}$ and $p_C = \frac{2}{3}$. The advantage of Treatment A over placebo will be summarised as the log-odds ratio, denoted by θ where

$$\theta = \log_e\left\{\frac{p_A(1-p_C)}{p_C(1-p_A)}\right\}.$$

For a log-odds ratio of 2, $\theta = \log_e(2) = 0.693$. The power is specified in terms of the log-odds ratio because of uncertainty over the value of $p_C$. If $p_C = \frac{1}{3}$, then there is 90% power to detect $p_A = \frac{1}{2}$, while If $p_C = \frac{1}{2}$, then there is 90% power to detect $p_A = \frac{2}{3}$, and so on. There is no need to know the value of $p_C$ in order to design and conduct the trial, and the power specification is guaranteed. However, predictions of how many patients will be recruited to the trial will depend on the values of $p_C$ and of $p_A$. Values will be assumed when making predictions, but these predictions will be wrong if these values are wrong.

### *4.2 A triangular design for the phase III comparative trial*

The design presented here is a triangular test, which is an asymmetric sequential procedure. Its asymmetry lies in it having high power to show that Treatment A is better than control if the odds ratio of success is equal to 2, but allowing much lower power to show that Treatment A

is worse than control if the odds ratio is equal to $\frac{1}{2}$. The trial will stop as soon as it is evident that Treatment A is better than control, or if it is apparent that such evidence of superiority will not be found. In the context of the ebola trial, there is no need to determine whether Treatment A is ineffective or whether it actually does harm. In either case, Treatment A should be abandoned.

Rather than updating the trial plot after every observation of a 14 day outcome, as recommended for the single arm studies, here it is suggested that up to 20 interim analyses of the data are conducted, occurring every time that 25 new 14 day records have been received (that is a total of 25 over both treatment arms). Thus the absolute maximum number of patients over both treatments will be 500. The calculations presented are approximate, unlike the exact results given for single-arm designs. This is because exact calculations are considerably more difficult to make in the two-arm case, and because the accuracy of approximate methods is much better when there are two treatment arms.

At each interim analysis, the statistics Z and V will be calculated. The trial will be stopped with the conclusion that Treatment A is significantly better than control if $Z \geq 6.39903 + 0.21049V$. The trial will be stopped with the conclusion that Treatment A is not significantly better than control if $Z \leq -6.39903 + 0.63148V$. Otherwise the trial will be continued until the next interim analysis. These stopping boundaries are illustrated in Figure 8. It is not essential that each new plotted point corresponds to results from exactly 25 new patients: the number may vary between 20 and 30. It is more important that the value of V increases by about 1.5 between each interim analysis: the target additional number of patients for each interim analysis may be varied once data are received to achieve this if the success rate in the trial as a whole turns out to be appreciably different from 0.583 (average of $\frac{1}{2}$ and $\frac{2}{3}$) as anticipated. The method is based on Whitehead (1997, 2011).

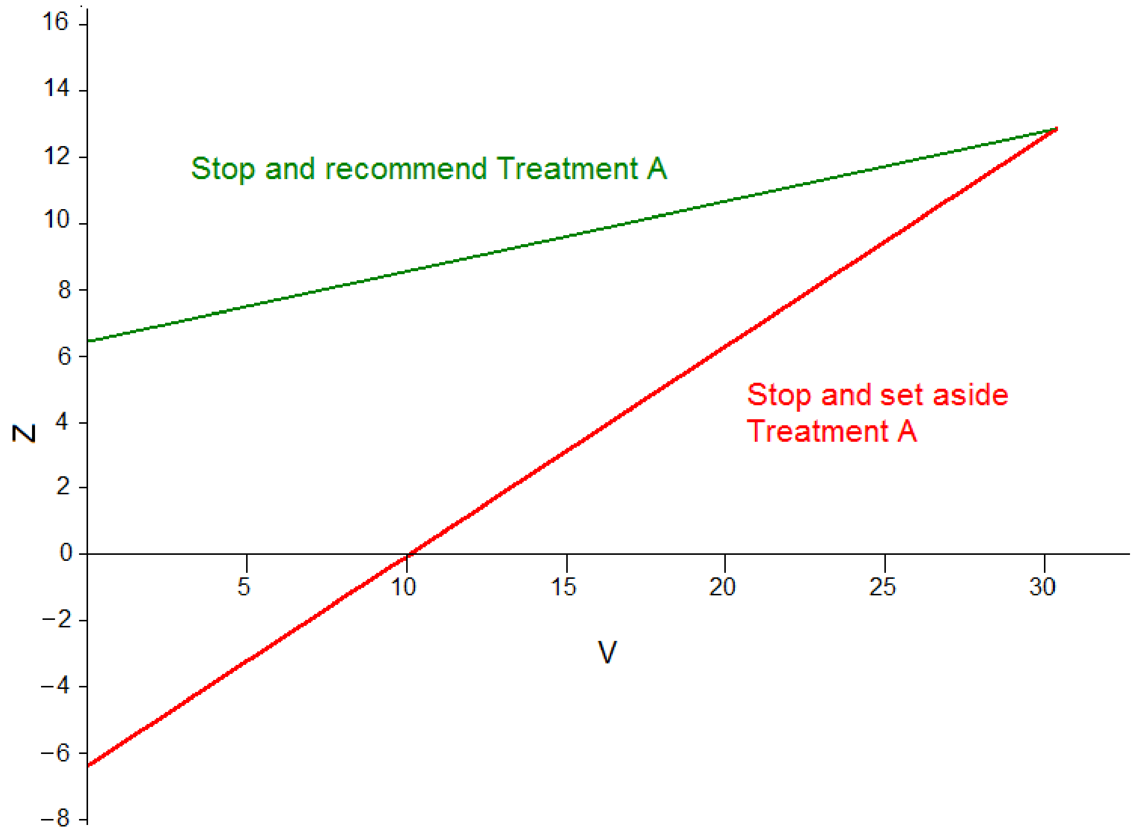


*Figure 8: Stopping boundaries for the phase III comparison of Treatment A with control*

Figures 9 and 10 respectively plot the probability that the upper boundary will be crossed and Treatment A recommended and the expected value of V against the true value of the log-odds ratio θ. Figure 11 plots the probabilities of stopping at or before the 2nd, 4th, 8th, 12th, 16th or 20th interim analysis against θ. These plots are not exact, but they are very accurate. However, they concern values of θ and V, which may be difficult to understand. Figures 12 and 13 are more approximate plots against the value of $p_A$ rather than θ, under the assumption that $p_C$ = 0.333, and the expected final value of n is presented rather than that of V. Figure 13 plots the probabilities of stopping at or before various sample sizes against $p_A$. It can be seen that the sample size is likely to be small when $p_A$ is less than $p_C$, or when it is considerably larger than $p_C$, with larger samples occurring if the advantage of Treatment A is modest.

For comparison, a fixed sample size trial of Treatment A against placebo would require V = 21.9 to achieve the same power requirement. This corresponds to a total sample size of about n = 360. It can be seen from Figure 12 that the expected value of n is smaller than this, even at its maximum.

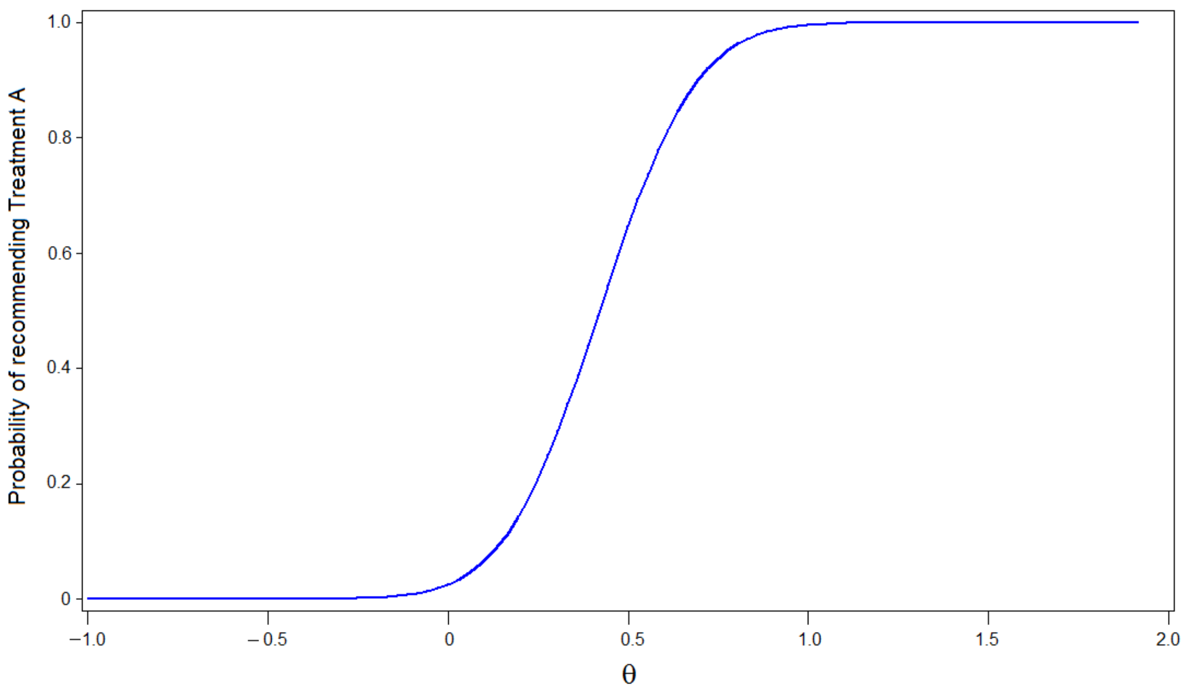


*Figure 9: The probability that Treatment A will be recommended for various true values of the log-odds ratio $\theta$ for the phase III comparison of Treatment A with control*

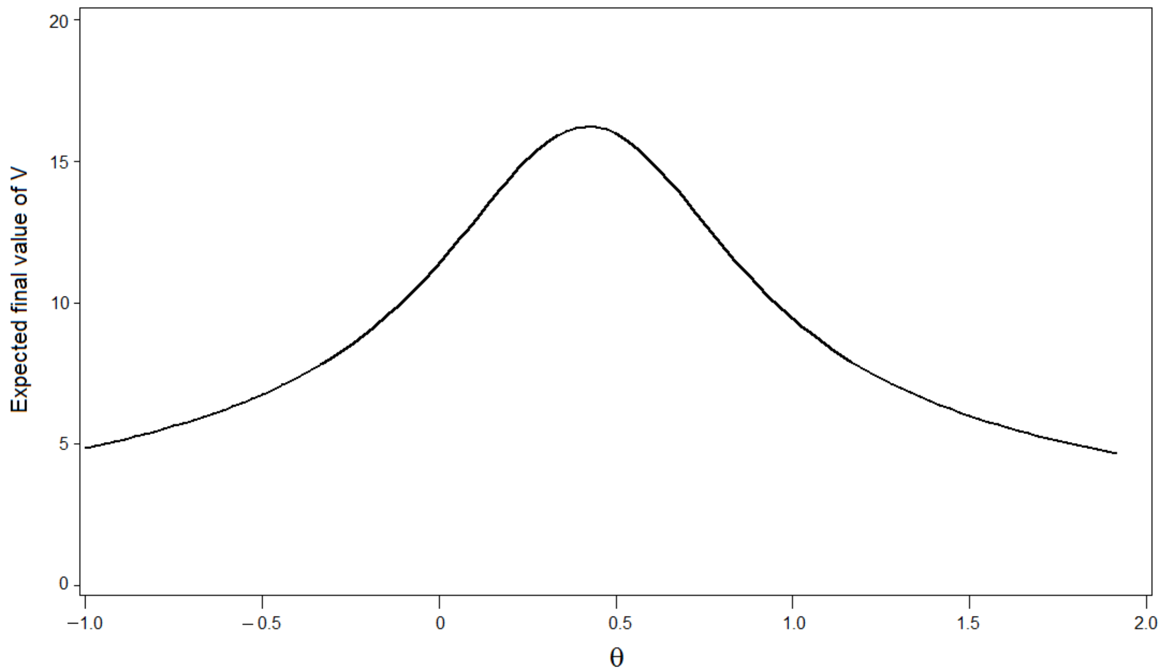


*Figure 10: The expected final value of V for various true values of the log-odds ratio $\theta$ for the phase III comparison of Treatment A with control*

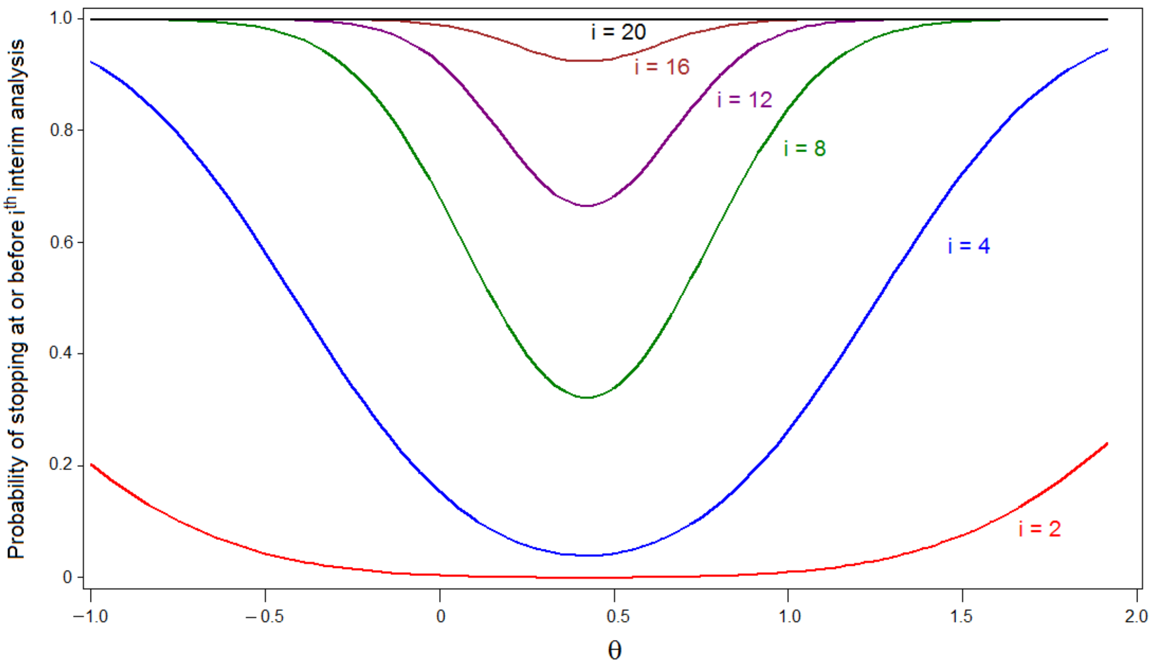


*Figure 11: Probabilities of stopping at or before the 2nd, 4th, 8th, 12th, 16th or 20th interim analysis for various true values of the log-odds ratio $\theta$ for the phase III comparison of Treatment A with control*

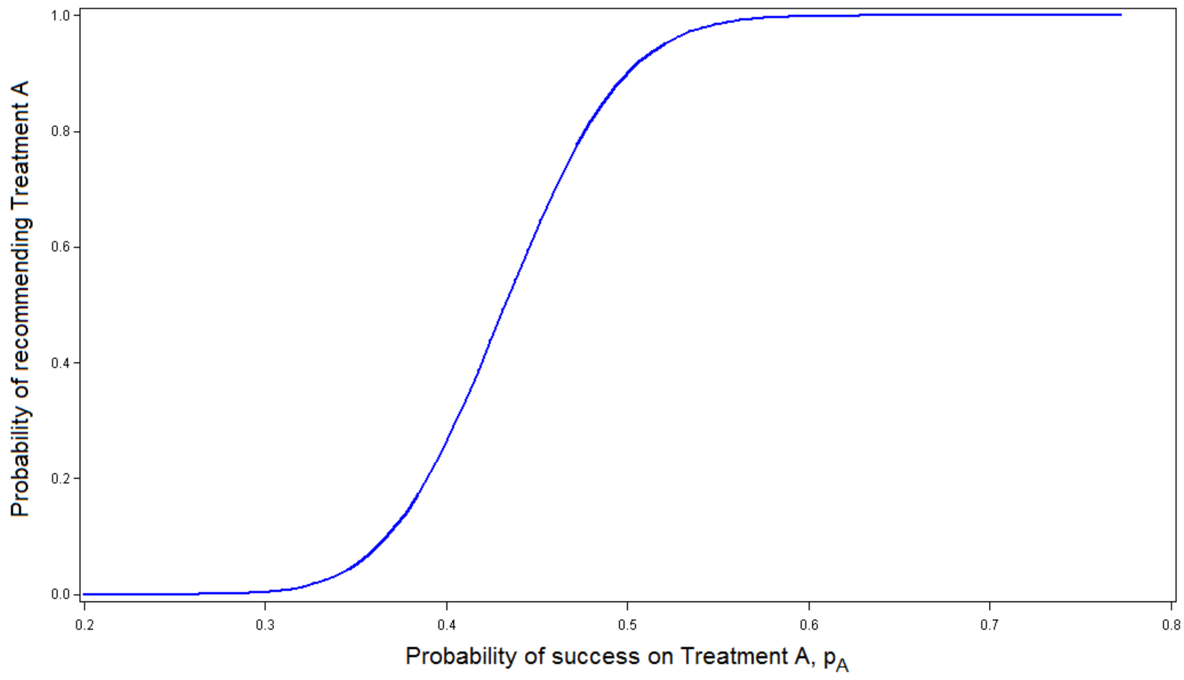


*Figure 12: The probability that Treatment A will be recommended for various true values of $p_A$, assuming that $p_C$ = 0.333, for the phase III comparison of Treatment A with control*

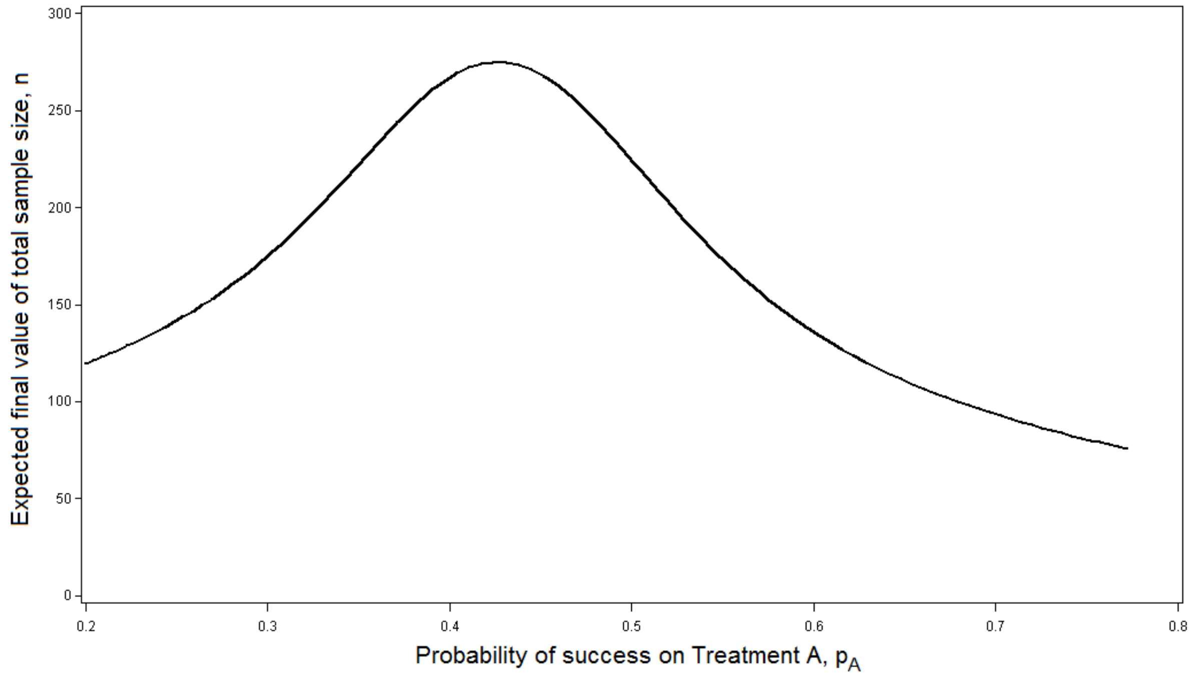


*Figure 13: The expected final value of total sample size, n, for various true values of $p_A$, assuming that $p_C = 0.333$, for the phase III comparison of Treatment A with control*

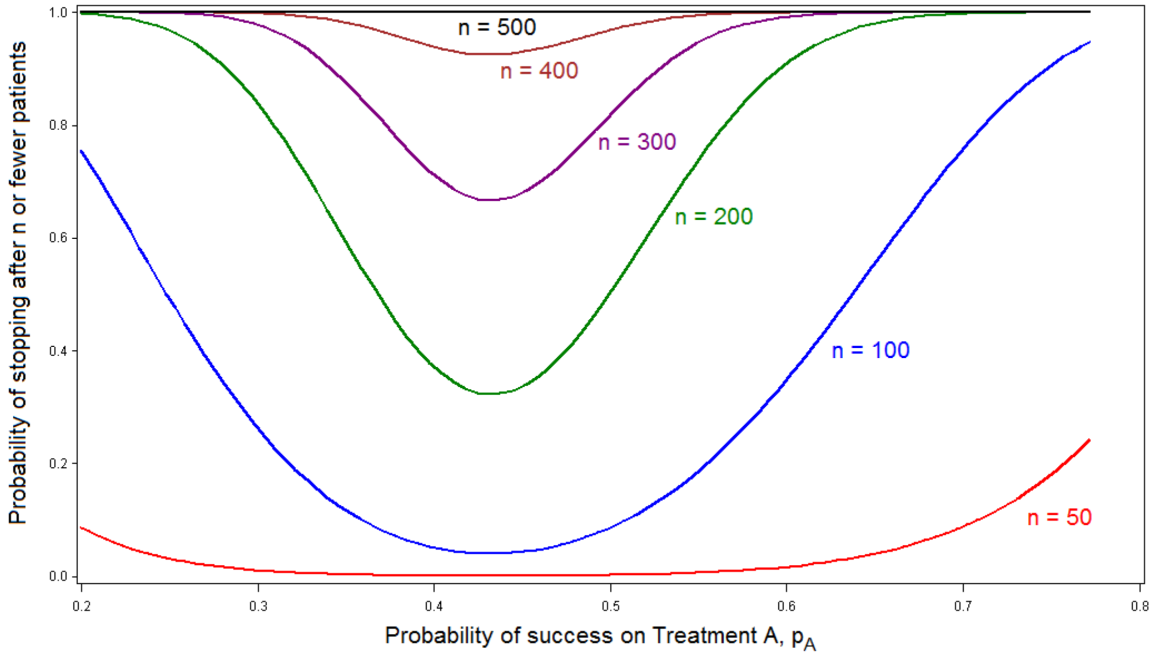


*Figure 14: Probabilities of stopping with no more than 50, 100, 200, 300, 400 or 500 patients for various true values of $p_A$, assuming that $p_C = 0.333$, for the phase III comparison of Treatment A with control*

Properties of the triangular design for some key combinations of $p_A$ and $p_C$ are shown in Table 6.

**Table 6: Probabilities of each conclusion and properties of the final sample size for the phase III comparison of Treatment A with control**

| $p_C$ | $p_A$ | θ | P(Rec A) | E(N*) | Probability of stopping with n ≤ | | | | |
|---|---|---|---|---|---|---|---|---|---|
| | | | | | 50 | 100 | 200 | 300 | 400 |
| 0.333 | 0.333 | 0.000 | 0.025 | 205.2 | 0.005 | 0.156 | 0.682 | 0.923 | 0.991 |
| | 0.500 | 0.693 | 0.900 | 224.6 | 0.002 | 0.087 | 0.504 | 0.819 | 0.969 |
| | 0.667 | 1.386 | 1.000 | 104.4 | 0.053 | 0.626 | 0.990 | 1.000 | 1.000 |
| 0.500 | 0.333 | −0.693 | 0.000 | 96.3 | 0.087 | 0.753 | 0.998 | 1.000 | 1.000 |
| | 0.500 | 0.000 | 0.025 | 182.4 | 0.005 | 0.156 | 0.682 | 0.923 | 0.991 |
| | 0.667 | 0.693 | 0.900 | 224.6 | 0.002 | 0.087 | 0.504 | 0.819 | 0.969 |
| | 0.800 | 1.386 | 1.000 | 114.7 | 0.053 | 0.626 | 0.990 | 1.000 | 1.000 |

## 5. Properties of the trial evaluation programme

There are six ways in which the trial programme could progress through the stages shown in Figure 1. These are shown in Table 7 below.

| Pathway | Phase II | Phase III | Extra Phase III | Final conclusion |
|---|---|---|---|---|
| 1 | (a) | Treatment A confirmed | - | **Recommend Treatment A** (very effective) |
| 2 | (a) | Treatment A not confirmed | A found better than control | **Recommend Treatment A** (moderately effective) |
| 3 | (a) | Treatment A not confirmed | A not found better than control | **Set aside Treatment A** |
| 4 | (b) | A found better than control | - | **Recommend Treatment A** (moderately effective) |
| 5 | (b) | A not found better than control | - | **Set aside Treatment A** |
| 6 | (c) | - | - | **Set aside Treatment A** |

**Table 7: Probabilities of each pathway for various combinations of $p_P$ and $p_A$**

| Pathway | $p_C$ | $p_A$ | Phase II | Phase III | Extra Phase III | Final conclusion |
|---|---|---|---|---|---|---|
| 1 | | | (a) | Confirm | - | **A wins** |
| | 0.333 | 0.333 | 0.000 | 0.000 | | 0.000 |
| | | 0.500 | 0.025 | 0.024 | | 0.001 |
| | | 0.667 | 0.906 | 0.904 | | 0.819 |
| | 0.500 | 0.333 | 0.000 | 0.000 | | 0.000 |
| | | 0.500 | 0.025 | 0.024 | | 0.001 |
| | | 0.667 | 0.906 | 0.904 | | 0.819 |
| | | 0.800 | 1.000 | 1.000 | | 1.000 |
| 2 | | | (a) | Not confirm | A beats P | **A wins** |
| | 0.333 | 0.333 | 0.000 | 1.000 | 0.025 | 0.000 |
| | | 0.500 | 0.025 | 0.976 | 0.900 | 0.022 |
| | | 0.667 | 0.906 | 0.096 | 1.000 | 0.087 |
| | 0.500 | 0.333 | 0.000 | 1.000 | 0.000 | 0.000 |
| | | 0.500 | 0.025 | 0.976 | 0.025 | 0.001 |
| | | 0.667 | 0.906 | 0.096 | 0.900 | 0.078 |
| | | 0.800 | 1.000 | 0.000 | 1.000 | 0.000 |
| 3 | | | (a) | Not confirm | A same as P | **A loses** |
| | 0.333 | 0.333 | 0.000 | 1.000 | 0.975 | 0.000 |
| | | 0.500 | 0.025 | 0.976 | 0.100 | 0.024 |
| | | 0.667 | 0.906 | 0.096 | 0.000 | 0.000 |
| | 0.500 | 0.333 | 0.000 | 1.000 | 1.000 | 0.000 |
| | | 0.500 | 0.025 | 0.976 | 0.975 | 0.024 |
| | | 0.667 | 0.906 | 0.096 | 0.100 | 0.009 |
| | | 0.800 | 1.000 | 0.000 | 0.000 | 0.000 |
| 4 | | | (b) | A beats P | - | **A wins** |
| | 0.333 | 0.333 | 0.094 | 0.025 | | 0.002 |
| | | 0.500 | 0.951 | 0.900 | | 0.856 |
| | | 0.667 | 0.094 | 1.000 | | 0.094 |
| | 0.500 | 0.333 | 0.094 | 0.000 | | 0.000 |
| | | 0.500 | 0.951 | 0.025 | | 0.024 |
| | | 0.667 | 0.094 | 0.900 | | 0.085 |
| | | 0.800 | 0.000 | 1.000 | | 0.000 |
| 5 | | | (b) | A same as P | - | **A loses** |
| | 0.333 | 0.333 | 0.094 | 0.975 | | 0.092 |
| | | 0.500 | 0.951 | 0.100 | | 0.095 |
| | | 0.667 | 0.094 | 0.000 | | 0.000 |
| | 0.500 | 0.333 | 0.094 | 1.000 | | 0.094 |
| | | 0.500 | 0.951 | 0.975 | | 0.927 |
| | | 0.667 | 0.094 | 0.100 | | 0.009 |
| | | 0.800 | 0.000 | 0.000 | | 0.000 |
| 6 | | | (c) | - | - | **A loses** |
| | 0.333 | 0.333 | 0.906 | | | 0.906 |
| | | 0.500 | 0.025 | | | 0.025 |
| | | 0.667 | 0.000 | | | 0.000 |
| | 0.500 | 0.333 | 0.906 | | | 0.906 |
| | | 0.500 | 0.025 | | | 0.025 |
| | | 0.667 | 0.000 | | | 0.000 |
| | | 0.800 | 0.000 | | | 0.000 |

**Table 8: Probabilities of recommending Treatment A for various combinations of $p_C$ and $p_A$**

| $p_C$ | $p_A$ | P(Rec A) |
|---|---|---|
| 0.333 | 0.333 | 0.002 |
| | 0.500 | 0.878 |
| | 0.667 | 0.913 |
| 0.500 | 0.333 | 0.000 |
| | 0.500 | 0.026 |
| | 0.667 | 0.982 |
| | 0.800 | 1.000 |

## 6. Final comments

All component trials would be analysed allowing for the sequential nature of the design used. This would lead to valid p-values and confidence intervals and to median unbiased estimates of the probability of survival past 14 days or of the log-odds ratio.

The scheme evaluated here is very idealised. Judgements will be made at the end of each component trial, and these might lead to modifications in successor trials. As more than one experimental treatment is to be evaluated in parallel, the phase III trials could be more complicated than indicated here. If more than one treatment is found to be very effective in Phase II, then they might be compared directly in randomised trials. If more than one treatment is found to be moderately effective in Phase II and none very effective,, then they might be compared with one another and with placebo in randomised trials.

## References


Bross, I. (1952). Sequential medical plans. *Biometrics* **8**, 188-205.

Clopper, C. J. And Pearson, E. S. (1934). The use of confidence or fiducial limits illustrated in the case of the binomial. *Biometrika* **26**, 404-413.

Fairbanks, K. and Madsen, R. (1982). P values for tests using a repeated significance test design. *Biometrika* **69**, 69-74.

Jovic, G. And Whitehead, J. (2010). An exact method for analysis following a two-stage phase II cancer clinical trial. *Statistics in Medicine* **29**, 3118-3125.

Whitehead, J. (1997). *The Design and Analysis of Sequential Clinical Trials*. (Revised 2nd Edition) Chichester: Wiley.

Whitehead, J. (2011). Group sequential trials revisited: simple implementation using SAS. *Statistical Methods in Medical Research* **20**, 636-656.

Whitehead, J. and Matsushita, T. (2003). Stopping clinical trials because of treatment ineffectiveness: a comparison of a *futility design* with a method of stochastic curtailment. *Statistics in Medicine* **22**, 677-687.

Whitehead, J. and Todd, S. (2004). The double triangular test in practice. *Pharmaceutical Statistics* **3**, 39-49.

WHO Ebola Response Team (2014). Ebola virus disease in West Africa – The first 9 months of the epidemic and forward projections. *New England Journal of Medicine* DOI 10.1056/NEJMoa1411100.
