# Supplementary material for: Experimental Treatment of Ebola Virus Disease with Brincidofovir
Source: PLoS One. 2016 Sep 9;11(9):e0162199. doi: 10.1371/journal.pone.0162199 (PMC5017617; doi:10.1371/journal.pone.0162199)
Supplement: S2 File — (DOCX) [file pone.0162199.s002.docx]

Supporting Information

S2 Text: Statistical Methods

Both children and adults were to be included in this trial. However, they were to be analyzed separately, as the effects of treatment may be different in the two groups. The primary analysis would concern the results from adults only, and a secondary analysis of the data in children would be used to ascertain whether the conclusions from those adult patients should apply to children. The stopping rules governing the study were based on data from adults only, and once met the whole study, in adults and children, would be stopped. In this section, sample sizes mentioned refer to the number of adult patients in the trial, and analyses refer to analyses of the data from adult patients only. For the purpose of analysis adults are defined as all patients ≥50kg in weight and/or ≥18 years of age.

Brincidofovir would first be evaluated in a phase II trial with a maximum of 140 patients without a concurrent control group, designed as a triage to classify the treatment as (a) very effective, (b) promising or (c) apparently ineffective. Denoting the true Day 14 survival rate of treated patients by p, if p = 0.800 then conclusion (a) was to be reached with probability 0.90; if p = 0.667 then conclusion (b) was to be reached with probability 0.95; and if p = 0.500 then conclusion (c) was to be reached with probability 0.90. The success rate threshold of a minimum of p = 0.500 was based on an analysis of a rudimentary data-set of 1820 sequential adult confirmed Ebola patients collected during July-October 2014 at four Médecins sans Frontières Ebola treatment units in Guinea, Sierra Leone and Liberia (including the study site used for this trial). Analyses of these data allowed an assessment of the Day 14 survival rate for patients treated with standard supportive therapy, which was estimated to be 0·43, with a 95% confidence interval of (0·40, 0·45), which was consistent with other contemporaneous assessments [1, 2]. The trial followed a sequential design, to be stopped according to a continuously maintained plot of the number of adult patients who had survived to Day 14 against the total number who had been recruited 14 days earlier. At the end of the trial, an exact analysis involving the estimation of p and associated 95% confidence interval was to be conducted according to an exact method of calculation. Children were not to be part of the sequential monitoring or the final analysis.

References

1. Team WHOER. Ebola virus disease in West Africa--the first 9 months of the epidemic and forward projections. N Engl J Med. 2014;371(16):1481-95. doi: 10.1056/NEJMoa1411100. PubMed PMID: 25244186; PubMed Central PMCID: PMC4235004.

2. Kucharski AJ, Edmunds WJ. Case fatality rate for Ebola virus disease in west Africa. Lancet. 2014;384(9950):1260. doi: 10.1016/S0140-6736(14)61706-2. PubMed PMID: 25260235.
