# Supplementary material for: Experimental Treatment of Ebola Virus Disease with Brincidofovir
Source: PLoS One. 2016 Sep 9;11(9):e0162199. doi: 10.1371/journal.pone.0162199 (PMC5017617; doi:10.1371/journal.pone.0162199)
Supplement: S3 File — (DOCX) [file pone.0162199.s003.docx]

Supporting information

S3 Text: Additional clinical details of trial participants

***Narrative of Participant 1***

Background

This 21 year-old female patient developed symptoms compatible with Ebola virus disease (EVD) and Ebola virus PCR testing was performed at a different healthcare facility where the patient initially presented. The patient was subsequently transferred to the trial centre following receipt of a positive EBOV PCR result at the initial facility. The patient was enrolled to the trial on the fourth day of illness.

Findings at enrolment

The patient was ambulatory and alert at the time of admission to the trial centre. Symptoms at enrolment included the following: fever; headache; fatigue; joint or muscle pains/aches; anorexia; nausea and vomiting (blood in vomitus); difficulty swallowing; chest pain; retro-orbital pain. The patient did not report diarrhoea at the time of enrolment, and it was noted that the patient was jaundiced. The patient had the following abnormal vital signs at enrolment: heart rate 104 bpm; respiratory rate 30 bpm; blood pressure 90/65 mmHg.

Ebola Treatment Unit (ETU) clinicians repeated EBOV PCR testing on admission to the trial centre and the cycle threshold (Ct) value was 22·70. A malaria rapid test performed on admission was negative.

Medications

ETU clinicians prescribed standard medications on admission, as per the Médecins Sans Frontières (MSF) EVD clinical management protocol. This included p.r.n. oral metoclopramide for on going nausea and moderate vomiting. The first dose of BCV (200mg; standard adult dosing) was administered on Study Day (SD) 0. Further 100 mg doses of BCV (dissolved in water, due to difficulty swallowing all tablets) were administered as scheduled on SD3 and SD7.

Clinical course

On SD1 the patient was documented to have diarrhoea of mild severity. Diarrhoea persisted from SD2 through SD7, fluctuating between moderate and severe diarrhoea. Moderate vomiting was noted on SD2 and persisted through SD5; the ETU clinicians administered IV fluid replacement (range 2.2 L per 24h to 4.9 L per 24h) in addition to oral rehydration solution, throughout hospitalisation. Bleeding was documented to have occurred on Study Days 0, 3, 4 and 5. From SD1 through SD7, the patient was apyrexial (daily tympanic temperature range 36·6°C-37·3°C), with fluctuating mild-to-moderate tachycardia (range 86-114 bpm). Blood pressure was generally stable, but decreased to 93/63 mmHg on SD6. Tachypnoea worsened on SD6 (40 bpm) and SD7 (48 bpm).

Laboratory tests

Laboratory tests were performed intermittently during hospitalisation, at the discretion of the ETU clinicians. The following abnormalities were detected: hypokalaemia on SD5 only (3·2 mEq/L); hyperuraemia and and hypercreatinaemia on SD7 only (17 mg/dL and 2·7 mg/dL, respectively). Hypoglycaemia was observed on SD6 only (74 mg/dL). EBOV PCR was repeated by ETU clinicians on SD6 and the Ct value had decreased to 19·89.

Outcome

The patient died on SD7. The death was compatible with severe EVD.

***Narrative of Participant 2***

Background

This 12 year-old male patient was admitted to the trial centre and a positive EBOV PCR result was received and the patient enrolled to the trial the same day. The patient’s mother provided consent.

Findings at enrolment

The patient was ambulatory and alert at enrolment. The patient had been symptomatic for seven days prior to admission and the following symptoms were recorded as being present at enrolment: fever; fatigue/general weakness; anorexia; nausea; vomiting of mild severity; diarrhoea of mild severity; sore throat. The patient had the following abnormal vital signs at enrolment: heart rate 102 bpm; respiratory rate 26 bpm.

A Ct value of 20·66 was reported for the EBOV PCR performed following admission to the ETU. A malaria rapid test performed on admission was negative.

Medications

ETU clinicians prescribed standard paediatric medications on admission, as per the MSF EVD clinical protocol. Metoclopramide and loperamide were not prescribed initially. The first weight-adjusted dose of BCV (2 tablets dissolved in 40 ml water, 30 ml administered) was given on SD0. One further dose (1 tablet dissolved in 20 ml water, 15 ml administered) was given as scheduled on SD3. Following discussion with the ETU clinicians and the Trial Operations Group in the UK, administration of the third dose was suspended, due to concerns over worsening diarrhoea. Therefore, BCV was not given on SD7 as scheduled, with an agreed plan to review continuing treatment 24 hours later.

Clinical course

On SD1 (day 8 of illness), the patient was documented to have severe diarrhoea. Moderate to predominantly severe diarrhoea persisted from SD2 through SD7. ETU clinicians prescribed loperamide on SD2, SD4 and SD5. Mild vomiting occurred on SD2, SD3, SD5 and SD6, and metoclopramide was given on SD1 and SD5. The ETU clinicians administered IV fluid replacement (range 3·1L per 24h to 5·8L per 24h) from SD2 through SD7, in addition to oral rehydration solution commenced on admission. Bleeding was not observed at any time. Pyrexia was observed every day except SD1 (daily tympanic temperature, range 37·7°C-39·2°C). Tachycardia persisted throughout admission, generally increasing over time (range 100-180 bpm). Daily morning blood pressure measurements were generally stable and normal from enrolment through SD7. Tachypnoea was observed throughout admission (range 26-44 bpm), and remained >36 bpm from SD3 onwards.

Laboratory tests

Selected laboratory tests were performed intermittently during hospitalisation, at the discretion of the ETU clinicians. The following abnormalities were detected on SD1 and SD3: elevated ALT (711 U/L and 335 U/L, respectively); hypoalbuminaemia (2·5 g/dL and 4·6 g/dL, respectively); elevated CRP (84 and 65 mg/L, respectively). Hypocalcaemia was observed SD1 through SD6. Plasma lactate was measured from SD4 onwards and was consistently elevated (range 2·5-4·8 mmol/L), whereas bicarbonate levels were low when measured from SD4 onwards (range 7·0-10·7 mmol/L). Hypoglycaemia was observed on SD5 and SD6 (59 mg/dL and 39 mg/dL, respectively). Haemoglobin gradually decreased, from 14·3 g/dL on SD1 to 8·5 g/dL on SD7, and haematocrit was ≤38% from SD2 onwards (nadir 25% on SD7). EBOV PCR testing was repeated by ETU clinicians on SD2 and SD6, with associated Ct values of 26·84 and 26·46, respectively.

Outcome

On the morning of SD7 (day 14 of illness), the patient became responsive to painful stimuli only and he died later that day. The death was compatible with severe EVD.

***Narrative of Participant 3***

Background

This 41 year-old female patient was admitted to the trial centre with a positive EBOV PCR result and the patient was enrolled to the trial on the same day.

Findings at enrolment

The patient was alert at enrolment. The patient had been symptomatic for two days and the following symptoms were recorded as being present at enrolment: fatigue/general weakness; joint or muscle pain/aches; anorexia; nausea; vomiting of mild severity; diarrhoea of mild severity; cough; chest pain; abdominal pain. The patient had the following abnormal vital signs at enrolment: heart rate 112 bpm; respiratory rate 28 bpm.

A Ct value of 20·80 was reported for the EBOV PCR performed on admission to the ETU. A malaria rapid test performed on admission was negative.

Medications

ETU clinicians prescribed standard medications on admission, as per the MSF EVD clinical protocol. Loperamide and metoclopramide were commenced at admission, and loperamide was continued through SD3. The first dose of BCV (2 x 100mg tablets) was given on SD0. The next scheduled dose, due to be administered on SD3, was not given due to a rise in plasma creatinine and an associated decrease in the estimated glomerular filtration rate to <15 ml/min/1·73m^2^, which is one of the criteria for exclusion from the trial.

Clinical course

On SD1 the patient was documented to have moderate diarrhoea. Diarrhoea was documented to be severe on SD2 and moderate on SD3. Mild to moderate vomiting occurred on SD1 through SD3. The ETU clinicians administered IV fluid replacement (range 1·5 L per 24h to 2·6 L per 24h) from SD1 through SD3, in addition to oral rehydration solution commenced on admission. Bleeding was not observed at any time. Pyrexia was observed on SD3 only (daily tympanic temperature, 39°C on SD3). Tachycardia persisted through admission, generally increasing over time and peaking on SD3 (range 110-150 bpm). Daily morning blood pressure measurements were generally stable and normal from enrolment through SD3. Tachypnoea was observed throughout admission and worsened over time (range 28-64 bpm).

Laboratory tests

Selected laboratory tests were performed intermittently during hospitalisation, at the discretion of the ETU clinicians. The following abnormalities were detected SD1 through SD3: hyponatraemia (range 129-132 mmol/L); azotaemia (range 40-79 mg/dL); hypercreatinaemia (range 6·5-7·9 mg/dL); hypocalcaemia (nadir 0·9 mmol/L); hyperlactataemia (range 3·9-5·7 mmol/L); low bicarbonate (range 9·4-12·1 mmol/L); hypokalaemia on SD1 only (3·3 mmol/L). Elevated ALT (538 U/L) and hypoalbuminaema (3·1g/dL) were detected when measured on SD2. The ETU clinicians did not repeat EBOV PCR testing.

Outcome

The patient died on SD3, day 5. The death was compatible with severe EVD. It was noted that the patient had significant acute or acute-on-chronic renal impairment.

***Narrative of Participant 4***

Background

This 11 year-old female patient was admitted to the trial centre with a positive EBOV PCR result and the patient was enrolled to the trial on the same day. Consent was obtained from an adult member of the same family. Consent to participate was withdrawn by a more senior adult member of the patient’s family on SD1 and only data collected up to the time of withdrawal are reported.

Findings at enrolment

The patient was alert at enrolment. The patient had been symptomatic for three days and the following symptoms were recorded as being present at enrolment: fever; headache; fatigue/general weakness; joint or muscle pain/aches; anorexia; nausea; difficulty swallowing; diarrhoea of moderate severity; difficulty breathing; bleeding (oral and nasal); conjunctivitis. The patient had the following abnormal vital signs at enrolment: pyrexia (38·3°C); tachycardia (98 bpm); tachypnoea (26 bpm).

A Ct value of 26·74 was reported for the EBOV PCR performed on admission to the ETU. A malaria rapid test performed on admission was negative.

Medications

ETU clinicians prescribed standard paediatric medications on admission, as per the MSF EVD clinical protocol. Loperamide was given on admission to the ETU. The first weight-adjusted dose of BCV (200mg dissolved in 20ml of water, 11ml administered) was given on SD0. Further doses of BCV were not given following withdrawal of consent to participate by a more senior adult member of the same family.

Clinical course

On SD1 the patient was documented to have mild diarrhoea, no vomiting and no bleeding complications. The ETU clinicians administered 2·1L IV fluid replacement over 24 hours, in addition to oral rehydration solution commenced on admission. Daily vital signs monitoring on SD1 revealed the following: temperature of 37°C; heart rate 86 bpm; blood pressure 95/60 mmHg; respiratory rate 26 bpm.

Laboratory tests

Selected laboratory tests were performed at the discretion of ETU clinicians on SD1. The following abnormalities were detected: hyponatraemia (133 mmol/L); hypokalaemia (3·4 mmol/L); azotaemia (23 mg/dL); hyperchloraemia (111 mmol/L); hypocalacaemia (0·8 mmol/L).

Outcome

Clinical outcome data were provided by MSF and it was recorded that the patient died of EVD during their hospitalisation.
