# Supplementary material for: Experimental Treatment of Ebola Virus Disease with Brincidofovir
Source: PLoS One. 2016 Sep 9;11(9):e0162199. doi: 10.1371/journal.pone.0162199 (PMC5017617; doi:10.1371/journal.pone.0162199)
Supplement: S1 Table — (DOCX) [file pone.0162199.s005.docx]

Supporting information

S1 Table: Treatment and clinical progression during admission

|  |  | **Participant** | | | |
| --- | --- | --- | --- | --- | --- |
| **Description**  **[normal range]** | **Study Day** | **1** | **2** | **3** | **4** |
| Brincidofovir  (weight adjusted dose) | Day 0 | Y (200mg) | Y (150mg) | Y (200mg) | Y (110mg) |
|  | Day 1 | N | N | N | **Withdrew** |
|  | Day 2 | N | N | N |  |
|  | Day 3 | Y (100mg) | Y (75mg) | N / **Died** |  |
|  | Day 4 | N | N |  |  |
|  | Day 5 | N | N |  |  |
|  | Day 6 | N | N |  |  |
|  | Day 7 | Y (100mg) / **Died** | N / **Died** |  |  |
| Temperature (^0^C ) | Day 0 | 37·1 | 37·1 | 35·6 | 38·3 |
|  | Day 1 | 36·6 | 37·3 | 35·2 | 37·0 |
|  | Day 2 | 36·6 | 39·0 | 36·2 |  |
|  | Day 3 | 36·4 | 37·7 | 39·0 |  |
|  | Day 4 | 37·2 | 37·1 |  |  |
|  | Day 5 | 37·3 | 39·2 |  |  |
|  | Day 6 | 37·3 | 39·0 |  |  |
|  | Day 7 | 36·8 | 38·4 |  |  |
| Heart rate (beats per minute) | Day 0 | 104 | 102 | 112 | 98 |
|  | Day 1 | 111 | 104 | 110 | 86 |
|  | Day 2 | 90 | 108 | 122 |  |
|  | Day 3 | 101 | 100 | 150 |  |
|  | Day 4 | 86 | 116 |  |  |
|  | Day 5 | 114 | 130 |  |  |
|  | Day 6 | 110 | 180 |  |  |
|  | Day 7 | 100 | 130 |  |  |
| Respiratory rate  (breaths per minute) | Day 0 | 30 | 26 | 28 | 26 |
|  | Day 1 | 24 | 22 | 32 | 26 |
|  | Day 2 | 32 | 28 | 38 |  |
|  | Day 3 | 28 | 36 | 64 |  |
|  | Day 4 | 28 | 40 |  |  |
|  | Day 5 | 30 | 44 |  |  |
|  | Day 6 | 40 | 38 |  |  |
|  | Day 7 | 48 | 44 |  |  |
| Systolic / diastolic blood pressure (mmHg) | Day 0 | 90 / 65 | 118 / 75 | 127 / 96 | 98 / 56 |
|  | Day 1 | 109 / 74 | 105 / 70 | 124 / 103 | 95 / 60 |
|  | Day 2 | 106 / 65 | 104 / 65 | 125 / 94 |  |
|  | Day 3 | 114 / 81 | 111 / 70 | 131 / 108 |  |
|  | Day 4 | 111 / 73 | 123 / 83 |  |  |
|  | Day 5 | 105 / 72 | 109 / 89 |  |  |
|  | Day 6 | 93 / 63 | 100 / 59 |  |  |
|  | Day 7 |  | 119 / 64 |  |  |
| Vomiting | Day 0 | Y (++) | Y (+) | Y (+) | N |
|  | Day 1 | Y (+) | N | Y (+) | N |
|  | Day 2 | Y (++) | Y (+) | Y (++) |  |
|  | Day 3 | Y (++) | Y (+) | Y (+) |  |
|  | Day 4 | Y (++) | N |  |  |
|  | Day 5 | Y (++) | Y (+) |  |  |
|  | Day 6 | N | Y (+) |  |  |
|  | Day 7 | N | N |  |  |
| Diarrhoea | Day 0 | N | Y (+) | Y (+) | Y (++) |
|  | Day 1 | Y (+) | Y (+++) | Y (++) | Y (+) |
|  | Day 2 | Y (++) | Y (+++) | Y (+++) |  |
|  | Day 3 | Y (+++) | Y (++) | Y (++) |  |
|  | Day 4 | Y (++) | Y (+++) |  |  |
|  | Day 5 | Y (++) | Y (+++) |  |  |
|  | Day 6 | Y (+++) | Y (+++) |  |  |
|  | Day 7 | Y (++) | Y (++) |  |  |
| Bleeding | Day 0 | Y | N | N | Y |
|  | Day 1 | N | N | N | N |
|  | Day 2 | N | N | N |  |
|  | Day 3 | Y | N | N |  |
|  | Day 4 | Y | N |  |  |
|  | Day 5 | Y | N |  |  |
|  | Day 6 | N | N |  |  |
|  | Day 7 | N | N |  |  |
| Oral Rehydration Solution (ORS) and intravenous (IV) fluid therapy (IV volume litres) | Day 0 | ORS | ORS | ORS / IV | IV |
|  | Day 1 | IV (2·2) | ORS | ORS / IV (2·6) | ORS / IV (2·1) |
|  | Day 2 | ORS / IV (2·2) | ORS / IV (3·1) | ORS / IV (1·5) |  |
|  | Day 3 | IV (3·3) | IV (4·3) | ORS / IV (1·5) |  |
|  | Day 4 | IV (4·9) | ORS / IV (5·8) |  |  |
|  | Day 5 | IV (3·3) | IV (2·5) |  |  |
|  | Day 6 | IV (3·6) | ORS / IV (1·9) |  |  |
|  | Day 7 | IV (4·8) | ORS / IV (2·1) |  |  |
| Ebolavirus PCR cycle threshold (Ct) | Day 0 | 22·7 | 20·66 | 20·8 | 26·74 |
|  | Day 1 |  |  |  |  |
|  | Day 2 |  | 26·84 |  |  |
|  | Day 3 |  |  |  |  |
|  | Day 4 |  |  |  |  |
|  | Day 5 |  |  |  |  |
|  | Day 6 | 19·89 | 26·46 |  |  |
|  | Day 7 |  |  |  |  |
| Sodium (mmol/L ) [i-STAT reference range: 138-146 mmol/L. Piccolo reference range: 128-145 mmol/L)] | Day 0 | 136 | 129 | 134 | 129 |
|  | Day 1 |  | 131 | 129 | 133 |
|  | Day 2 | 138 | 127 | 133 |  |
|  | Day 3 | 140 | 131 | 132 |  |
|  | Day 4 |  | 133^P^ |  |  |
|  | Day 5 | 138 | 125 |  |  |
|  | Day 6 | 138 | 130 ^P^ |  |  |
|  | Day 7 | 136 ^P^ | 125 |  |  |
| Potassium (mmol/L) [i-STAT reference range: 3·5-4·9 mmol/L. Piccolo reference range: 3·6-5·1 mmol/L)] | Day 0 | 4·1 |  | 2·8 | 7·8 |
|  | Day 1 |  | 2·7 | 3.3 | 3·4 |
|  | Day 2 | 3.7 | 2·8 | 4.3 ^P^ |  |
|  | Day 3 | 3·7 | 3·2 | 3·7 |  |
|  | Day 4 |  | 3·3 |  |  |
|  | Day 5 | 3·2 | 4·1 ^P^ |  |  |
|  | Day 6 | 3·6 | 3·3 |  |  |
|  | Day 7 | 4·0 | 3·3 |  |  |
| Chloride (mmol/L ) [i-STAT reference range: 98-109 mmol/L. Piccolo reference range: 98-108 mmol/L)] | Day 0 | 102 ^P^ | 96 | 103 | 106 |
|  | Day 1 |  | 99 | 106 | 111 |
|  | Day 2 | 106 | 100 | 97 ^P^ |  |
|  | Day 3 | 108 | 105 | 105 |  |
|  | Day 4 |  | 111 |  |  |
|  | Day 5 | 107 | 112 |  |  |
|  | Day 6 | 108 | 110 |  |  |
|  | Day 7 | 106 | 102 |  |  |
| Calcium (mmol/L) [i-STAT ionised calcium reference range: 1·2-1·32 mmol/L. Piccolo calcium reference range: 8·0-10·3 mg/dl)] | Day 0 | 10·3 ^P^ | 7·3 ^P^ | 1·1 | 0·9 |
|  | Day 1 |  | 1.0 | 0·9 | 0·8 |
|  | Day 2 | 1·3 | 0·9 | 7·6 ^P^ |  |
|  | Day 3 | 1·4 | 1·0 | 0·9 |  |
|  | Day 4 |  | 1·1 |  |  |
|  | Day 5 | 1·4 | 0·8 |  |  |
|  | Day 6 | 1·3 | 0·9 |  |  |
|  | Day 7 | 1·1 | 0·9 |  |  |
| Bicarbonate (mmol/L) [i-STAT reference range: 24-29 mmol/L. Piccolo reference range: 18-33 mmol/L)] | Day 0 |  | 14·2 |  | 19·3 |
|  | Day 1 |  |  | 12·1 |  |
|  | Day 2 |  |  | 9·4 |  |
|  | Day 3 |  |  | 11·1 |  |
|  | Day 4 |  | 10·7 |  |  |
|  | Day 5 |  | 9·2 ^P^ |  |  |
|  | Day 6 |  | 7 |  |  |
|  | Day 7 |  | 10·4 |  |  |
| Blood urea nitrogen (mg/dL) [i-STAT reference range: 8-26 mg/dL. Piccolo reference range: 7-22 mg/dL)] | Day 0 | 7 ^P^ | 5 | 37 ^P^ | 27 |
|  | Day 1 |  | 5 | 49 | 23 |
|  | Day 2 | 3 | 11 | 40 ^P^ |  |
|  | Day 3 | 2·5 | 11·0 | 79·0 |  |
|  | Day 4 |  | 3 |  |  |
|  | Day 5 | 2·5 | 8·0 |  |  |
|  | Day 6 | 6 | 12 |  |  |
|  | Day 7 | 17 |  |  |  |
| Creatinine (mg/dL) [i-STAT reference range: 0·6-1·3 mmol/L. Piccolo reference range: 0.6-1·2 mg/dL)] | Day 0 | 1 ^P^ | 0·7 | 6·8 ^P^ | 1 |
|  | Day 1 |  | 0·6 | 8·8 | .9 |
|  | Day 2 | 0·8 | 0·7 | 6·5 ^P^ |  |
|  | Day 3 | 0·9 | 0·6 ^P^ | 7·9 |  |
|  | Day 4 |  | 0·6 |  |  |
|  | Day 5 | 0·9 | 0·6 ^P^ |  |  |
|  | Day 6 | 1·2 | 0·7 |  |  |
|  | Day 7 | 2·7 | 0·7 |  |  |
| ALT/SGPT (U/L) [Piccolo normal range 10-47 U/L] | Day 0 | 96 ^P^ | 750 ^P^ |  |  |
|  | Day 1 |  | 711 ^P^ |  |  |
|  | Day 2 |  |  | 538 ^P^ |  |
|  | Day 3 |  | 335 ^P^ |  |  |
|  | Day 4 |  |  |  |  |
|  | Day 5 |  |  |  |  |
|  | Day 6 |  |  |  |  |
|  | Day 7 |  |  |  |  |
| Lactate (mmol/L) [i-STAT reference range 0·9-1·70 mmol/L] | Day 0 |  |  |  |  |
|  | Day 1 |  |  | 4·4 |  |
|  | Day 2 |  |  | 3·9 |  |
|  | Day 3 |  |  | 5·7 |  |
|  | Day 4 |  | 4·1 |  |  |
|  | Day 5 |  | 3.4 ^P^ |  |  |
|  | Day 6 |  | 2·5 |  |  |
|  | Day 7 |  | 4·8 |  |  |
| Glucose (mg/dL) [i-STAT reference range: 70-105 mg/dL. Piccolo reference range: 73-118 mg/dL)] | Day 0 | 111 ^P^ | 91 | 107 ^P^ | 158 ^P^ |
|  | Day 1 |  | 189 ^P^ | 112 |  |
|  | Day 2 | 111 | 86 | 106 ^P^ |  |
|  | Day 3 | 136 | 170 ^P^ | 93 |  |
|  | Day 4 |  | 94 |  |  |
|  | Day 5 | 111 | 59 ^P^ |  |  |
|  | Day 6 | 74 | 39 |  |  |
|  | Day 7 | 161 | 147 |  |  |
| Haemoglobin (g/dL) [i-STAT reference range 12-17 g/dL] | Day 0 |  | 13·3 | 17·3 | 15·6 |
|  | Day 1 |  | 14·3 | 18·0 | 14·6 |
|  | Day 2 | 16·0 | 12·9 |  |  |
|  | Day 3 | 15·6 | 12·2 | 16·0 |  |
|  | Day 4 |  | 12·6 |  |  |
|  | Day 5 | 16·0 | 10·2 |  |  |
|  | Day 6 | 15·6 | 8·8 |  |  |
|  | Day 7 | 16·3 | 8·5 |  |  |
| Haematocrit (%) [i-STAT reference range 38-51%] | Day 0 | 42 | 53 |  | 43 |
|  | Day 1 |  | 42 | 53 | 43 |
|  | Day 2 | 47 | 38 |  |  |
|  | Day 3 | 46 | 36 | 47 |  |
|  | Day 4 |  | 37 |  |  |
|  | Day 5 | 47 | 30 |  |  |
|  | Day 6 | 46 | 26 |  |  |
|  | Day 7 | 48 | 25 |  |  |
